# Genome of the Netherlands population-specific imputations identify an *ABCA6* variant associated with cholesterol levels

**DOI:** 10.1038/ncomms7065

**Published:** 2015-03-09

**Authors:** Elisabeth M. van Leeuwen, Lennart C. Karssen, Joris Deelen, Aaron Isaacs, Carolina Medina-Gomez, Hamdi Mbarek, Alexandros Kanterakis, Stella Trompet, Iris Postmus, Niek Verweij, David J. van Enckevort, Jennifer E. Huffman, Charles C. White, Mary F. Feitosa, Traci M. Bartz, Ani Manichaikul, Peter K. Joshi, Gina M. Peloso, Patrick Deelen, Freerk van Dijk, Gonneke Willemsen, Eco J. de Geus, Yuri Milaneschi, Brenda W.J.H. Penninx, Laurent C. Francioli, Androniki Menelaou, Sara L. Pulit, Fernando Rivadeneira, Albert Hofman, Ben A. Oostra, Oscar H. Franco, Irene Mateo Leach, Marian Beekman, Anton J.M. de Craen, Hae-Won Uh, Holly Trochet, Lynne J. Hocking, David J. Porteous, Naveed Sattar, Chris J. Packard, Brendan M. Buckley, Jennifer A. Brody, Joshua C. Bis, Jerome I. Rotter, Josyf C. Mychaleckyj, Harry Campbell, Qing Duan, Leslie A. Lange, James F. Wilson, Caroline Hayward, Ozren Polasek, Veronique Vitart, Igor Rudan, Alan F. Wright, Stephen S. Rich, Bruce M. Psaty, Ingrid B. Borecki, Patricia M. Kearney, David J. Stott, L. Adrienne Cupples, The Genome of the Netherlands Consortium, Pieter B.T. Neerincx, Clara C. Elbers, Pier Francesco Palamara, Itsik Pe'er, Abdel Abdellaoui, Wigard P. Kloosterman, Mannis van Oven, Martijn Vermaat, Mingkun Li, Jeroen F.J. Laros, Mark Stoneking, Peter de Knijff, Manfred Kayser, Jan H. Veldink, Leonard H. van den Berg, Heorhiy Byelas, Johan T. den Dunnen, Martijn Dijkstra, Najaf Amin, K. Joeri van der Velde, Jessica van Setten, Mathijs Kattenberg, Barbera D.C. van Schaik, Jan Bot, Isaäc J. Nijman, Hailiang Mei, Vyacheslav Koval, Kai Ye, Eric-Wubbo Lameijer, Matthijs H. Moed, Jayne Y. Hehir-Kwa, Robert E. Handsaker, Shamil R. Sunyaev, Mashaal Sohail, Fereydoun Hormozdiari, Tobias Marschall, Alexander Schönhuth, Victor Guryev, H. Eka D. Suchiman, Bruce H. Wolffenbuttel, Mathieu Platteel, Steven J. Pitts, Shobha Potluri, David R. Cox, Qibin Li, Yingrui Li, Yuanping Du, Ruoyan Chen, Hongzhi Cao, Ning Li, Sujie Cao, Jun Wang, Jasper A. Bovenberg, J. Wouter Jukema, Pim van der Harst, Eric J. Sijbrands, Jouke-Jan Hottenga, Andre G. Uitterlinden, Morris A. Swertz, Gert-Jan B. van Ommen, Paul I.W. de Bakker, P. Eline Slagboom, Dorret I. Boomsma, Cisca Wijmenga, Cornelia M. van Duijn

**Affiliations:** 1 Department of Epidemiology, Erasmus Medical Center, Rotterdam 3000 CA, The Netherlands; 2 Department of Molecular Epidemiology, Leiden University Medical Center, Leiden 2300 RC, The Netherlands; 3 Department of Epidemiology and Internal Medicine, Erasmus Medical Center, Rotterdam 3000 CA, The Netherlands; 4 Department of Biological Psychology, VU University Amsterdam and EMGO+ Institute for Health and Care Research, Amsterdam 1081BT, The Netherlands; 5 Department of Genetics, Genomics Coordination Center, University of Groningen, University Medical Center Groningen, Groningen 9700 RB, The Netherlands; 6 Department of Cardiology, Leiden University Medical Center, Leiden 2300 RC, The Netherlands; 7 Department of Gerontology and Geriatrics, Leiden University Medical Center, Leiden 2300 RC, The Netherlands; 8 Department of Cardiology, University Medical Center Groningen, University of Groningen, Groningen 9700 RB, The Netherlands; 9 BioAssist, Netherlands Bioinformatics Center, Nijmegen 6500 HB, The Netherlands; 10 MRC Human Genetics Unit, MRC IGMM, University of Edinburgh, Edinburgh EH4 2XU, UK; 11 Department of Biostatistics, Boston U School of Public Health, Boston, Massachusetts 02118, USA; 12 Department of Genetics, Washington University School of Medicine, St Louis, Missouri 63108, USA; 13 Department of Biostatistics and Medicine, University of Washington, Seattle, Washington 98101, USA; 14 Department of Public Health Sciences, Center for Public Health Genomics, University of Virginia, Charlottesville, Virginia 22908, USA; 15 Centre for Population Health Sciences, University of Edinburgh, Edinburgh, Scotland EH8 9AG, UK; 16 Center for Human Genetic Research, Massachusetts General Hospital, Boston, Massachusetts 02176, USA; 17 Department of Psychiatry, VU University Medical Center Amsterdam/GGZinGeest, EMGO+ Institute for Health and Care Research, Neuroscience Campus Amsterdam, Amsterdam 1081HL, The Netherlands; 18 Department of Medical Genetics, Center for Molecular Medicine, University Medical Center Utrecht, Utrecht 3584 CG, The Netherlands; 19 Department of Clinical Genetics, Erasmus Medical Center, Rotterdam 3000 CA, The Netherlands; 20 Department of Genetical Statistics, Leiden University Medical Center, Leiden 2300 RC, The Netherlands; 21 Division of Applied Health Sciences, University of Aberdeen, Aberdeen AB25 2ZD, UK; 22 Centre for Genomic and Experimental Medicine, MRC IGMM, University of Edinburgh, Edinburgh EH4 2XU, UK; 23 BHF Glasgow Cardiovascular Research Centre, Faculty of Medicine, University of Glasgow, Glasgow G12 8QQ, UK; 24 Institute of Cardiovascular and Medical Sciences, University of Glasgow, Glasgow G12 8QQ, UK; 25 Department of Pharmacology and Therapeutics, University College Cork, Cork, Ireland; 26 Department of Medicine, University of Washington, Seattle, Washington 98101, USA; 27 Institute for Translational Genomics and Population Sciences, Los Angeles BioMedical Research Institute at Harbor-UCLA Medical Center, Torrance, California 90502, USA; 28 Department of Genetics, University of North Carolina, Chapel Hill, North Carolina NC 27599, USA; 29 Department of Public Health, Faculty of Medicine, University of Split, Split 21000, Croatia; 30 Department of Medicine and Epidemiology, University of Washington, Seattle, Washington 98101, USA; 31 Department of Genetics and Biostatistics, Washington University School of Medicine, St Louis, Missouri 63108, USA; 32 Framingham Heart Study, Framingham, Massachusetts 01702, USA; 33 Department of Internal Medicine, Erasmus Medical Center, Rotterdam 3000 CA, The Netherlands; 34 Department of Human Genetics, Leiden University Medical Center, Leiden P.O. Box 9600, 2300 RC, The Netherlands; 35 Department of Epidemiology, Julius Center for Health Sciences and Primary Care, University Medical Center Utrecht, Utrecht 3584 CG, The Netherlands; 36 Department of Biological Psychology, VU University Amsterdam, Amsterdam 1081BT, The Netherlands; 37 Department of Genetics, University of Groningen, University Medical Center Groningen, Groningen 9700 RB, The Netherlands; 38 Department of Computer Science, Columbia University, New York, NY 10027-7003, USA; 39 Department of Systems Biology, Columbia University, New York, NY 10032, USA; 40 Department of Forensic Molecular Biology, Erasmus Medical Center, Rotterdam 3000 CA, The Netherlands; 41 Leiden Genome Technology Center, Department of Human Genetics, Leiden University Medical Center, Leiden 2300 RC, The Netherlands; 42 Department of Evolutionary Genetics, Max Planck Institute for Evolutionary Anthropology, Leipzig, 4103, Germany; 43 Forensic Laboratory for DNA Research, Department of Human Genetics, Leiden University Medical Center, Leiden, 2300 RC, The Netherlands; 44 Department of Neurology, University Medical Center Utrecht, Utrecht, 3584 CG, The Netherlands; 45 Bioinformatics Laboratory, Department of Clinical Epidemiology, Biostatistics and Bioinformatics, Amsterdam Medical Center, Amsterdam 1090 GE, The Netherlands; 46 SURFsara, Science Park, Amsterdam 1098 XG, The Netherlands; 47 The Genome Institute, Washington University, St. Louis, MO 98101, USA; 48 Department of Human Genetics, Radboud University Nijmegen Medical Centre, Nijmegen 6500 HB, The Netherlands; 49 Broad Institute of Harvard and MIT, Cambridge, MA 2142, USA; 50 Department of Genetics, Harvard Medical School, Boston, MA 2115, USA; 51 Division of Genetics, Brigham and Women's Hospital, Harvard Medical School, Boston, MA 2115, USA; 52 Department of Genome Sciences, University of Washington, Seattle, WA 98101, USA; 53 Centrum voor Wiskunde en Informatica, Life Sciences Group, Amsterdam 1098 XG, The Netherlands; 54 European Research Institute for the Biology of Ageing, University Medical Center Groningen, University of Groningen, Groningen 9700 RB, The Netherlands; 55 Department of Endocrinology, University Medical Center Groningen, Groningen 9700 RB, The Netherlands; 56 Rinat-Pfizer Inc, South San Francisco, CA 10017, USA; 57 BGI-Shenzhen, Shenzhen 518083, China; 58 BGI-Europe, Copenhagen DK-1870, Denmark; 59 Department of Biology, University of Copenhagen, Copenhagen 2100, Denmark; 60 The Novo Nordisk Foundation Center for Basic Metabolic Research, University of Copenhagen, Copenhagen 2100, Denmark; 61 Legal Pathways Institute for Health and Bio Law, Aerdenhout, The Netherlands; 62 A full list of consortium members appears at the end of the paper.

## Abstract

Variants associated with blood lipid levels may be population-specific. To identify low-frequency variants associated with this phenotype, population-specific reference panels may be used. Here we impute nine large Dutch biobanks (~35,000 samples) with the population-specific reference panel created by the Genome of the Netherlands Project and perform association testing with blood lipid levels. We report the discovery of five novel associations at four loci (*P* value <6.61 × 10^−4^), including a rare missense variant in *ABCA6* (rs77542162, p.Cys1359Arg, frequency 0.034), which is predicted to be deleterious. The frequency of this *ABCA6* variant is 3.65-fold increased in the Dutch and its effect (*β*_LDL-C_=0.135, *β*_TC_=0.140) is estimated to be very similar to those observed for single variants in well-known lipid genes, such as *LDLR*.

## Abstract (web summary)

Frequencies of rare variants fluctuate over populations, hampering gene discovery. Here the authors use a population-specific reference panel, the Genome of the Netherlands, to discover four novel loci involved in lipid metabolism, including an exonic variant in *ABCA6*.

## Graphical abstract

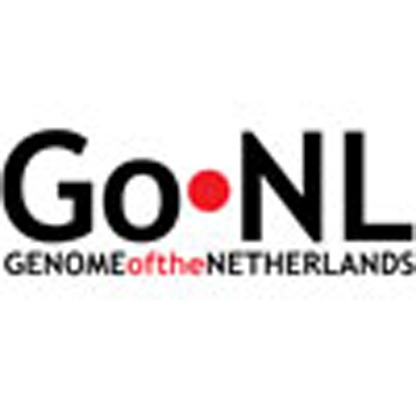

Genome-wide association studies (GWAS) have identified a large number of loci associated with blood lipid levels and analysis suggest there are additional susceptibility loci that have not yet been discovered^1^,^2^,^3^. Despite the fact that rare functional variants are known to play a major role in lipid metabolism^1^,^2^,^3^, there has been limited success in finding such variants in population-based studies using next-generation sequencing. Even if the effect of these variants is expected to be larger than that of common variants, the sample size needed to detect these rare or low-frequency variants increases dramatically with variant rarity. As the frequency of rare variants may increase in certain populations because of drift and founder effects^4^, the power of searches for rare functional variants may improve by the use of reference sets specific to distinct populations. Such references allow for better quality imputation of rare variants especially those with increased frequency in the population of interest^3^,^5^,^6^. Previous studies have successfully detected rare variants by imputation into larger sets of individuals in isolated populations followed by association testing to detect variants associated with the trait of interest^7^,^8^,^9^.

Here we describe an imputation-based GWAS for circulating lipid levels using a custom-built reference panel for the Dutch population (Genome of the Netherlands, GoNL, http://www.nlgenome.nl/), in which the whole genomes of 250 parent–offspring trios were sequenced at ~13 × coverage^5^,^6^. Owing to the trio design, the phasing quality of the reference panel was better than that of the 1000 Genomes (1-kG) Phase 1 panel. In this study we show that using this population-specific reference panel we were able to identify five novel associations at four loci.

## Results

Nine large Dutch epidemiological cohorts (comprising 36,000 samples in total) were imputed with the GoNL reference panel (~19.5 million single-nucleotide polymorphisms (SNPs)) on an identical protocol^6^,^10^. All cohorts conducted association analysis on the imputed variants assuming an additive genetic effect on high-density lipoprotein cholesterol (HDL-C), low-density lipoprotein cholesterol (LDL-C), total cholesterol (TC) and triglyceride (TG) levels (Methods, Supplementary Methods and Supplementary Table 1), and the results were meta-analysed. We used conditional analysis implemented in GCTA^11^ to identify variants associated independently with lipid levels.

**Figure 1:**
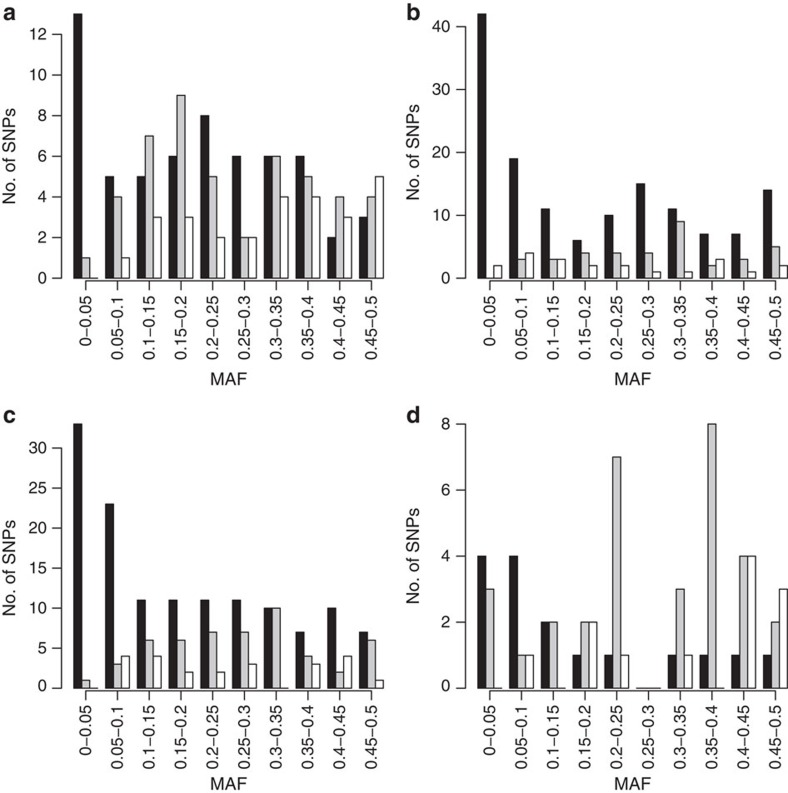
Identified variants for plasma lipid levels. Distribution of the variants identified by conditional analysis implemented by GCTA to be independently associated with the lipid traits (**a**) HDL-C (60 variants), (**b**) LDL-C (142 variants), (**c**) TC (134 variants) and (**d**) TG (16 variants)) over MAF bins after meta-analysis of discovery cohorts (black). The histograms also include loci identified in ref. 1 (grey) and ref. 2 (white).

Both rare (minor allele frequency (MAF) <0.01), low (0.01<MAF<0.05) and common variants (MAF>0.05) were associated with HDL-C (*N*=60 variants), LDL-C (*N*=142 variants), TC (*N*=134 variants) and TG (*N*=16 variants) in both known and novel loci (Methods, Supplementary Tables 2–5 and Supplementary Fig. 1). In Fig. 1 we compare the allele frequencies that reach genome-wide significance in the GCTA analysis (*P* value <5 × 10^−8^) to those reported in refs 1, 2 (Fig. 1). The majority of the known HDL-C (31 of 45, 68.9%), LDL-C (24 of 34, 70.6%), TC (33 of 48, 68.6%) and TG (13 of 30, 43.3%) loci described in ref. 1 replicated at a *P* value <3.18 × 10^−4^ (Bonferroni correction based on 157 variants; Methods, Supplementary Figs 2 and 3 and Supplementary Tables 6–7). We also confirmed several of the HDL-C (6 of 27, 22.2%), LDL-C (7 of 21, 33.3%), TC (4 of 23, 17.4%) and TG (1 of 12, 8.3%) loci described in ref. 2 at a *P* value <6.02 × 10^−4^ (Bonferroni correction based on 83 variants) despite a sample size of ~20% of the other studies.

To identify novel loci associated with blood lipid levels, we selected from the list of variants identified by GCTA, those variants located more than 1 Mb away from previously identified loci. This resulted in six novel associations at five loci (Methods, Tables 1 and 2 and Supplementary Table 8). The five loci are not in linkage disequilibrium (LD) with previously described GWAS loci (Methods and Supplementary Table 9). Conditional analysis in the discovery cohorts showed that these new variants were independent from previously identified loci (Supplementary Table 10 and Supplementary Fig. 4). Of the five loci, three (rs149580368, rs77542162 and rs144984216) have an increased frequency in GoNL compared with 1-kG (Phase 1 integrated release v3, April 2012, all ancestries; Table 1), suggesting that there may have been genetic drift in the Dutch population for these loci^4^. Yet, as each of these loci has a MAF>0.005, we assumed that these alleles also segregate in other populations of European descent^4^, such as those of the Cohorts for Heart and Aging Research in Genomic Epidemiology (CHARGE) consortium. Therefore, we set out replication in independent samples from the CHARGE cohorts using the 1-kG reference panel (Phase 1 integrated release v3, April 2012, all ancestries). We were able to replicate five out of the six variants using the Bonferroni-corrected *P* value threshold of 8.33 × 10^−3^ (Table 2, Methods and Supplementary Table 11).

Of the replicated variants, rs77542162 is the most interesting variant. This missense variant is associated with both LDL-C and TC (Supplementary Figs 5 and 6) and is located on chromosome 17 within the *ABCA6* gene (ATP-binding cassette, subfamily A (ABC1), member 6). The frequency of this variant is 1.31-fold higher in the discovery cohorts than in the replication cohorts and even 3.65-fold higher in the GoNL population than in the 1-kG population. This missense variant changes the amino acid cysteine into arginine at position 1359 (Cys1359Arg) and is predicted to be damaging for the structure and function of the protein by Polyphen2 (ref. 12), MutationTaster^13^ and LRT^14^. The effect size of rs77542162 (*β*_LDL-C_=0.135 and *β*_TC_=0.140) is very similar to those observed for other single variants in well-known lipid genes, such as *LDLR* and *CETP*, as reported in ref. 1. The membrane-associated protein encoded by this gene is a member of the superfamily of ATP-binding cassette (ABC) transporters that transport various molecules across extra- and intracellular membranes. This protein is a member of the ABC1 subfamily, which is the only major ABC subfamily found exclusively in multicellular eukaryotes. *ABCA6* is clustered with four other ABC1 family members on chromosome 17q24 and appears to play a role in macrophage lipid homeostasis.

One other replicated variant, rs149580368, is also enriched with a 1.92-fold increase in frequency in the Dutch population compared with the 1-kG population. This intergenic variant (Supplementary Fig. 7), without a significant *cis-*eQTL effect, is located between the protein-coding genes *C17orf105* (chromosome 17 open reading frame 105) and *MPP3* (membrane protein, palmitoylated 3). Two replicated variants have similar frequencies in the GoNL and 1-kG reference sets: rs4752801 (Supplementary Fig. 8), an new intergenic variant with a high frequency (MAF=0.355) that is located in a region previously identified^1^, and rs117162033 (Supplementary Fig. 9), an intronic variant in the myosin F (*MYO1F*)-coding gene. *C17orf15, MPP3* and *MYO1F* have no known impact on lipid levels. As the imputation quality of rs117162033 is lower than the other variants, we validated the imputation of this variant using the same approach as published in ref. 15. We compared in a random sample of 65 participants of the GoNL reference panel their sequence and best-guess GoNL-imputed genotypes and found that the concordance was 100% (all participants were correctly imputed). The association between TG and the intronic variant in the *MYO1F* gene is remarkable because of the low frequency of the variant. This confirms the conclusions as published before about the GoNL reference panel, that the trio-based phasing contributed significantly to the imputation quality of rare variants^5^.

In this current study, the GoNL reference panel was used for imputations of the discovery cohorts and the 1-kG reference panel for the imputation of the replication cohorts. Although it would be interesting to impute with a combined reference panel of both the GoNL data, the 1-kG data and other sequence data, this effort is ongoing.

This study shows that the imputation of a population-specific reference panel into large epidemiological cohorts can reveal both low-frequency and rare variants associated with blood lipid levels using classical association testing approaches. The three variants with increased frequency in the Dutch population as compared with the 1-kG population include a rare, predicted to be deleterious missense variant in *ABCA6,* which has increased frequency 3.65 times larger in the Dutch population. The effect of this variant is comparable to that of variants in the *LDLR* gene, a gene for which several population-based screening programmes have been initiated. Our findings suggest that next-generation-sequencing effort may yield clinically relevant findings. Our paper further shows that next-generation-sequencing efforts in *specific homogeneous* populations as the Dutch may yield clinically relevant findings *worldwide*.

## Methods

### Study descriptions

The descriptions of the including cohorts can be found in the Supplementary Methods. A written informed consent was obtained from all study participants for all cohorts and local ethical committees at participating institutions approved individual study protocols.

### Study samples and phenotypes

A summary of the details of both the discovery and replication cohorts participating in this study can be found in Supplementary Tables 1 and 12.

Only samples of Dutch ancestry were used in the discovery cohorts; the samples in the replication cohorts are from various ancestries (see Supplementary Table 12). In all studies, except MESA Whites, all individuals who used lipid-lowering medication at the time the lipid levels were measured, were excluded. In MESA Whites, the total cholesterol values for individuals on lipid-lowering medication were divided by 0.8. In all studies except for LLS and PREVEND, the subjects were fasting when the lipid levels were measured. In LLS all samples were non-fasted and in PREVEND 2.99% were non-fasted. The LDL-C levels were measured within the ERF, Croatia-Korcula, Croatia-Split, Croatia-Vis, FamHS and Lifelines cohorts, within the other cohorts the Friedewald equation was used to calculate the LDL-C levels^16^.

The lipid measurements were adjusted for sex, age and age^2^ in all cohorts. Various methods were used to account for family relationships: in ERF grammar-gamma, GenABEL version 1.7.6 (refs 17, 18) was used; in the Croatia-Korcula, Croatia-Split, Croatia-Vis and Generation Scotland cohorts mmscore (GenABEL)^17^ was used; and in LLS, qt-assoc was used. In CHS the clinic was used as extra covariate, in Lifelines PC1 and PC2, in FamHS the field centre, the genotyping array (Illumina 550 k, 610 k and 1 M), PC5 only for TC and PC1 only for LDL, in FHS the cohort (offspring and third generation) and PCs, in MESA Whites 2 PCs and study site, in NTR-NESDA PCs and chip effect, in ORCADES the genotyping array and PC1, PC2 and PC3, in PROSPER-Dutch only PC1 and in both PROSPER-Scottish and PROSPER-Irish PC1-PC4.

### Genotyping and imputations

Detailed information about genotyping and imputations per cohort can be found in the Supplementary Methods. In summary, all cohorts were genotyped using commercially available Affymetrix or Illumina genotyping arrays, or custom Perlegen arrays. Quality control was performed independently for each study. To facilitate meta-analysis, each replication cohort performed genotype imputation using IMPUTE^19^ or Minimac^20^ with reference to the GoNL project data for the discovery cohorts and with reference to the 1-kG project data for the replication cohorts.

### GWAS in all discovery cohorts

All nine discovery cohorts ran separate the genome-wide association study for each of the four traits: HDL-C, LDL-C, TC and TG. Supplementary Table 13 shows the genomic control factor *λ* per trait per cohort and Supplementary Figs 10–13 show the *λ* per MAF bin per trait per cohort. We therefore used only the SNPs with a *R*^2^>0.3, *R*^2^<1.1 and expected minor allele count (expMAC=2 × MAF × *R*^2^·sample size) >10. Most inflations are observed within the ERF study, especially in the lowest-frequency variants, which is probably caused by the family structure in this cohort.

### Meta-analysis of discovery cohorts

The association results of all studies were combined and the s.e.-based weights were calculated using METAL^21^. This tool also applies genomic control by automatically correcting the test statistics to account for small amounts of population stratification or unaccounted relatedness. METAL also allows for heterogeneity. We used the following filters: 0.3<*R*^2^<1.1 and expMAC>10.

After meta-analyses of all available variants, we excluded the variants that are not present in at least six of the nine cohorts. We also excluded all variants that are labelled as being in the inaccessible genome, since the quality of those SNPs cannot be guaranteed^22^. The remaining variants per trait, see Supplementary Table 14, were used to create Manhattan plots and QQ plots, see Supplementary Figs 14 and 15. The meta-analysis resulted in 1,905 SNPs with a *P* value less than 5 × 10^−8^ for HDL-C, 2,626 SNPs for LDL-C, 3,133 SNPs for TC and 1,310 for TG.

### Confirmation of known loci

Previously, Teslovich *et al*^1^ and Willer *et al*^2^ identified 157 loci associated with one of more of the lipids. Teslovich *et al*^1^ identified 47, 37, 52 and 32 loci to be associated with HDL-C, LDL-C, TC and TG, respectively. The positions of these loci were reported on human genome build 36; we therefore lifted these positions over to human genome build 37 and checked the association results after the meta-analysis of all discovery cohorts. The effect size of these loci was reported in mg dl^−1^, whereas in this study we use mmol l^−1^. We therefore multiplied the effect size for the loci associated with TG with 0.0259 and the other loci with 0.011. Supplementary Fig. 2 and Supplementary Table 6 show the comparison per trait of our meta-analysis of all discovery cohorts with the results of the meta-analysis in ref. 1. We did the same for the loci identified in ref. 2, see Supplementary Fig. 3 and Supplementary Table 7. The effect size of these loci could not be compared with our results, since trait residuals within each study participating in the meta-analysis in ref. 2 were adjusted for sex and age^2^ and subsequently quantile normalized. Their GWAS was performed with the inverse normal transformed trait values.

### Selection of independent variants

In order to select only associated variants that were independent of previous findings, we used the GCTA tool^11^. This tool performs a stepwise selection procedure to select multiple associated SNPs by a conditional and joint analysis approach using summary-level statistics from a meta-analysis and LD corrections between SNPs estimated from the GoNL reference panel, release 4. This analysis revealed 60 independent variants associated with HDL-C, 142 independent variants associated with LDL-C, 134 independent variants associated with TC and 16 independent variants associated with TG. By using this approach, we were able to identify additional independent variants in known loci. Figure 1 shows that we identified both common and rare variants and more rare variants compared with refs 1, 2. There is an overlap between the genome-wide significant SNPs of the different traits, and also between the independent SNPs of the different traits, as shown in Supplementary Fig. 1.

### Identification of potential novel variants

To identify potential novel variants, we first excluded all variants within 1 Mb of a known loci from refs 1, 2. Since the number of loci associated with the four traits differ, we end up with 7,946,245 SNPs for HDL-C, 8,014,693 SNPs for LDL-C, 7,923,530 SNPs for TC and 7,468,790 SNPs for TG. For all traits we do find some genome-wide significant loci, see Supplementary Figs 16 and 17. We used the GCTA tool to select only those variants that are independently associated with the lipid trait. This analysis revealed two novel independent variants associated with HDL-C, one novel independent variant associated with LDL-C, two novel independent variants associated with TC and one novel independent variants associated with TG, see Supplementary Table 8 and Supplementary Fig. 18. We used PLINK to test whether these six variants are in LD with the known loci from refs 1, 2. None of the six variants are in LD with known loci associated with the same trait on the same chromosome (*R*^2^<0.14).

### Replication of potential novel variants

The six potential novel loci were replicated in 11 cohorts: CHS, Croatia-Korcula, Croatia-Split, Croatia-Vis, FamHS, FHS, Generation Scotland, MESA Whites, ORCADES, PROSPER-Scottish and PROSPER-Irish. The association results of all cohorts were combined and the s.e.-based weights were calculated using METAL^21^. The Bonferroni correction for multiple testing was 8.33 × 10^−3^. This resulted in the significant replication of five out of the six variants, see Supplementary Fig. 19 and Supplementary Table 11.

### Conditional analysis

Within the discovery cohorts we performed a conditional analysis to see whether the novel variants are independent of the known loci from refs 1, 2. Supplementary Table 10 shows the results within these cohorts with and without adjusting for the known loci for the trait in question, if available in the GoNL reference panel. Since the unadjusted and adjusted results are similar, we conclude that the newly identified variants are independent of the known loci.

## Author contributions

E.M.v.L. organized the study and designed the study with substantial input of L.C.K., A.I., P.I.W.d.B. and C.M.v.D. E.M.v.L. drafted the manuscript with substantial input of L.A.C., A.Me, B.M.P., C.W., G.M.P., J.F.W., J.E.H., L.C.F., L.C.K., J.D., P.E.S., D.I.B., J.E.H., H.M., P.M.K., P.I.W.d.B., S.L.P., S.T., C.M.v.D. and G.-J.B.v.O. All authors had the opportunity to comment on the manuscript. Data collection, GWAS and statistical analysis were performed by T.M.B., J.A.B., J.C.B., B.M.P. (CHS); J.E.H., C.H., O.P., V.V., I.R., A.F.W. (CROATIA); E.M.v.L., B.A.O., C.M.v.D. (ERF); C.C.W., L.A.C. (FHS), M.F.F., I.B.B. (FamHS); J.E.H., H.T., L.J.H., D.J.P. (Generation Scotland); G.M.P., Q.D., L.A.L. (JHS); A.Ma., J.I.R., J.C.M., S.S.R. (MESA); A.K., P.D., F.v.D., M.A.S., C.W. (Lifelines); J.D., M.B., A.J.M.C., H.-W.U., P.E.S. (LLS); H.M., G.W., E.J.d.G., Y.M., B.W.J.H.P., J.-J.H., D.I.B. (NTR-NESDA); N.V., I.M.L., P.v.H. (PREVEND); S.T., I.P., N.S., C.J.P., B.M.B., P.M.K., D.J.S., J.W.J. (PROSPER); P.K.J., H.C., J.F.W. (ORCADES); E.M.v.L., C.M.-G., F.R., A.H., O.H.F., E.J.S., A.G.U., C.M.v.D. (Rotterdam Study). D.J.v.E. recruited cohorts. Creation of the GoNL reference panel was carried out by L.C.F., A.Me., S.L.P. and P.D. Design of the GoNL project was made by C.W., M.A.S., C.M.v.D., D.I.B., P.E.S., G.-J.B.O., P.I.W.d.B. E.M.v.L. performed the meta-analysis. Biological association of loci and bioinformatics were carried out by E.M.v.L. and C.M.v.D.

## Additional information

**How to cite this article:** van Leeuwen, E. M. *et al* Genome of the Netherlands population-specific imputations identify a ABCA6 variant associated with cholesterol levels. *Nat. Commun.* 6:6065 doi: 10.1038/ncomms7065 (2015).

## Supplementary Material

Supplementary Information Supplementary Figures 1-19, Supplementary Tables 1-14, Supplementary Note 1, Supplementary Methods and Supplementary References

## Figures and Tables

**Table 1:** Summary descriptions for the variants associated with HDL-C, LDL-C, TC or TG.

**SNP**	**Chr**	**Position**	**EA**	**NEA**	**Gene**	**MAF**_**GoNL**_	**MAF**_**1-kG**_	**MAF**_**GoNL**_ **/MAF**_**1-kG**_ **(*P* value for two population proportions)**
rs4752801	11	47,907,641	G	A	Close to the *NUP160*	0.347	0.338	1.027 (0.258)
rs149580368	17	41,874,745	A	C	Between *C17orf105* and *MPP3*	0.029	0.015	1.923 (<0.0001)
rs77542162	17	67,081,278	G	A	* ABCA6 *	0.030	0.008	3.647 (<0.0001)
rs144984216	19	20,479,901	T	C	* ZNF826P *	0.028	0.011	2.555 (<0.0001)
rs117162033	19	8,627,569	T	C	* MYO1F *	0.007	0.007	0.957 (<0.0001)

EA, effect allele; GoNL, Genome of the Netherlands; HDL-C, high-density lipoprotein cholesterol; LDL-C, low-density lipoprotein cholesterol; MAF_GoNL_ and MAF_1_ _kG_, the minor allele frequency of the effect allele in the GoNL reference panel and in the 1-kG reference panel (Phase 1 integrated release v3, April 2012, all ancestries), respectively; NEA, non-effect allele; SNP, single-nucleotide polymorphism; TC, total cholesterol; TG, triglyceride.

**Table 2:** Results for the variants associated with HDL-C, LDL-C, TC or TG.

																	
