# Supplementary material for: Genome of the Netherlands population-specific imputations identify an ABCA6 variant associated with cholesterol levels
Source: Nat Commun. 2015 Mar 9;6:6065. doi: 10.1038/ncomms7065 (PMC4366498; doi:10.1038/ncomms7065)
Supplement: Supplementary Information — Supplementary Figures 1-19, Supplementary Tables 1-14, Supplementary Note 1, Supplementary Methods and Supplementary References [file ncomms7065-s1.pdf]

Supplementary figures

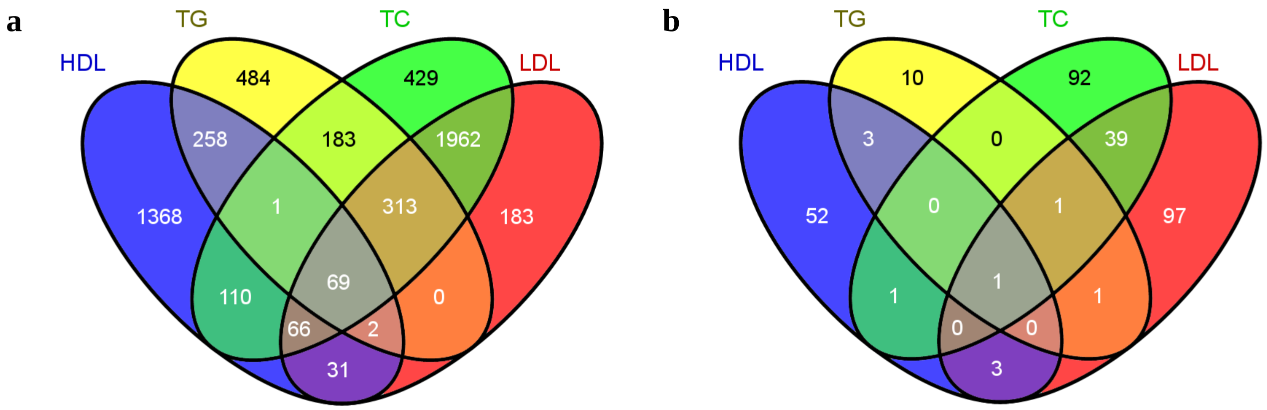

Supplementary Figure 1. The overlap between (a) the genome-wide significant (*p*-value $< 5 \cdot 10^{-8}$) SNPs per trait and (b) the independently associated SNPs per trait.

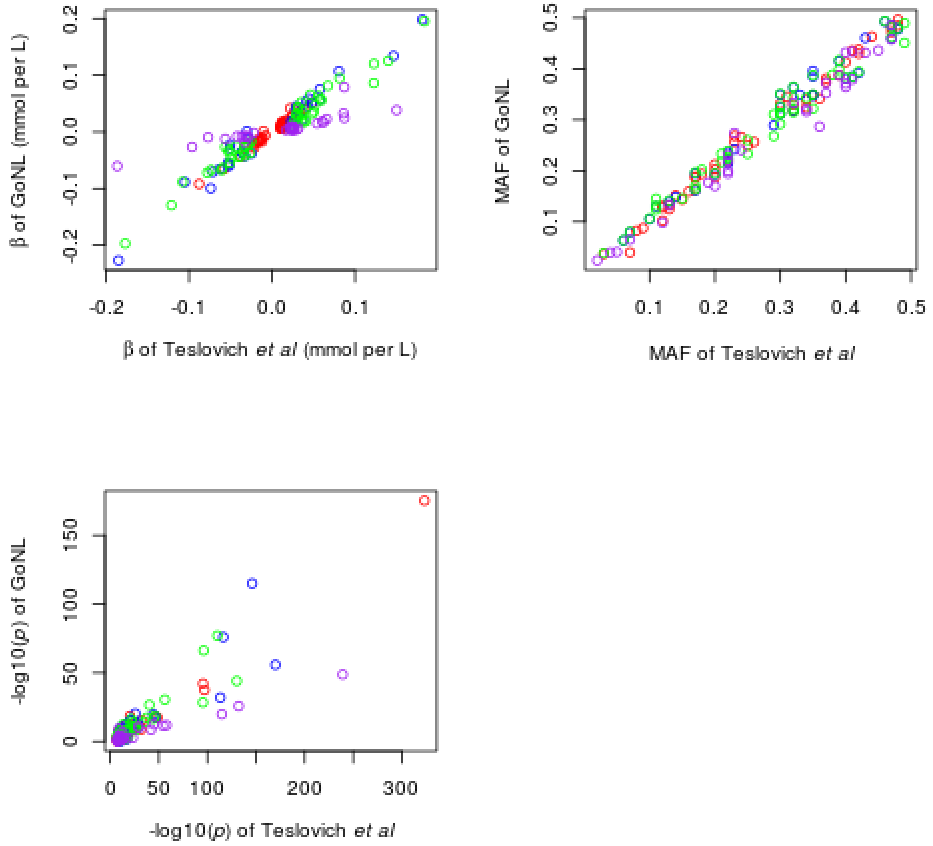

Supplementary Figure 2. The confirmation of the results of Teslovich *et al.*[1] in the meta-analysis of the discovery cohorts.

The red circles are the HDL loci, the blue circles are the LDL loci, the green circles are the TC loci and the purple circles are the TG loci.

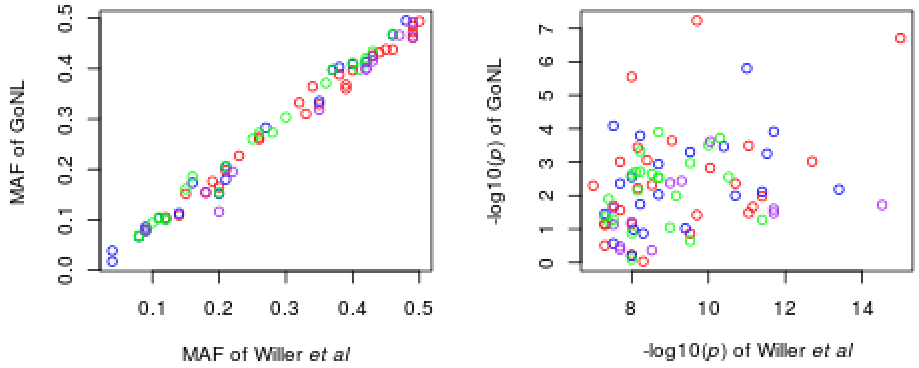

Supplementary Figure 3. The confirmation of the results of Willer *et al.*[2] in the meta-analysis of the discovery cohorts.

The red circles are the HDL loci, the blue circles are the LDL loci, the green circles are the TC loci and the purple circles are the TG loci.

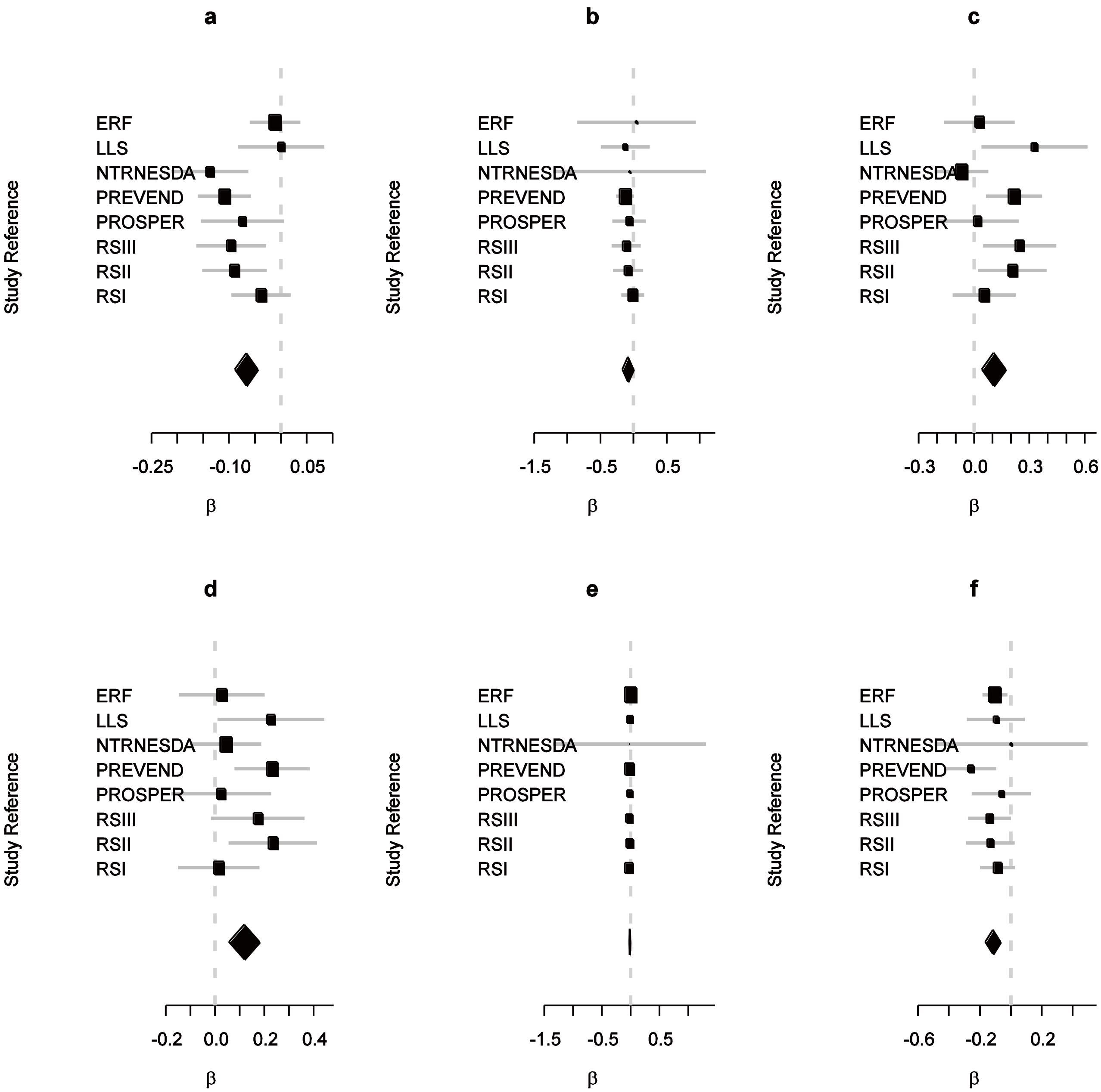

Supplementary Figure 4. The forestplot of all potential novel loci after the conditional analysis within the discovery cohorts.

a: HDL, chromosome 11, position 47,907,641.

b: HDL, chromosome 17, position 41,874,745.

c: LDL, chromosome 17, position 67,081,278.

d: TC, chromosome 17, position 67,081,278.

e: TC, chromosome 19, position 20,479,901.

f: TG, chromosome 19, position 8,627,569.

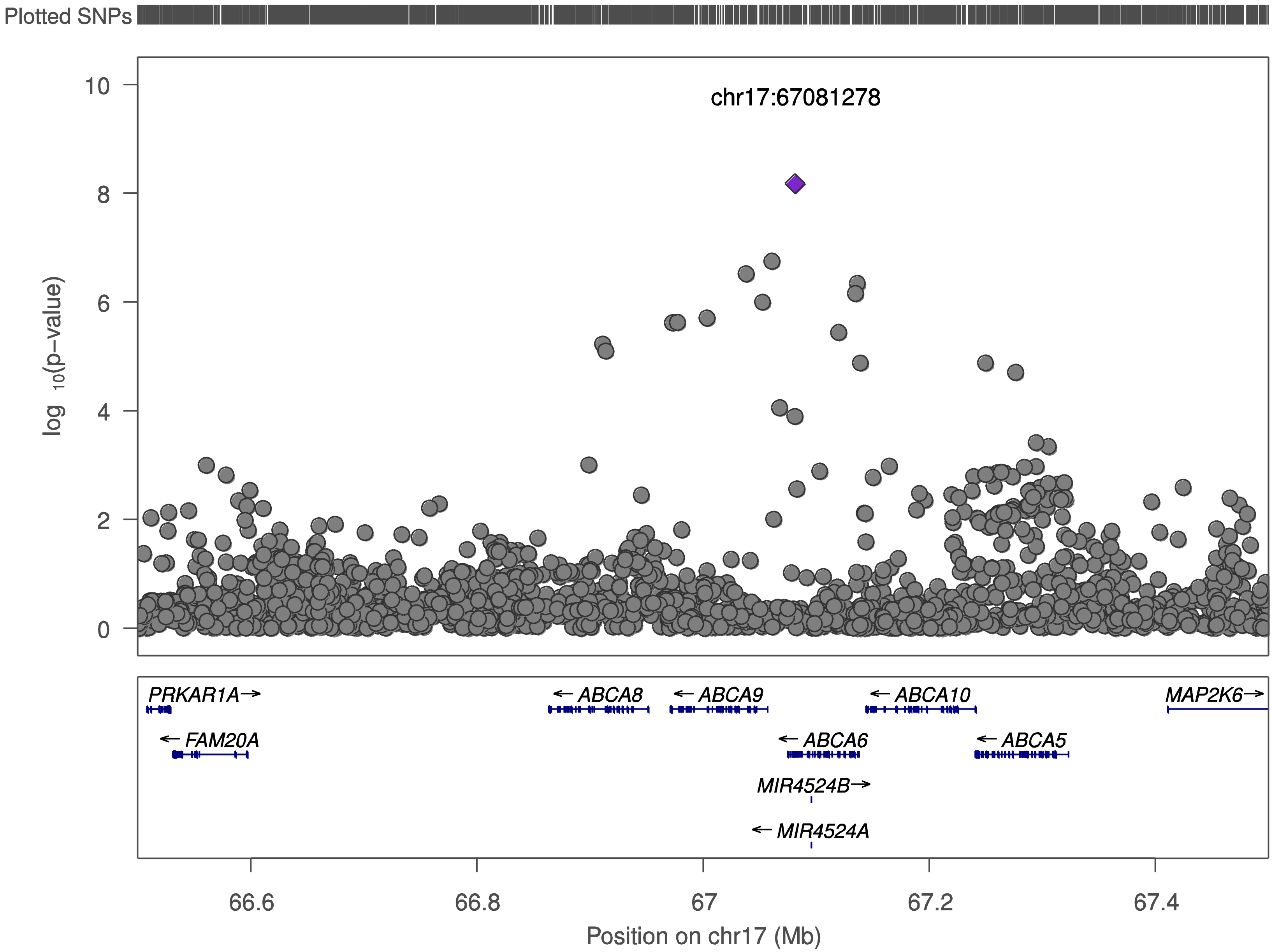

Supplementary Figure 5. The regional association results of the initial meta-analysis of all discovery cohorts for LDL on chromosome 17.

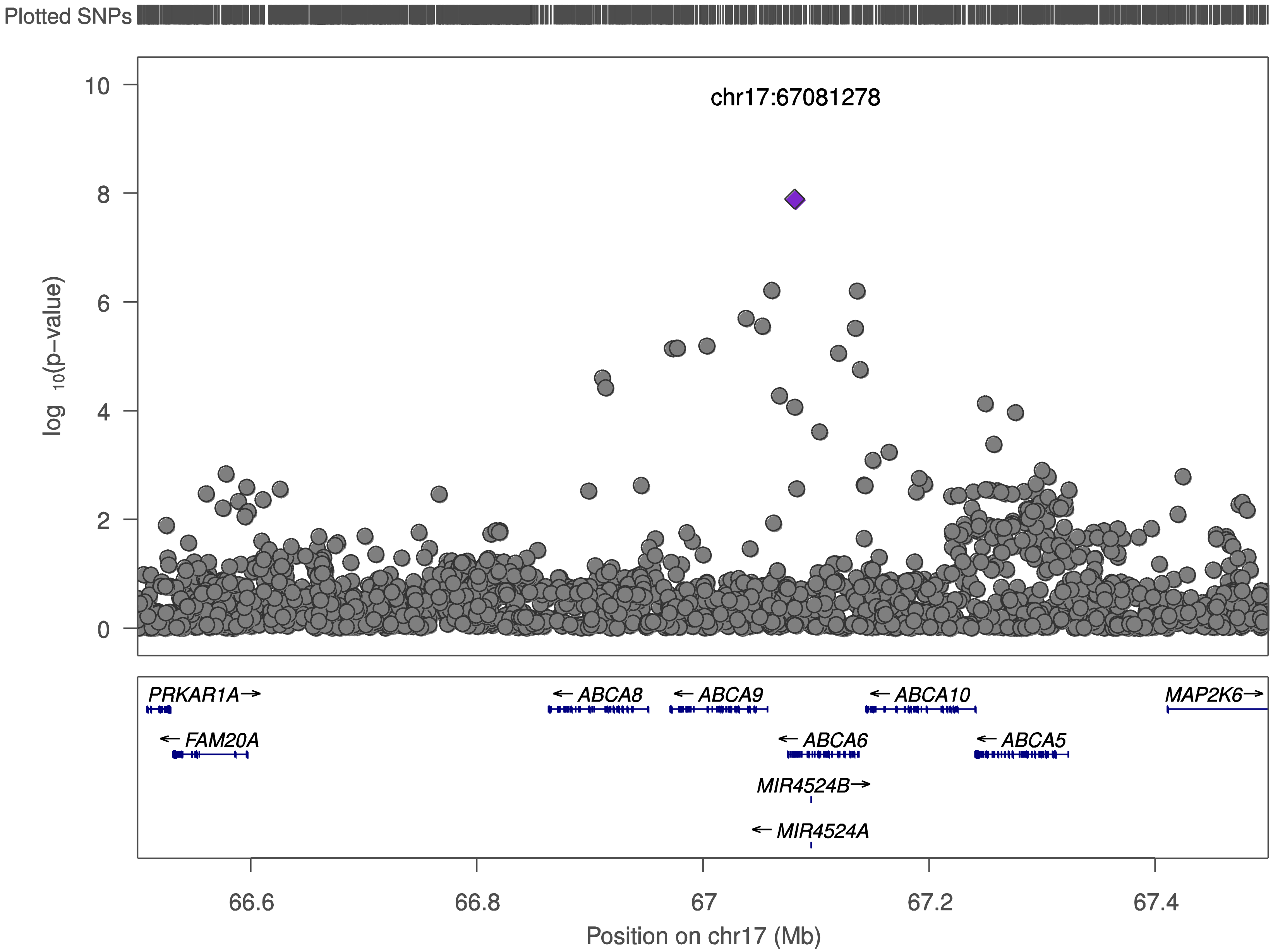

Supplementary Figure 6. The regional association results of the initial meta-analysis of all discovery cohorts for TC on chromosome 17.

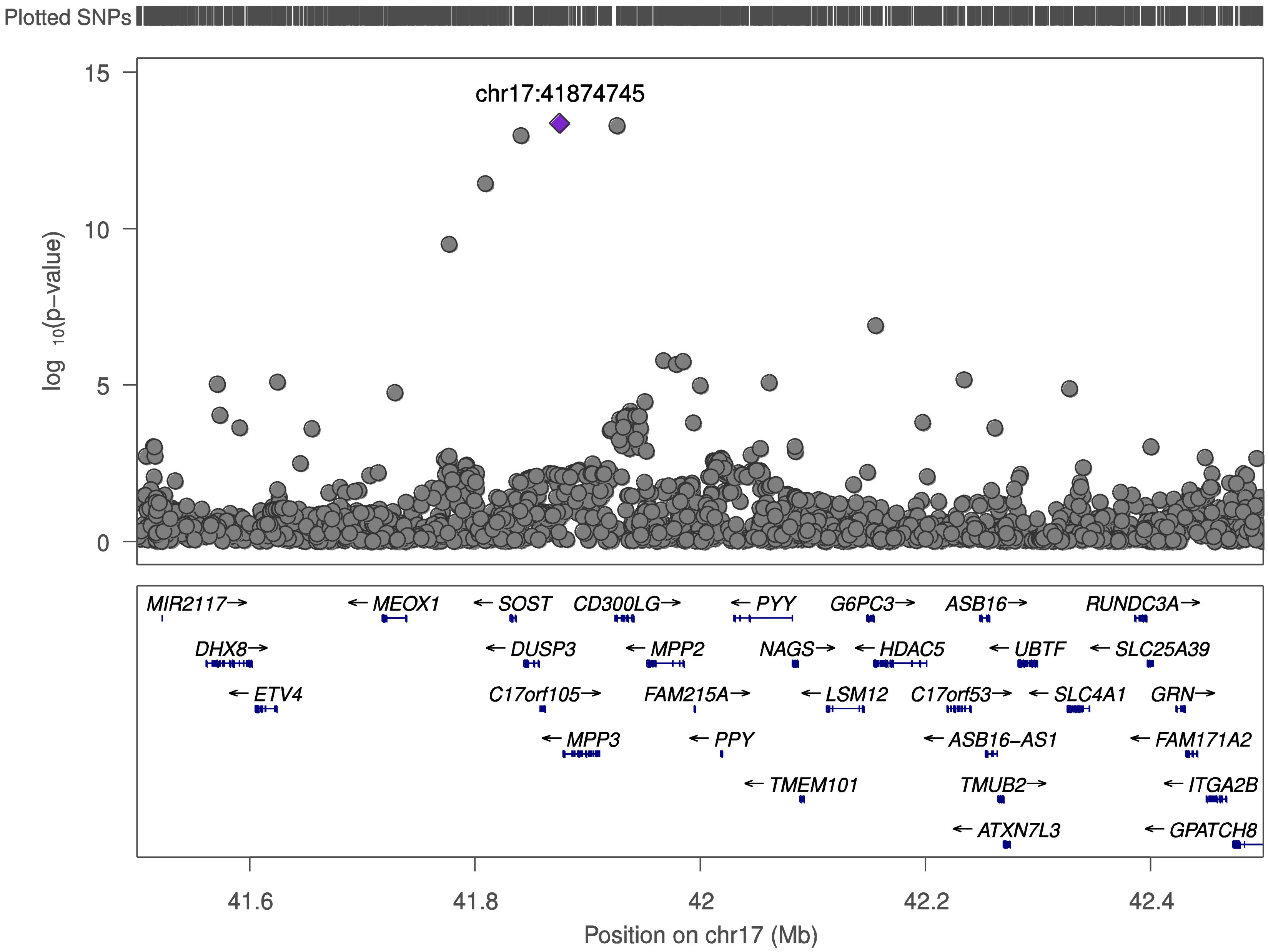

Supplementary Figure 7. The regional association results of the initial meta-analysis of all discovery cohorts for HDL on chromosome 17.

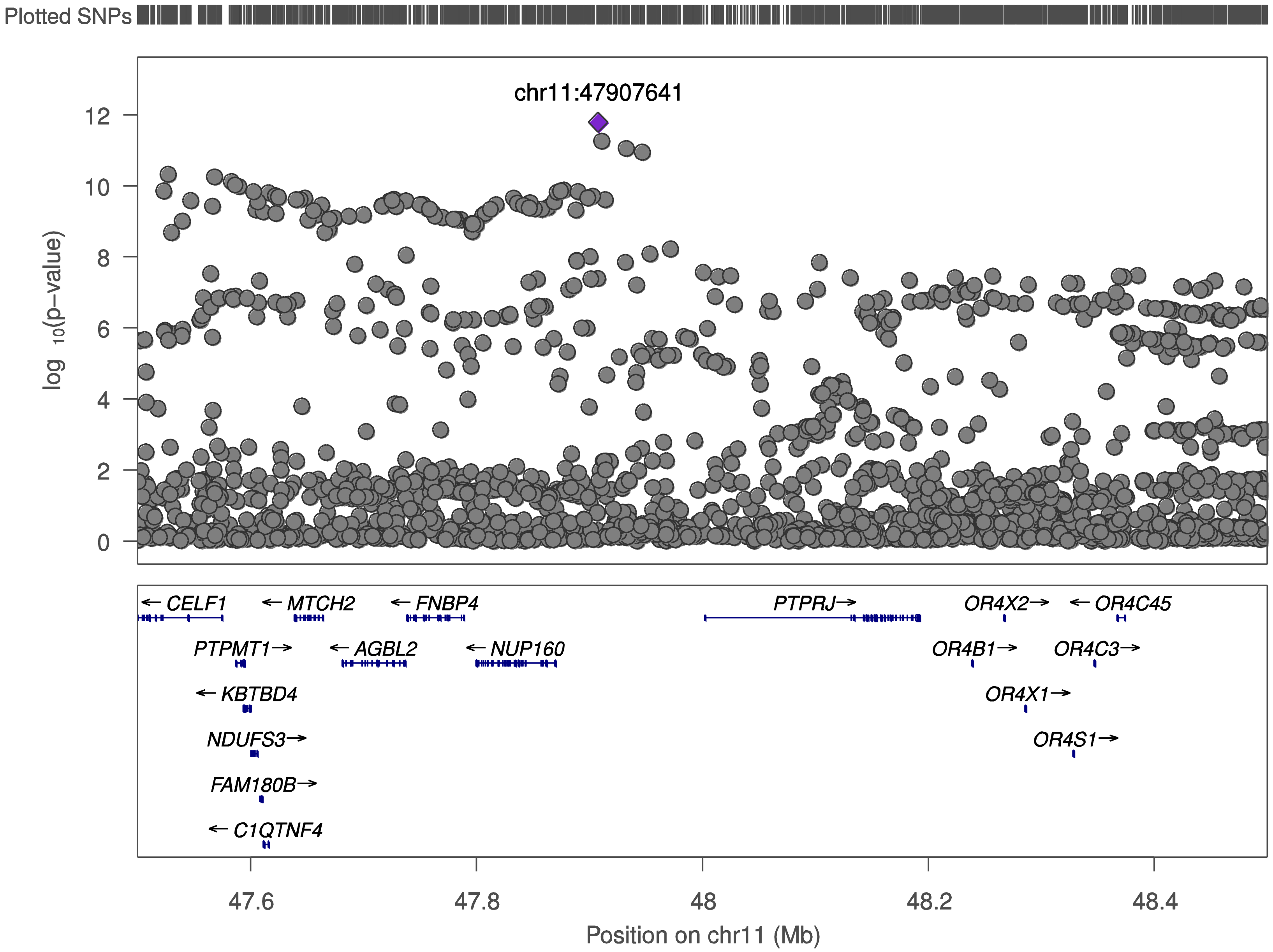

Supplementary Figure 8. The regional association results of the initial meta-analysis of all discovery cohorts for HDL on chromosome 11.

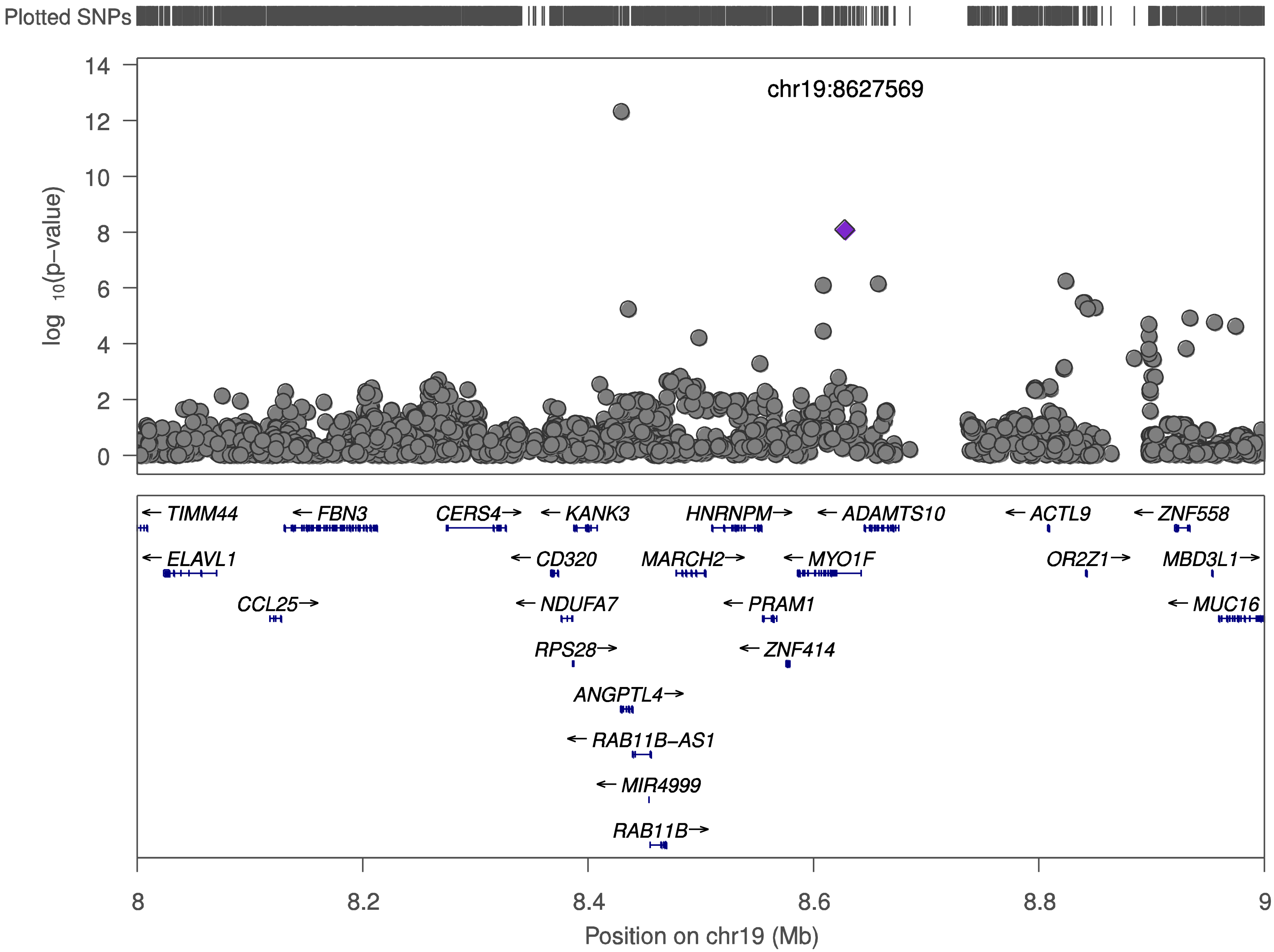

Supplementary Figure 9. The regional association results of the initial meta-analysis of all discovery cohorts for TG on chromosome 19.

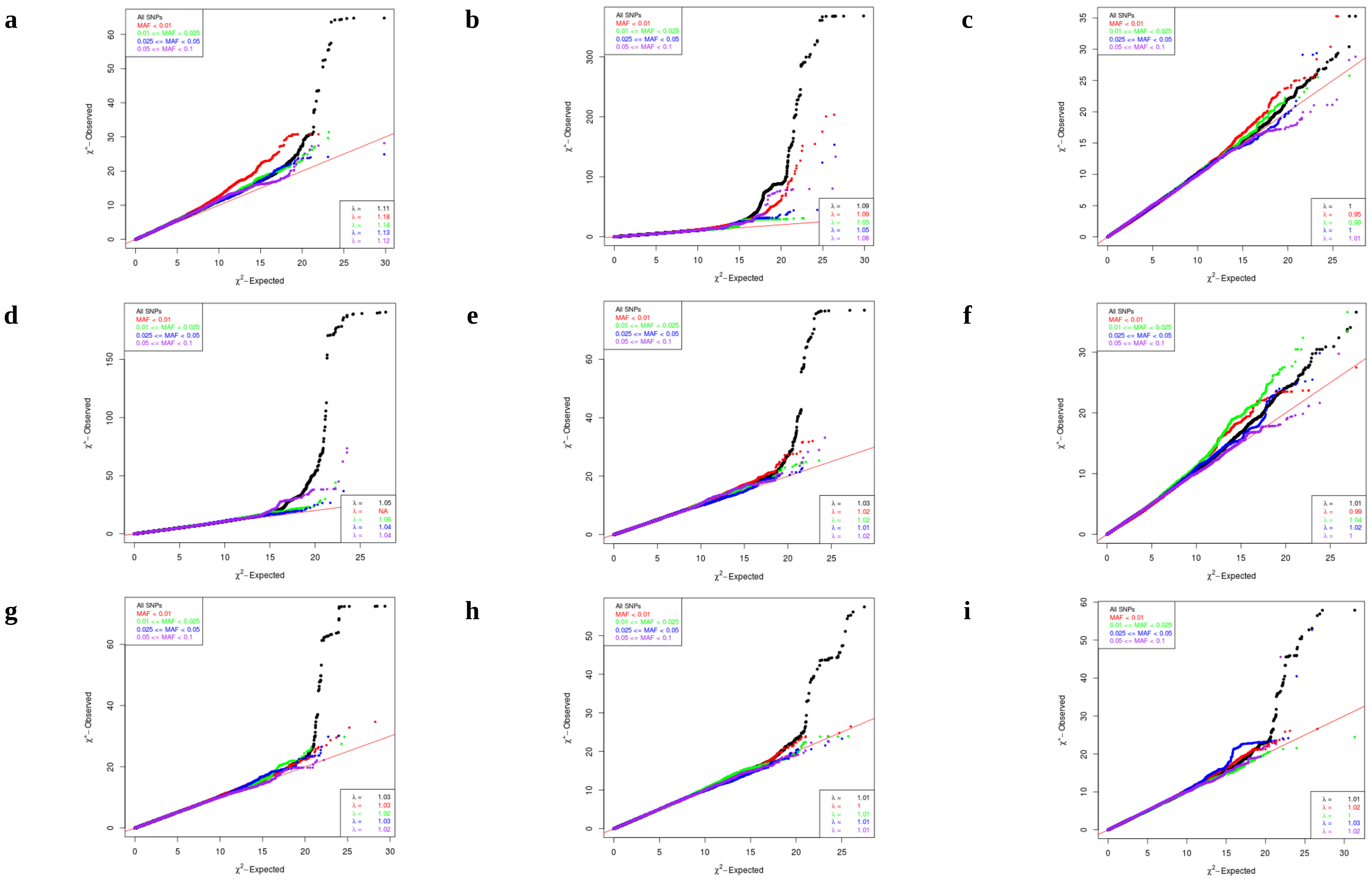

Supplementary Figure 10. The QQ plot for HDL per cohort after removing those SNPs which do not have association results or did not meet the QC thresholds.

a: ERF, b: Lifelines, c: LLS, d: NTR-NESDA, e: PREVEND, f: PROSPER, g: RS-I, h: RS-II and i: RS-III.

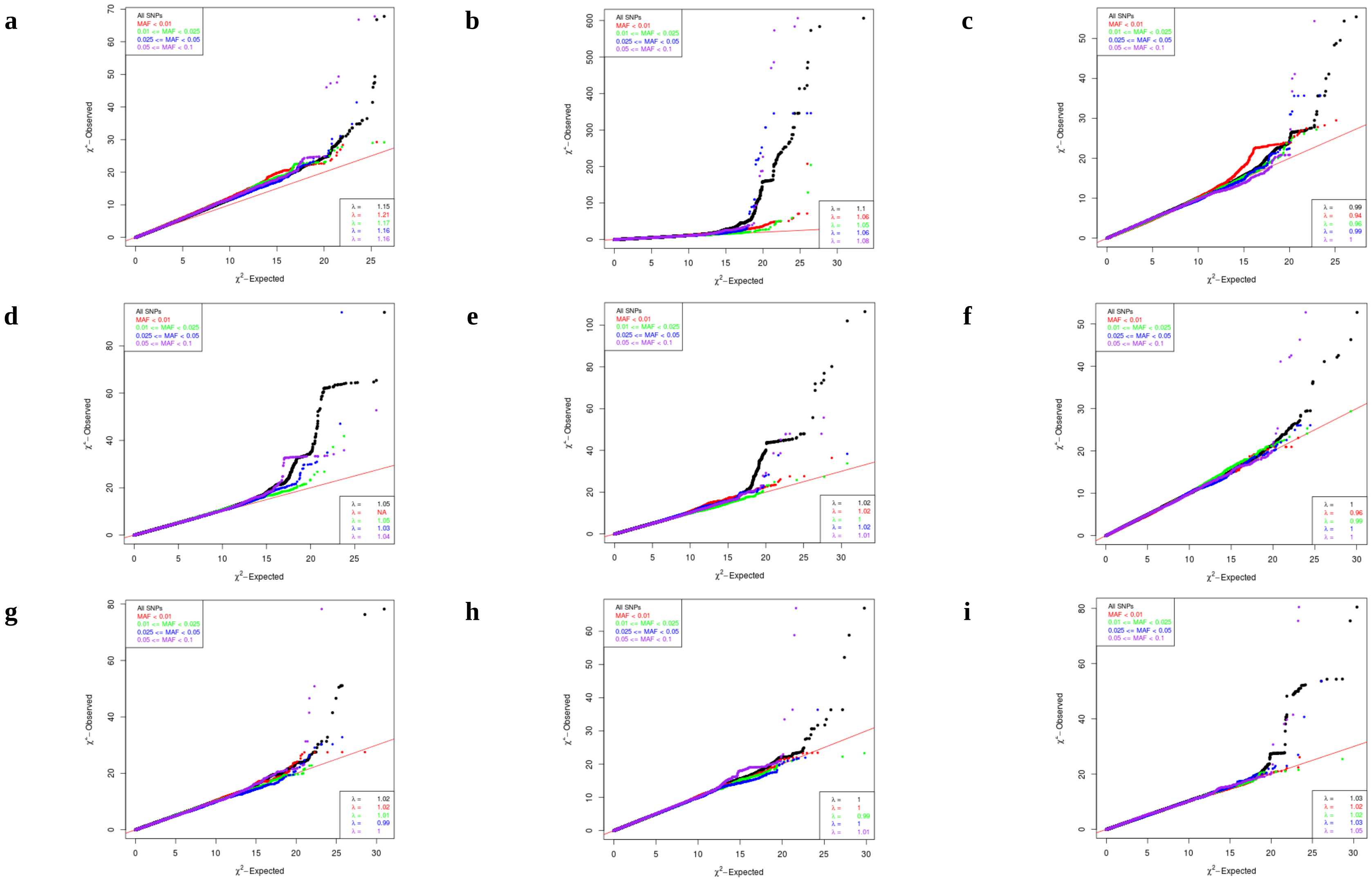

Supplementary Figure 11. The QQ plot for LDL per cohort after removing those SNPs which do not have association results or did not meet the QC thresholds.

a: ERF, b: Lifelines, c: LLS, d: NTR-NESDA, e: PREVEND, f: PROSPER, g: RS-I, h: RS-II and i: RS-III.

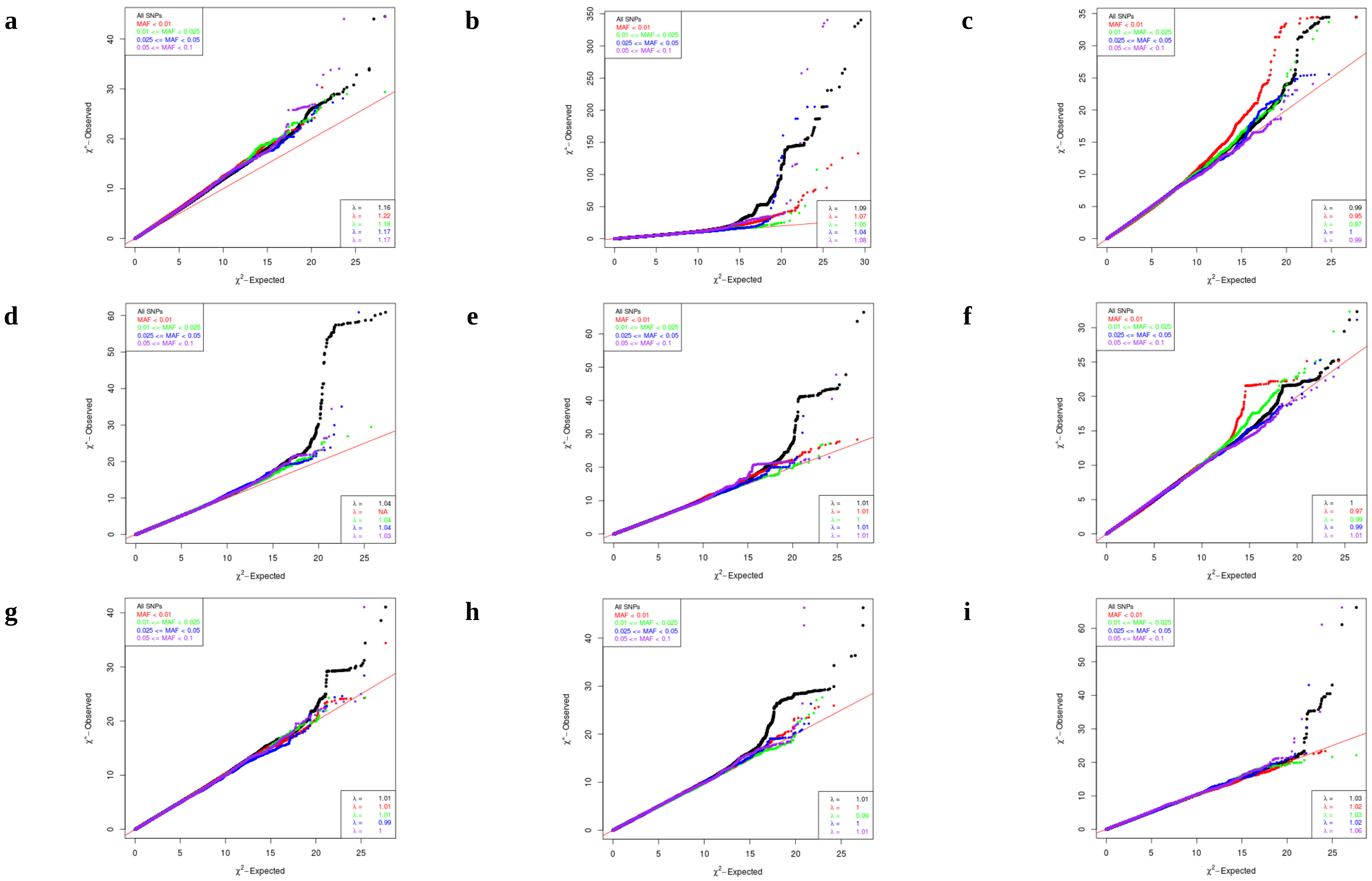

Supplementary Figure 12. The QQ plot for TC per cohort after removing those SNPs which do not have association results or did not meet the QC thresholds.

a: ERF, b: Lifelines, c: LLS, d: NTR-NESDA, e: PREVEND, f: PROSPER, g: RS-I, h: RS-II and i: RS-III.

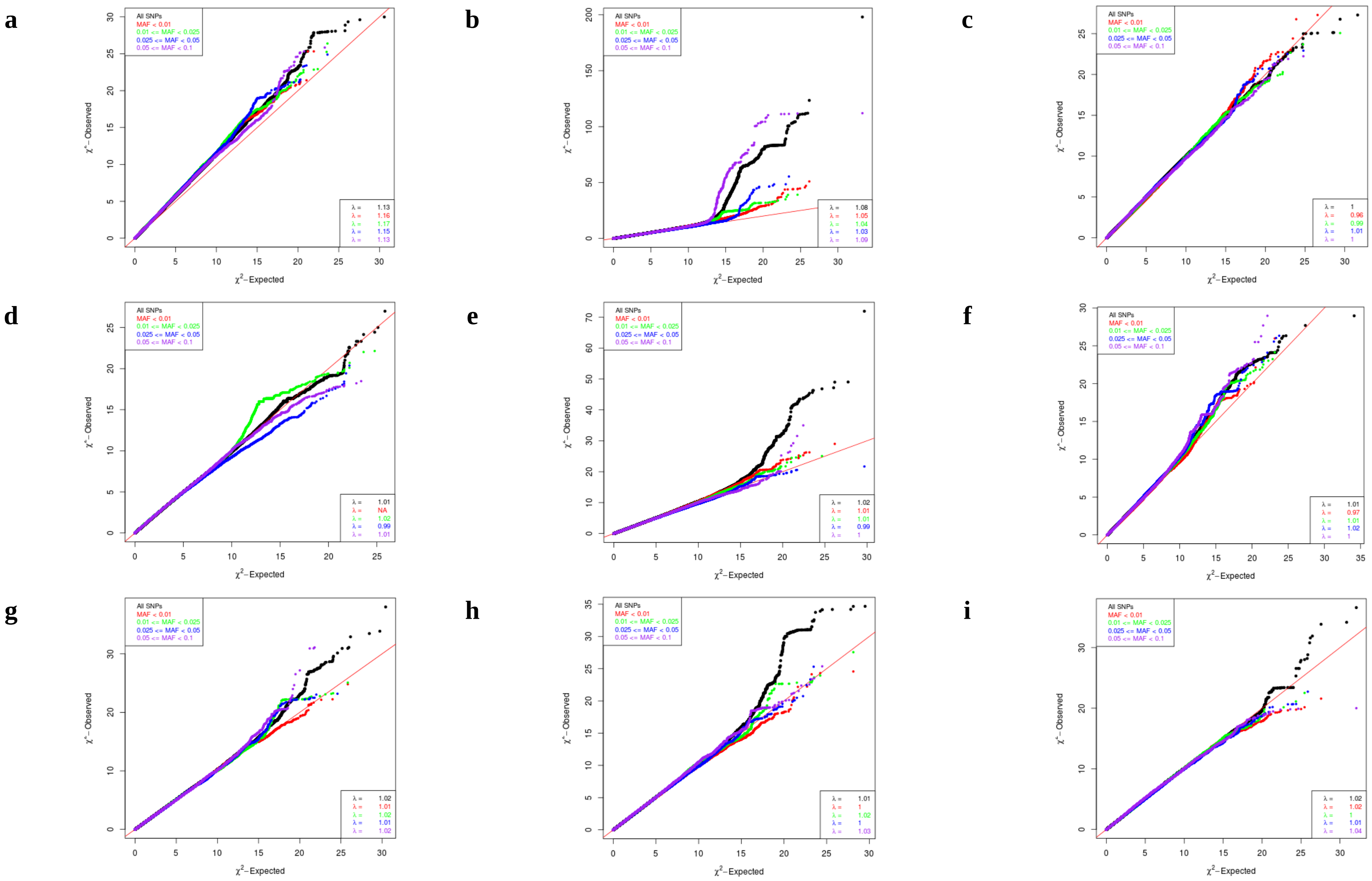

Supplementary Figure 13. The QQ plot for TG per cohort after removing those SNPs which do not have association results or did not meet the QC thresholds.

a: ERF, b: Lifelines, c: LLS, d: NTR-NESDA, e: PREVEND, f: PROSPER, g: RS-I, h: RS-II and i: RS-III.

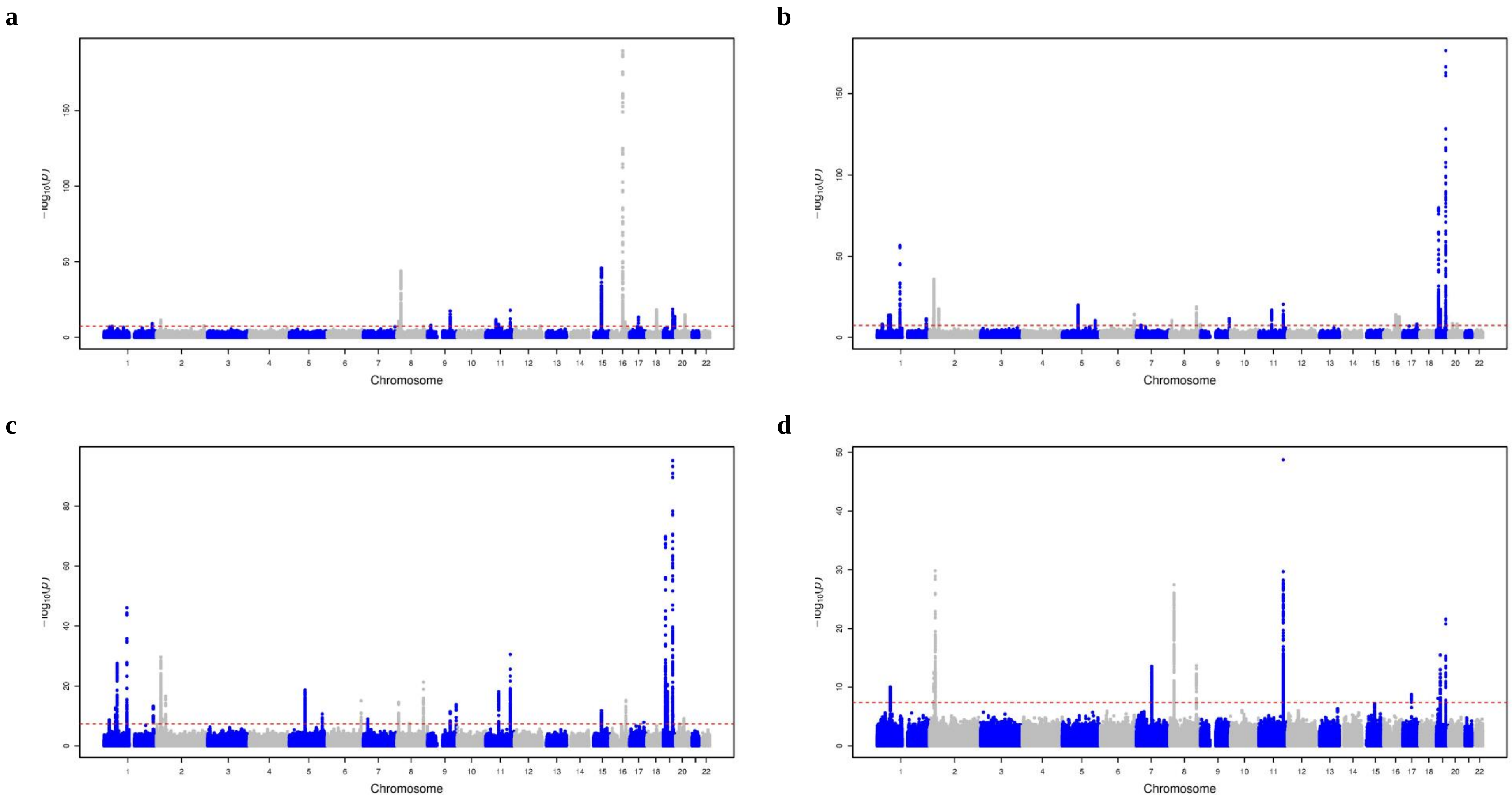

Supplementary Figure 14. The Manhattan plot of all the SNPs after meta-analysis per trait (a: HDL, b: LDL, c: TC and d: TG) and after filtering of the SNPs which do not have association results or did not meet the QC thresholds in less than 6 cohort of all discovery cohorts. Also, the SNPs that are labeled inaccessible by the Genome of the Netherlands project are removed to create the Manhattan plot.

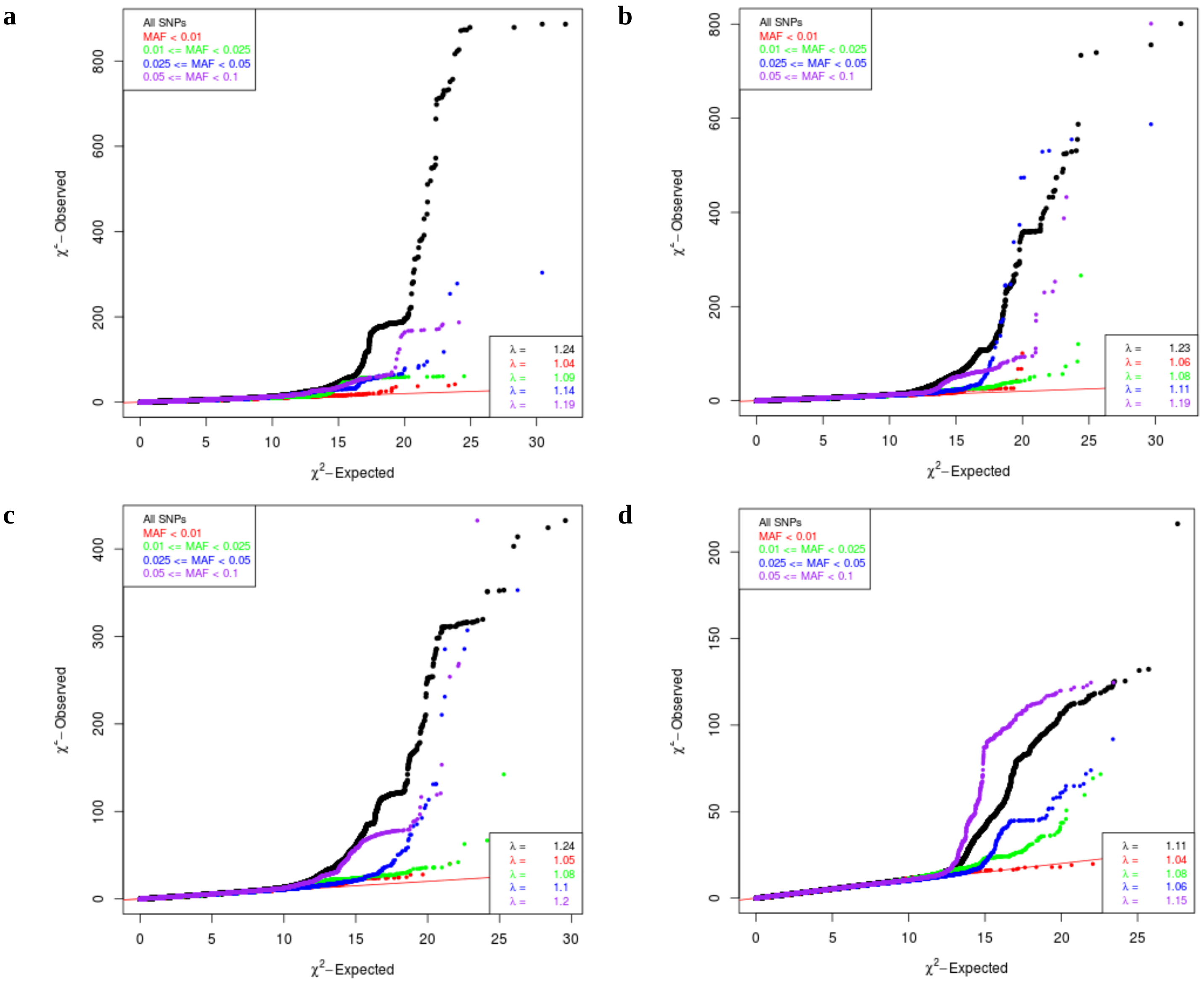

Supplementary Figure 15. The QQ plot of all the SNPs after meta-analysis per trait (a: HDL, b: LDL, c: TC and d: TG) and after filtering of the SNPs which do not have association results or did not meet the QC thresholds in less than 6 cohort of all discovery cohorts. Also, the SNPs that are labeled inaccessible by the Genome of the Netherlands project are removed to create the QQ plot.

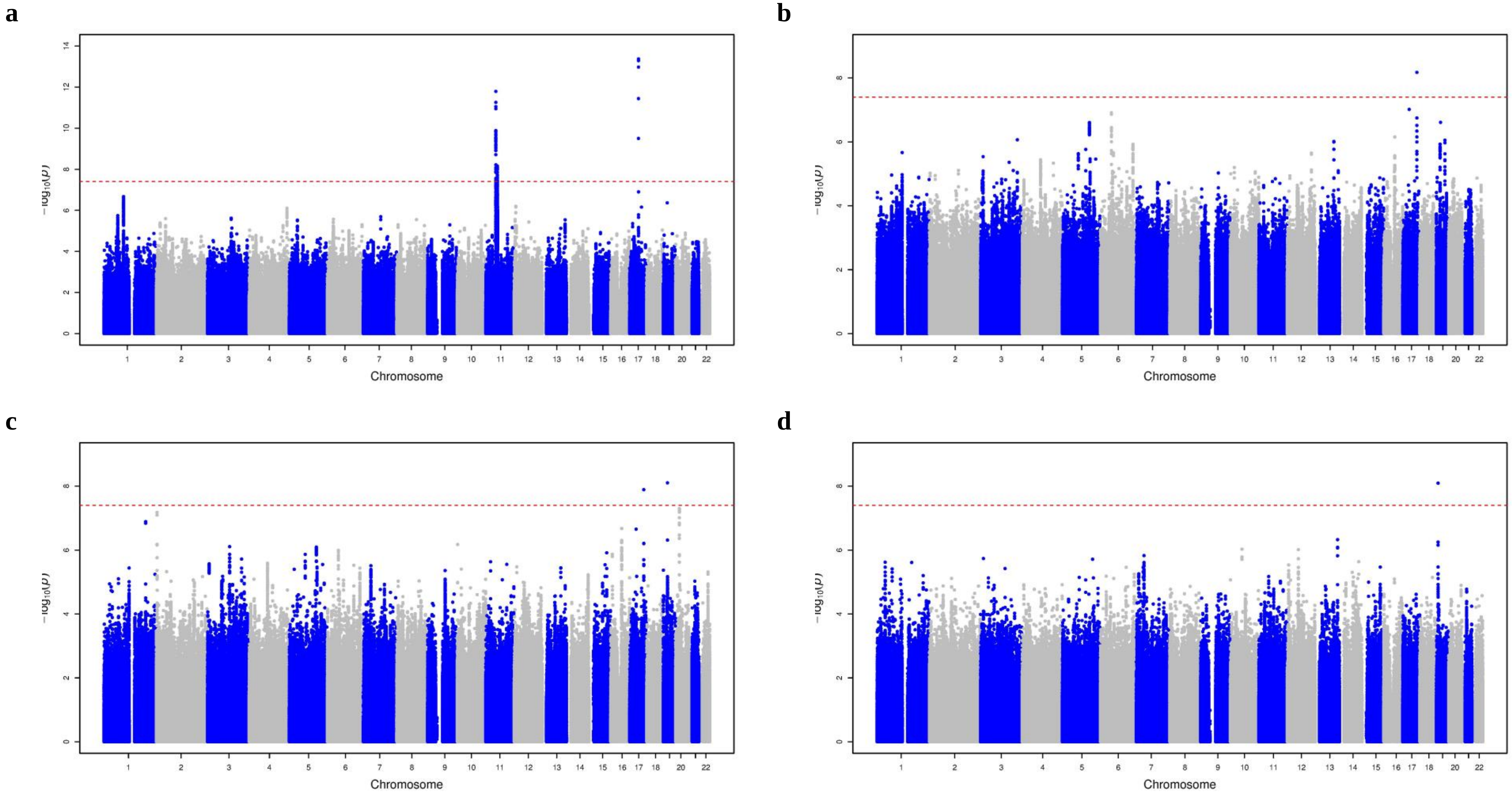

Supplementary Figure 16. The Manhattan plot of all the SNPs after exclusion of all SNPs within 1Mb of a known locus per trait.

a: HDL, b: LDL, c: TC and d: TG

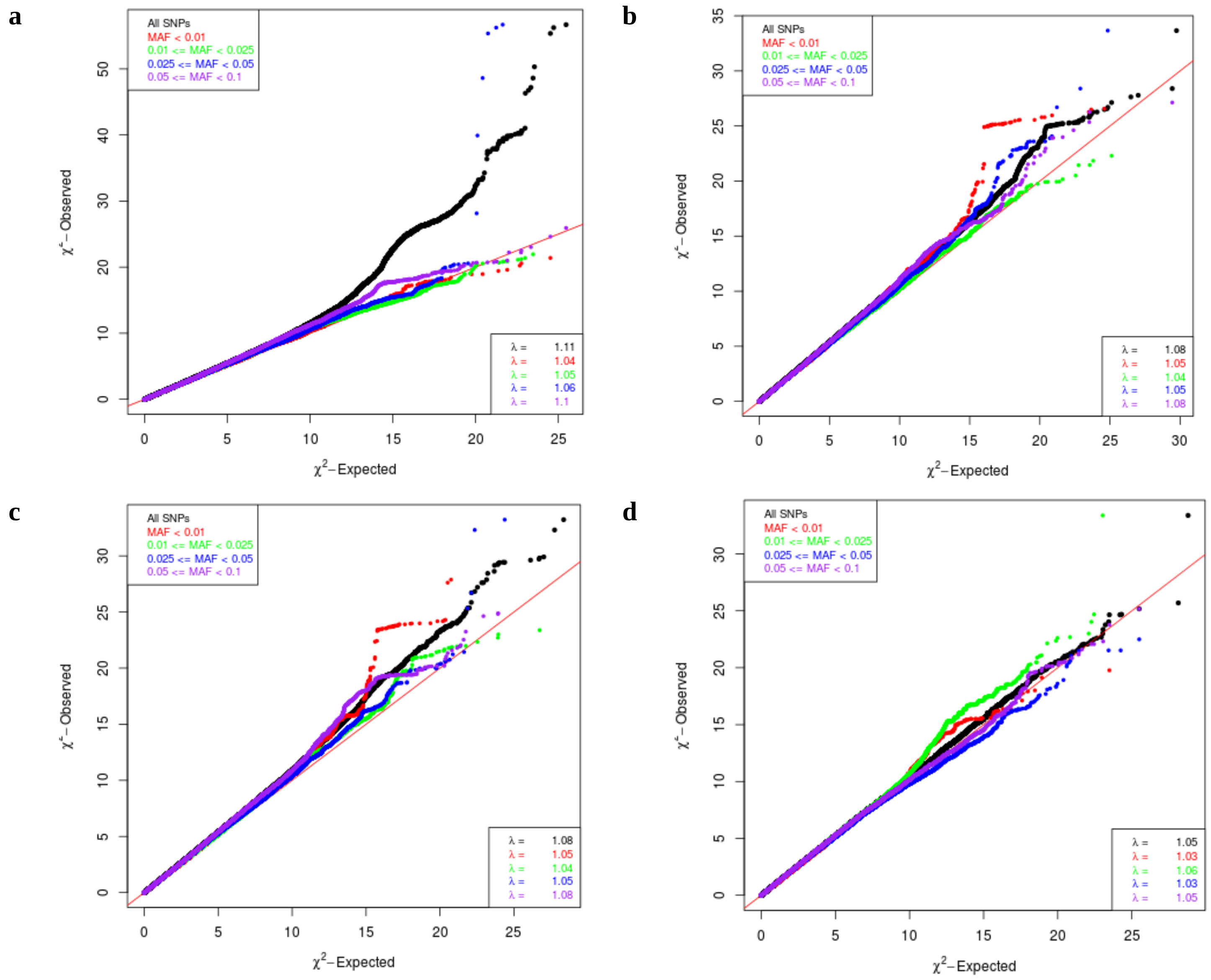

Supplementary Figure 17. The QQ plot of all the SNPs after exclusion of all SNPs within 1Mb of a known locus per trait.

a: HDL, b: LDL, c: TC and d: TG

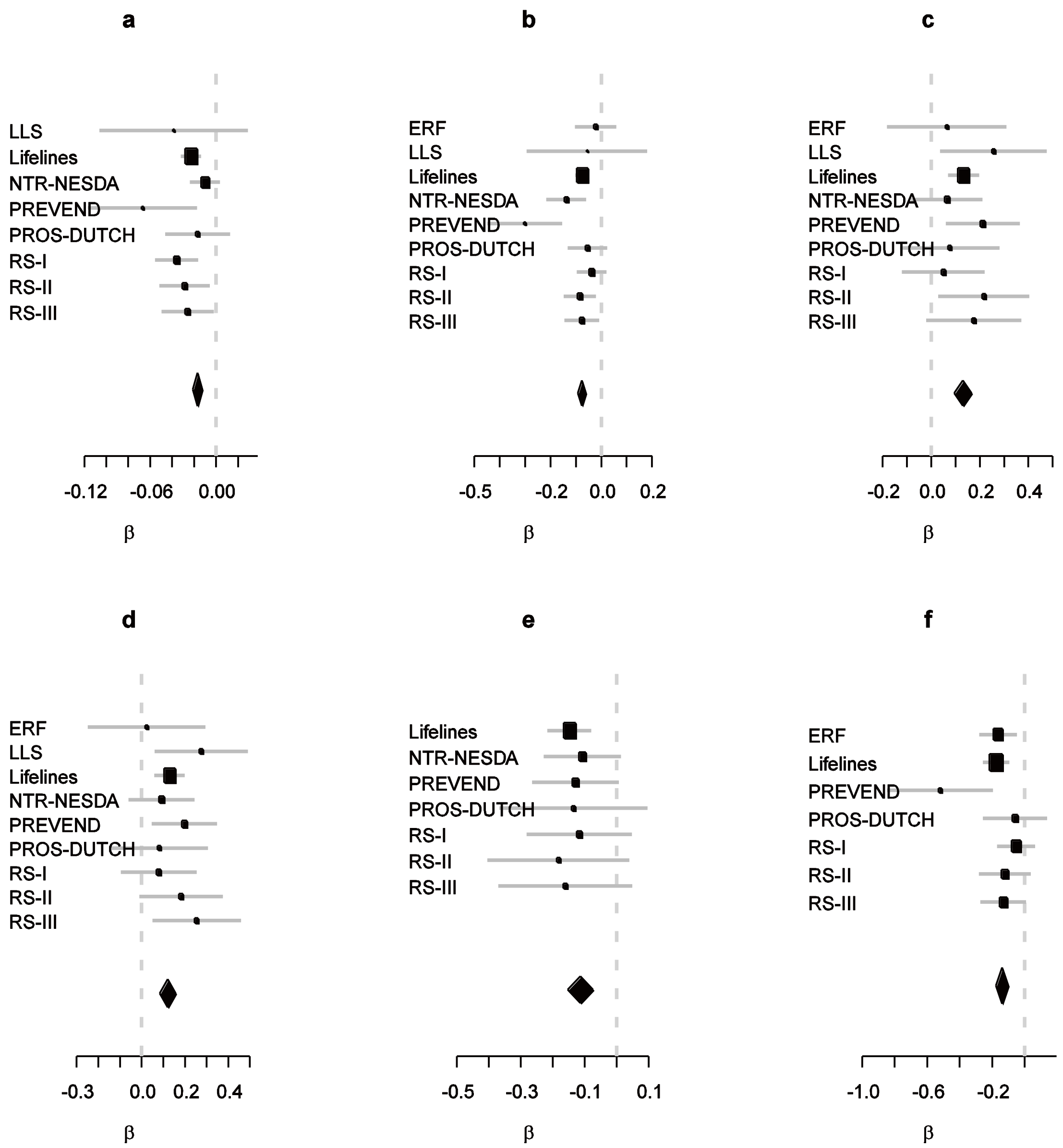

Supplementary Figure 18. The forestplot of all potential novel loci after the discovery phase.
Only cohorts of which the SNPs were not excluded after QC are included in the forestplot.

a: HDL, chromosome 11, position 47,907,641.

b: HDL, chromosome 17, position 41,874,745.

c: LDL, chromosome 17, position 67,081,278.

d: TC, chromosome 17, position 67,081,278.

e: TC, chromosome 19, position 20,479,901.

f: TG, chromosome 19, position 8,627,569.

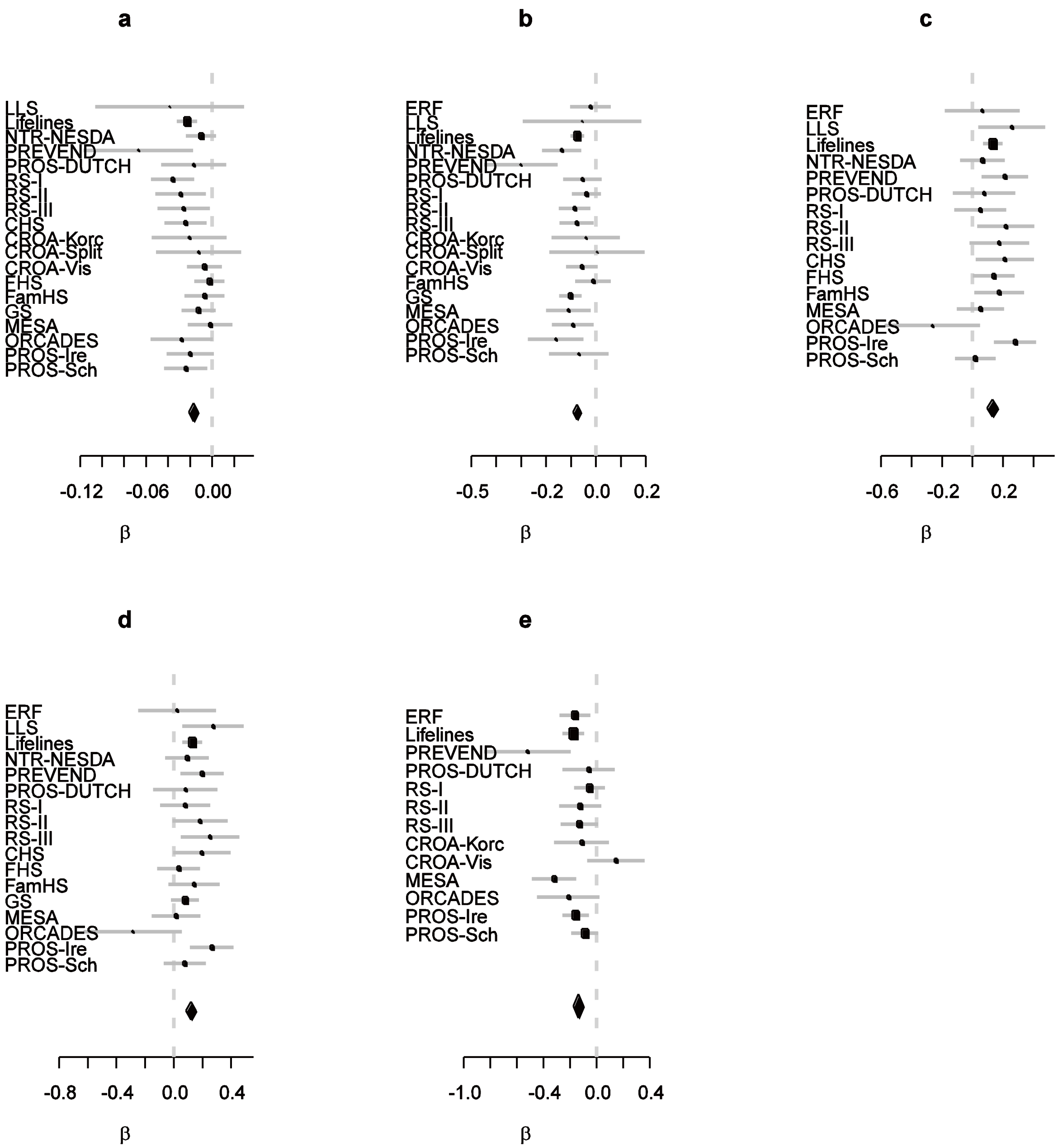

Supplementary Figure 19. The forestplot of all novel, significant replicated loci after combining the discovery and replication phase.

Only cohorts of which the SNPs were not excluded after QC are included in the forestplot.

a: HDL, chromosome 11, position 47,907,641.

b: HDL, chromosome 17, position 41,874,745.

c: LDL, chromosome 17, position 67,081,278.

d: TC, chromosome 17, position 67,081,278.

e: TG, chromosome 19, position 8,627,569.

Supplementary tables

Supplementary Table 1. Baseline characteristics for the discovery cohorts.

Cohort	HDL			LDL		
	N (% male)	Mean age (SD), years	Mean HDL-C (SD), in mmol L^{-1}	*N* (% male)	Mean age (SD), years	Mean LDL-C (SD), in mmol L^{-1}
ERF	2387 (43.57)	47.38 (14.29)	1.29 (0.36)	2376 (43.43)	47.34 (14.29)	3.81 (0.97)
Lifelines	12328 (40.72)	47.68 (11.03)	1.46 (0.39)	12331 (40.73)	47.68 (11.03)	3.34 (0.89)
LLS	2116 (44.99)	58.90 (6.85)	1.46 (0.45)	2054 (44.74)	58.92 (6.85)	3.38 (0.91)
NTR-NESDA	7564 (36.50)	42.41 (14.51)	1.47 (0.41)	7501 (36.20)	42.36 (14.53)	3.39 (1.01)
PREVEND	3434 (51.20)	49.26 (12.41)	1.31 (0.39)	3363 (50.80)	49.27 (12.45)	4.04 (1.09)
PROSPER	961 (52.30)	75.2 (3.3)	1.25 (0.32)	961 (52.30)	75.2 (3.3)	3.94 (0.78)
RS-I	2989 (57.10)	66.17 (7.22)	1.39 (0.39)	2884 (57.84)	66.16 (7.20)	4.17 (0.91)
RS-II	1859 (45.08)	64.67 (8.09)	1.38 (0.37)	1819 (44.91)	64.65 (8.10)	4.17 (0.91)
RS-III	2362 (41.79)	56.52 (6.69)	1.45 (0.43)	2335 (41.37)	56.52 (6.68)	3.95 (0.95)

Cohort	TC			TG		
	N (% male)	Mean age (SD), years	Mean TC-C (SD), in mmol L^{-1}	*N* (% male)	Mean age (SD), years	Mean TG-C (SD), in mmol L^{-1}
ERF	2388 (43.61)	47.38 (14.29)	5.65 (1.08)	2357 (43.23)	47.30 (14.31)	1.28 (0.65)
Lifelines	12331 (40.73)	47.69 (11.03)	5.16 (0.99)	12220 (40.37)	47.69 (11.04)	1.20 (0.65)
LLS	2099 (45.07)	58.90 (6.84)	5.66 (1.10)	-	-	-
NTR-NESDA	7580 (36.50)	42.4 (14.51)	5.12 (1.07)	7530 (36.20)	42.37 (14.52)	1.28 (0.67)
PREVEND	3488 (51.20)	49.25 (12.42)	5.65 (1.09)	3436 (51.20)	49.26 (12.41)	1.43 (1.00)
PROSPER	961 (52.30)	75.2 (3.3)	5.78 (0.87)	961 (52.30)	75.2 (3.3)	1.48 (0.67)
RS-I	3039 (57.82)	66.22 (7.22)	5.87 (0.97)	2981 (58.37)	66.45 (7.43)	1.47 (0.64)
RS-II	1858 (45.05)	64.66 (8.08)	5.86 (0.95)	1828 (44.97)	64.65 (8.10)	1.51 (0.70)
RS-III	2365 (41.78)	56.51 (6.65)	5.70 (1.01)	2339 (41.30)	56.54 (6.71)	1.41 (0.67)

Supplementary Table 2. Independent variants associated with HDL after discovery phase.
EA: effect allele. NEA: non-effect allele. MAF and MAF_{GoNL}: the minor allele frequency of the effect allele in the discovery cohorts and in the GoNL reference panel. *N*: estimated effective sample size, determined by GCTA.

Trait	Chr	Position	EA	NEA	MAF	After METAL analysis			*N*	MAF_{GoNL}	After GCTA analysis		
						β	$SE_β$	*p*-value			β	$SE_β$	*p*-value
HDL	1	40,035,928	T	G	0.214	-0.020	0.004	5.08E-08	36625.8	0.215	-0.020	0.004	4.84E-08
HDL	1	230,296,153	C	T	0.402	-0.020	0.003	6.61E-10	34235.8	0.424	-0.020	0.003	5.12E-10
HDL	2	21,226,560	A	G	0.218	0.025	0.004	3.19E-12	39941.1	0.223	0.025	0.004	1.75E-12
HDL	2	227,125,254	G	A	0.379	0.017	0.003	2.00E-08	39442	0.363	0.017	0.003	1.21E-08
HDL	8	9,181,395	A	G	0.093	-0.035	0.005	2.91E-11	36742.7	0.092	-0.035	0.005	2.57E-11
HDL	8	19,772,641	C	T	0.019	-0.090	0.012	3.83E-14	32890.6	0.018	-0.078	0.012	4.25E-11
HDL	8	19,793,581	T	C	0.027	-0.096	0.010	3.21E-20	28968.8	0.028	-0.103	0.010	7.61E-23
HDL	8	19,819,328	C	T	0.106	0.067	0.005	1.27E-44	38222.9	0.117	0.042	0.006	1.40E-13
HDL	8	19,819,439	G	A	0.295	0.044	0.003	1.75E-41	36775.2	0.296	0.028	0.004	1.16E-12
HDL	8	126,495,818	T	C	0.463	0.017	0.003	4.40E-08	37391.8	0.453	0.017	0.003	3.18E-08
HDL	9	15,298,666	T	C	0.164	-0.023	0.004	8.10E-09	38493.9	0.145	-0.023	0.004	7.81E-09
HDL	9	107,661,742	C	A	0.263	-0.030	0.003	3.29E-18	37633.1	0.246	-0.032	0.003	1.35E-20
HDL	9	107,690,124	G	A	0.333	0.015	0.003	1.21E-06	39508.4	0.324	0.018	0.003	3.74E-09
HDL	11	47,676,170	A	G	0.497	0.001	0.003	8.01E-01	37237.3	0.495	0.025	0.004	2.05E-10
HDL	11	47,907,641	G	A	0.355	-0.023	0.003	1.62E-12	35668.4	0.347	-0.040	0.004	1.74E-21
HDL	11	61,603,510	A	C	0.325	-0.020	0.003	2.17E-09	35034.9	0.355	-0.020	0.003	3.50E-09
HDL	11	116,648,917	G	C	0.133	-0.041	0.005	1.00E-18	34302.5	0.133	-0.058	0.005	6.02E-30
HDL	11	116,705,516	G	A	0.282	0.011	0.003	1.62E-03	35784.6	0.289	0.030	0.004	5.61E-15
HDL	12	123,790,007	C	T	0.085	0.029	0.005	2.98E-08	38558.3	0.076	0.030	0.005	9.88E-09
HDL	12	125,259,888	A	G	0.340	0.018	0.003	2.24E-08	36584.4	0.352	0.019	0.003	7.57E-09
HDL	15	58,571,910	T	C	0.279	-0.020	0.003	1.32E-09	38733.1	0.281	-0.022	0.003	2.20E-11
HDL	15	58,671,765	G	T	0.352	0.043	0.003	6.14E-41	36133.9	0.333	0.024	0.004	2.20E-08
HDL	15	58,680,178	C	T	0.360	0.045	0.003	1.30E-46	38117	0.328	0.028	0.004	7.07E-11
HDL	15	58,730,498	C	T	0.211	0.040	0.004	2.36E-27	37165.2	0.202	0.043	0.004	1.39E-30
HDL	16	49,498,861	C	G	0.245	-0.006	0.004	1.22E-01	30589.1	0.231	-0.023	0.004	6.41E-09
HDL	16	51,493,551	G	C	0.078	-0.011	0.006	5.33E-02	39534.8	0.077	-0.047	0.006	5.96E-17
HDL	16	51,980,822	G	A	0.030	0.007	0.009	4.31E-01	36183.4	0.033	0.062	0.009	1.58E-11
HDL	16	54,316,920	C	G	0.211	-0.007	0.004	1.18E-01	29352.1	0.193	-0.047	0.004	6.10E-27
HDL	16	54,655,478	G	C	0.022	0.030	0.012	1.09E-02	29737.8	0.026	0.125	0.012	2.69E-25
HDL	16	55,733,248	A	G	0.122	-0.021	0.007	3.13E-03	16421.4	0.113	-0.054	0.007	3.37E-14
HDL	16	56,576,250	T	G	0.012	-0.020	0.016	2.29E-01	27794.4	0.017	0.104	0.017	7.20E-10
HDL	16	56,624,079	G	A	0.421	0.004	0.003	2.57E-01	34492.2	0.405	-0.025	0.003	2.04E-13
HDL	16	56,759,695	A	G	0.036	0.032	0.008	1.16E-04	36296.7	0.042	0.115	0.009	7.61E-39
HDL	16	56,933,409	A	G	0.044	0.015	0.008	5.43E-02	35363.7	0.039	0.141	0.008	4.84E-66
HDL	16	56,982,299	T	C	0.373	0.009	0.003	5.62E-03	38320.8	0.353	0.028	0.003	1.47E-16
HDL	16	56,985,514	A	G	0.334	0.094	0.003	1.99E-174	34727.6	0.328	0.123	0.006	2.65E-87
HDL	16	56,990,803	G	A	0.247	-0.039	0.004	5.26E-28	35627.5	0.246	-0.240	0.008	4.88E-205
HDL	16	56,991,363	T	C	0.334	0.092	0.003	4.13E-190	39381.6	0.329	0.197	0.007	1.65E-152
HDL	16	56,993,897	C	A	0.212	-0.027	0.004	3.01E-11	32173.2	0.180	-0.217	0.007	2.17E-194
HDL	16	56,994,212	C	T	0.269	-0.057	0.003	6.27E-62	37604.8	0.280	0.090	0.006	4.48E-47
HDL	16	56,994,894	A	G	0.499	0.001	0.003	8.33E-01	35844.1	0.533	-0.411	0.008	0.00E+00
HDL	16	57,033,607	A	G	0.210	0.008	0.004	4.91E-02	32393.4	0.213	-0.044	0.004	2.53E-25
HDL	16	57,170,189	C	T	0.042	0.019	0.008	1.27E-02	38481.3	0.050	0.129	0.008	1.73E-60
HDL	16	57,470,884	C	A	0.150	0.006	0.004	1.68E-01	38287	0.155	0.037	0.004	7.78E-18
HDL	16	57,911,524	T	C	0.011	-0.041	0.021	5.02E-02	18791	0.015	-0.160	0.022	1.22E-13
HDL	16	63,121,015	A	G	0.086	-0.008	0.005	1.21E-01	39123.1	0.090	-0.044	0.005	3.82E-16

HDL	16	64,729,743	A	G	0.161	0.005	0.004	2.31E-01	34408.7	0.161	0.031	0.004	2.63E-12
HDL	16	66,514,962	T	C	0.059	-0.001	0.007	8.85E-01	36480.7	0.064	-0.046	0.007	4.10E-12
HDL	16	66,687,201	T	C	0.030	0.045	0.011	2.72E-05	25830	0.025	0.085	0.011	1.10E-14
HDL	16	67,655,133	C	T	0.128	0.028	0.005	6.55E-09	33452.4	0.125	0.028	0.005	1.59E-08
HDL	17	37,829,571	C	A	0.389	-0.018	0.003	5.63E-08	33396.7	0.416	-0.019	0.003	4.31E-09
HDL	17	41,874,745	A	C	0.036	-0.075	0.010	4.23E-14	25141.2	0.029	-0.078	0.010	5.72E-15
HDL	18	47,096,294	A	G	0.174	-0.022	0.004	5.52E-08	34858	0.174	-0.026	0.004	2.00E-10
HDL	18	47,158,186	T	C	0.166	-0.035	0.004	5.17E-19	38116	0.168	-0.038	0.004	4.74E-21
HDL	18	47,169,340	G	T	0.013	0.111	0.015	4.49E-14	30630.7	0.011	0.107	0.015	4.01E-13
HDL	19	45,422,160	G	C	0.187	-0.039	0.004	2.36E-19	31772.3	0.200	-0.044	0.004	1.92E-24
HDL	19	45,448,465	G	T	0.345	0.018	0.003	1.03E-07	34267.7	0.347	0.022	0.003	2.67E-10
HDL	19	54,809,343	G	T	0.195	0.033	0.004	2.03E-14	30755.7	0.195	0.036	0.004	9.67E-17
HDL	20	43,042,364	T	C	0.037	-0.052	0.009	8.65E-09	29736.3	0.037	-0.058	0.009	1.41E-10
HDL	20	44,545,460	G	A	0.268	-0.027	0.003	9.87E-16	39733.7	0.271	-0.028	0.003	1.34E-17

Supplementary Table 3. Independent variants associated with LDL after discovery phase.
EA: effect allele. NEA: non-effect allele. MAF and MAF_{GoNL}: the minor allele frequency of the effect allele in the discovery cohorts and in the GoNL reference panel. *N*: estimated effective sample size, determined by GCTA.

Trait	Chr	Position	EA	NEA	MAF	After METAL analysis			*N*	MAF_{GoNL}	After GCTA analysis		
						β	$SE_β$	*p*-value			β	$SE_β$	*p*-value
LDL	1	25,580,095	A	G	0.443	-0.047	0.008	7.28E-09	28797.3	0.430	-0.132	0.013	2.62E-23
LDL	1	25,724,005	C	T	0.494	0.012	0.007	1.15E-01	34179	0.505	0.180	0.014	5.52E-37
LDL	1	25,754,025	G	C	0.454	0.034	0.007	1.47E-06	36409.5	0.438	0.091	0.012	4.83E-15
LDL	1	55,493,584	A	C	0.242	-0.065	0.008	1.83E-14	35129.4	0.237	-0.066	0.008	6.05E-15
LDL	1	55,521,195	T	C	0.088	-0.075	0.014	2.56E-08	30971.3	0.076	-0.079	0.014	5.51E-09
LDL	1	62,981,440	G	A	0.371	-0.055	0.007	1.61E-14	38663.4	0.373	-0.060	0.007	5.40E-17
LDL	1	109,783,570	T	A	0.438	-0.010	0.007	1.31E-01	38859.4	0.418	-0.174	0.016	2.35E-27
LDL	1	109,784,938	C	T	0.458	0.042	0.007	7.86E-10	39626	0.454	0.167	0.016	7.25E-26
LDL	1	109,788,453	T	A	0.489	-0.001	0.007	8.90E-01	39406.9	0.500	-0.354	0.020	1.38E-71
LDL	1	109,818,530	C	T	0.238	-0.139	0.009	2.20E-57	32964.9	0.212	-0.169	0.010	1.82E-59
LDL	1	109,950,441	A	T	0.075	-0.097	0.014	9.69E-13	36138.6	0.080	-0.131	0.018	9.45E-14
LDL	1	234,849,339	G	A	0.477	-0.048	0.007	4.05E-12	38286.8	0.479	-0.048	0.007	2.93E-12
LDL	2	21,152,755	A	G	0.053	-0.135	0.017	4.43E-16	32511.3	0.043	-0.177	0.017	1.11E-24
LDL	2	21,183,334	C	A	0.061	0.023	0.016	1.38E-01	32155.9	0.058	0.144	0.022	2.04E-11
LDL	2	21,202,959	A	C	0.276	0.041	0.008	7.18E-08	38143.2	0.278	0.234	0.019	1.69E-35
LDL	2	21,213,193	T	C	0.273	0.057	0.008	2.66E-13	37392.1	0.291	0.327	0.020	1.84E-57
LDL	2	21,216,815	A	C	0.365	0.074	0.007	5.67E-25	37512	0.360	0.137	0.012	1.14E-31
LDL	2	21,217,490	T	C	0.479	0.020	0.007	2.91E-03	39156.3	0.520	-0.476	0.019	1.39E-142
LDL	2	21,244,000	T	G	0.451	0.082	0.007	7.43E-33	38190.2	0.474	0.269	0.018	9.47E-51
LDL	2	21,250,914	A	G	0.492	-0.057	0.007	5.86E-16	36837.3	0.450	0.113	0.014	4.27E-16
LDL	2	21,291,529	G	A	0.181	-0.114	0.009	1.41E-36	37527	0.138	-0.078	0.010	1.10E-15
LDL	2	44,063,098	C	G	0.070	-0.111	0.014	1.70E-15	35726.1	0.056	-0.093	0.014	4.88E-11
LDL	2	44,072,576	G	T	0.311	0.070	0.008	2.55E-18	32059.6	0.313	0.061	0.008	9.90E-14
LDL	5	74,656,539	C	T	0.415	0.066	0.007	1.44E-20	36784.9	0.396	0.066	0.007	2.74E-20
LDL	5	156,443,066	A	G	0.369	-0.047	0.007	3.51E-11	38391	0.346	-0.047	0.007	3.36E-11
LDL	6	160,985,526	A	G	0.075	0.123	0.016	4.71E-15	26519.1	0.079	0.123	0.016	4.35E-15
LDL	7	21,613,842	A	G	0.243	0.045	0.008	2.48E-08	37677.7	0.248	0.045	0.008	2.80E-08
LDL	8	9,184,231	G	A	0.092	-0.080	0.012	2.93E-11	37524.7	0.088	-0.080	0.012	2.69E-11
LDL	8	126,500,031	G	C	0.456	-0.062	0.007	1.00E-19	39312.3	0.441	-0.062	0.007	1.10E-19
LDL	8	145,069,534	T	C	0.360	0.041	0.007	1.07E-08	37800.2	0.338	0.041	0.007	1.16E-08
LDL	9	136,141,870	T	C	0.201	0.061	0.009	2.12E-12	38309.2	0.193	0.061	0.009	1.90E-12
LDL	11	61,557,803	C	T	0.339	-0.065	0.008	1.32E-17	34812.4	0.359	-0.065	0.008	1.46E-17
LDL	11	116,648,917	G	C	0.139	0.099	0.011	3.68E-21	34160.5	0.133	0.099	0.011	3.25E-21
LDL	16	52,310,527	A	G	0.195	0.044	0.009	7.04E-07	37284.7	0.193	0.049	0.009	4.40E-08
LDL	16	56,993,324	A	C	0.341	-0.057	0.007	8.64E-15	37697.1	0.329	-0.199	0.016	3.39E-36
LDL	16	56,994,212	C	T	0.277	0.034	0.008	1.71E-05	36223.3	0.280	-0.101	0.014	1.18E-13
LDL	16	56,994,894	A	G	0.492	0.007	0.007	3.42E-01	35858.9	0.533	0.153	0.014	7.98E-27
LDL	16	72,148,419	G	A	0.200	0.065	0.009	1.86E-13	37388.9	0.189	0.065	0.009	2.21E-13
LDL	17	67,081,278	G	A	0.034	0.135	0.023	6.67E-09	26820.2	0.030	0.135	0.023	6.71E-09
LDL	19	11,198,502	C	T	0.134	-0.198	0.010	1.76E-80	38321.5	0.117	-0.199	0.011	1.76E-73
LDL	19	11,227,480	A	C	0.428	-0.069	0.007	1.04E-23	41483	0.411	-0.080	0.007	3.69E-29
LDL	19	11,248,104	A	G	0.034	-0.084	0.019	1.43E-05	38962.9	0.029	-0.158	0.020	2.82E-15
LDL	19	11,283,516	A	G	0.065	-0.234	0.024	8.01E-22	13262.4	0.058	-0.150	0.026	4.09E-09
LDL	19	11,411,868	A	C	0.311	-0.033	0.008	1.34E-05	39155.4	0.309	-0.049	0.008	2.37E-10
LDL	19	18,285,944	A	G	0.276	0.037	0.008	2.50E-06	39847	0.270	0.060	0.008	3.22E-14
LDL	19	19,299,305	T	C	0.058	0.070	0.019	2.53E-04	24046.7	0.061	0.125	0.019	1.20E-10

LDL	19	19,447,352	G	A	0.052	-0.149	0.018	4.63E-17	31240.3	0.038	-0.141	0.018	3.30E-15
LDL	19	35,858,483	T	C	0.393	-0.003	0.009	7.04E-01	25087.9	0.396	-0.196	0.009	1.71E-95
LDL	19	36,324,324	G	A	0.194	0.000	0.011	9.83E-01	25143.4	0.210	-0.355	0.012	1.63E-194
LDL	19	36,624,359	A	G	0.319	0.002	0.009	8.32E-01	28810.2	0.327	-0.278	0.009	8.92E-193
LDL	19	37,947,185	T	C	0.016	0.048	0.033	1.45E-01	28961	0.015	1.350	0.036	1.71E-302
LDL	19	38,269,871	G	C	0.021	0.056	0.030	6.63E-02	26034.5	0.019	1.139	0.033	8.93E-267
LDL	19	38,683,266	C	A	0.029	0.016	0.025	5.26E-01	27834.9	0.027	0.920	0.027	9.19E-256
LDL	19	38,832,410	T	C	0.014	-0.066	0.035	5.99E-02	29870.6	0.024	-0.798	0.037	1.34E-103
LDL	19	39,120,462	A	G	0.012	-0.133	0.038	4.15E-04	27829	0.013	-1.969	0.047	0.00E+00
LDL	19	39,292,117	T	A	0.037	-0.033	0.022	1.21E-01	29496.3	0.038	1.227	0.028	0.00E+00
LDL	19	39,549,365	G	T	0.067	-0.021	0.017	2.30E-01	25462.4	0.046	-0.664	0.019	2.07E-260
LDL	19	40,080,355	C	G	0.018	0.051	0.029	7.98E-02	31633	0.015	1.137	0.032	2.00E-268
LDL	19	40,102,981	A	T	0.042	-0.033	0.021	1.14E-01	26979.1	0.038	-0.526	0.023	5.53E-115
LDL	19	40,260,983	G	C	0.015	0.013	0.040	7.57E-01	20463.5	0.014	1.193	0.043	3.57E-166
LDL	19	41,375,256	G	C	0.023	-0.161	0.036	6.01E-06	17215	0.012	-1.277	0.039	7.25E-237
LDL	19	41,402,057	G	A	0.007	-0.153	0.072	3.37E-02	14253	0.010	-1.261	0.075	6.71E-63
LDL	19	41,738,212	T	C	0.045	-0.010	0.024	6.78E-01	19781.3	0.048	-0.452	0.025	2.29E-70
LDL	19	42,016,880	C	T	0.308	0.000	0.008	9.75E-01	32228.5	0.316	0.220	0.009	2.82E-124
LDL	19	42,059,389	C	T	0.484	0.000	0.008	9.74E-01	32724.9	0.490	-0.067	0.009	9.82E-14
LDL	19	42,107,792	C	A	0.105	0.039	0.012	9.23E-04	38182.7	0.087	0.283	0.013	4.88E-106
LDL	19	42,424,510	T	C	0.063	-0.007	0.018	7.09E-01	24428	0.070	0.554	0.020	7.76E-164
LDL	19	43,908,757	C	G	0.024	-0.098	0.037	7.30E-03	15396.2	0.027	-0.819	0.041	1.09E-90
LDL	19	43,912,233	T	C	0.101	-0.032	0.012	9.52E-03	34733.7	0.107	-0.298	0.014	1.70E-95
LDL	19	43,960,771	T	C	0.117	-0.008	0.013	5.16E-01	28565.4	0.122	0.167	0.014	2.82E-33
LDL	19	43,976,583	T	C	0.118	-0.016	0.012	1.54E-01	35334.9	0.131	-0.264	0.012	2.14E-101
LDL	19	44,286,809	T	C	0.018	-0.003	0.029	9.24E-01	32696.2	0.025	-1.107	0.033	1.02E-253
LDL	19	44,352,665	T	C	0.290	-0.013	0.009	1.35E-01	31085.8	0.332	-0.301	0.010	5.69E-212
LDL	19	45,032,407	G	C	0.082	-0.019	0.014	1.79E-01	32102.6	0.080	-0.553	0.016	1.89E-268
LDL	19	45,302,504	A	G	0.025	-0.624	0.034	2.41E-75	16597.1	0.020	-0.681	0.042	1.62E-59
LDL	19	45,337,918	A	G	0.051	0.300	0.022	8.71E-42	20208.4	0.040	0.440	0.029	5.17E-53
LDL	19	45,359,706	A	G	0.295	0.130	0.008	3.98E-55	33597.2	0.270	0.235	0.010	1.71E-128
LDL	19	45,382,675	A	G	0.045	-0.453	0.019	4.01E-129	31796	0.038	-1.814	0.030	0.00E+00
LDL	19	45,389,596	A	G	0.042	-0.450	0.019	8.88E-123	32309.1	0.044	-1.209	0.036	5.02E-247
LDL	19	45,390,333	G	A	0.226	0.075	0.009	2.27E-17	34946.7	0.226	0.166	0.015	6.83E-29
LDL	19	45,404,972	C	T	0.474	-0.002	0.007	8.23E-01	40820.5	0.504	0.097	0.008	6.29E-31
LDL	19	45,418,486	A	G	0.035	0.399	0.031	2.70E-38	14736.7	0.027	0.500	0.038	1.03E-38
LDL	19	45,420,082	C	A	0.099	0.307	0.016	6.56E-86	21876.2	0.093	0.626	0.021	1.88E-192
LDL	19	45,422,160	G	C	0.195	0.227	0.010	5.20E-116	30922.9	0.200	0.441	0.017	2.83E-154
LDL	19	45,426,792	A	G	0.066	-0.504	0.018	3.33E-177	23940.4	0.062	-1.398	0.032	0.00E+00
LDL	19	45,436,244	C	T	0.402	0.117	0.012	1.58E-23	14626.3	0.412	0.233	0.013	2.45E-71
LDL	19	45,522,181	G	A	0.480	-0.007	0.008	3.82E-01	28182.4	0.527	-0.107	0.009	2.73E-31
LDL	19	45,585,167	T	C	0.432	-0.016	0.008	3.26E-02	34194.6	0.393	-0.073	0.008	4.39E-18
LDL	19	45,762,886	G	A	0.246	0.010	0.010	3.12E-01	28386.3	0.254	0.584	0.012	0.00E+00
LDL	19	46,320,218	A	G	0.011	-0.044	0.044	3.17E-01	23112.2	0.011	1.025	0.066	3.54E-55
LDL	19	46,514,441	A	G	0.264	0.020	0.009	2.81E-02	30090.3	0.269	-0.353	0.011	4.73E-237
LDL	19	46,546,840	A	C	0.018	-0.027	0.034	4.31E-01	23422.3	0.019	-2.136	0.055	0.00E+00
LDL	19	46,817,166	T	C	0.026	0.018	0.025	4.71E-01	30111	0.026	1.015	0.029	9.65E-272
LDL	19	46,824,904	T	A	0.145	0.031	0.012	1.21E-02	25400.5	0.163	0.370	0.014	2.80E-160
LDL	19	46,972,134	C	G	0.022	-0.008	0.027	7.55E-01	31314.9	0.026	-0.730	0.030	5.41E-129
LDL	19	47,262,197	T	C	0.025	-0.029	0.029	3.17E-01	23437.1	0.027	-0.684	0.032	1.32E-102
LDL	19	47,305,297	G	C	0.413	-0.013	0.009	1.29E-01	27658.1	0.394	-0.381	0.010	1.11E-305
LDL	19	47,305,760	G	T	0.055	0.016	0.020	4.33E-01	23619.9	0.069	1.143	0.025	0.00E+00
LDL	19	48,225,314	G	C	0.303	-0.011	0.010	3.11E-01	21225.5	0.289	-0.294	0.011	7.32E-147

LDL	19	48,533,330	C	T	0.030	-0.048	0.027	7.09E-02	23608.4	0.023	-0.921	0.030	3.73E-210
LDL	19	49,186,141	G	C	0.165	-0.018	0.010	6.42E-02	37415	0.163	-0.310	0.011	1.15E-183
LDL	19	49,337,237	G	A	0.093	0.001	0.017	9.33E-01	21183.6	0.095	0.624	0.018	5.18E-252
LDL	19	49,554,632	T	C	0.268	0.001	0.011	9.68E-01	19696.9	0.271	-0.067	0.012	9.60E-09
LDL	19	49,728,318	A	G	0.305	-0.017	0.009	5.44E-02	29522.4	0.285	-0.243	0.010	8.11E-133
LDL	19	49,877,060	T	C	0.008	0.183	0.066	5.34E-03	13838.5	0.008	0.432	0.068	2.42E-10
LDL	19	50,382,870	G	T	0.019	0.047	0.031	1.30E-01	26298.9	0.020	1.225	0.036	2.72E-252
LDL	19	50,747,533	C	T	0.037	-0.028	0.024	2.34E-01	23946.5	0.038	-0.747	0.027	1.22E-170
LDL	19	50,775,408	T	C	0.016	-0.026	0.040	5.11E-01	19078.8	0.020	-0.823	0.043	4.97E-80
LDL	19	50,779,151	T	C	0.261	0.012	0.009	2.06E-01	29674.7	0.268	0.266	0.010	2.21E-151
LDL	19	50,929,142	G	A	0.317	0.008	0.010	4.39E-01	22839.6	0.336	0.297	0.011	7.61E-162
LDL	19	51,579,634	T	C	0.019	0.025	0.034	4.53E-01	22391.6	0.024	-0.781	0.036	6.60E-103
LDL	19	51,801,506	T	G	0.023	0.007	0.030	8.23E-01	24158.1	0.022	0.786	0.033	4.58E-123
LDL	19	51,820,554	T	G	0.013	-0.036	0.042	3.82E-01	21282.4	0.020	-2.018	0.048	0.00E+00
LDL	19	52,148,617	G	A	0.373	0.002	0.010	8.42E-01	20323.3	0.359	-0.248	0.012	4.31E-94
LDL	19	52,162,237	G	A	0.343	0.007	0.008	4.06E-01	35339.4	0.326	-0.104	0.009	4.79E-29
LDL	19	52,252,712	A	G	0.014	-0.060	0.034	7.29E-02	30478.6	0.024	1.557	0.039	0.00E+00
LDL	19	52,285,858	G	A	0.300	0.003	0.009	7.58E-01	31969.3	0.289	0.204	0.009	2.93E-104
LDL	19	52,925,832	G	A	0.172	-0.007	0.010	4.65E-01	35478.1	0.176	-0.279	0.011	3.29E-151
LDL	19	53,069,703	A	G	0.047	0.003	0.018	8.67E-01	31763.2	0.051	0.748	0.022	2.23E-263
LDL	19	53,080,928	A	G	0.268	-0.019	0.008	2.62E-02	35886.1	0.241	-0.207	0.009	8.02E-117
LDL	19	53,103,677	T	G	0.019	0.062	0.029	3.59E-02	30708.7	0.026	0.208	0.032	4.47E-11
LDL	19	53,112,249	C	G	0.222	0.026	0.010	8.38E-03	28666.2	0.237	0.389	0.011	6.81E-252
LDL	19	53,135,014	A	G	0.266	0.003	0.009	7.06E-01	32073.4	0.249	-0.207	0.014	3.54E-52
LDL	19	53,149,057	A	C	0.298	0.000	0.008	9.60E-01	34437.3	0.288	0.420	0.013	3.97E-226
LDL	19	53,637,115	G	T	0.113	-0.018	0.014	2.02E-01	24761.2	0.119	-0.398	0.015	1.68E-152
LDL	19	53,981,103	G	T	0.080	-0.009	0.015	5.48E-01	28368.1	0.089	-0.560	0.017	7.91E-230
LDL	19	54,226,681	C	T	0.019	0.122	0.036	6.86E-04	19743.5	0.025	0.794	0.038	7.29E-97
LDL	19	54,228,064	A	C	0.300	-0.002	0.009	8.52E-01	31969.4	0.297	-0.185	0.009	1.73E-89
LDL	19	54,618,206	G	C	0.115	-0.003	0.012	7.90E-01	34273.5	0.113	0.314	0.013	1.48E-129
LDL	19	54,639,661	G	C	0.015	-0.009	0.039	8.11E-01	21662.3	0.025	-0.696	0.044	2.38E-57
LDL	19	55,013,318	C	T	0.220	0.008	0.009	3.63E-01	33320.2	0.208	0.286	0.010	2.14E-172
LDL	19	55,015,902	T	C	0.223	0.002	0.011	8.76E-01	25367.2	0.240	0.402	0.012	1.60E-252
LDL	19	55,454,861	T	C	0.043	-0.013	0.022	5.61E-01	25183.7	0.042	-0.801	0.024	1.13E-237
LDL	19	55,480,864	T	A	0.141	0.004	0.013	7.51E-01	24014.3	0.124	0.366	0.015	4.80E-139
LDL	19	55,513,879	G	C	0.016	0.022	0.033	5.14E-01	28456.8	0.023	-0.941	0.037	3.72E-142
LDL	19	56,201,817	G	A	0.103	0.004	0.012	7.60E-01	36486.4	0.098	-0.299	0.013	1.00E-117
LDL	19	56,438,192	C	T	0.006	0.069	0.062	2.70E-01	22898.4	0.005	2.023	0.069	4.80E-187
LDL	19	57,480,893	A	G	0.455	-0.013	0.007	6.05E-02	38759.8	0.444	-0.193	0.008	3.63E-142
LDL	19	58,190,130	A	G	0.035	0.000	0.024	9.87E-01	24980.5	0.036	0.742	0.026	7.16E-175
LDL	20	17,844,492	G	T	0.339	0.051	0.009	2.48E-09	28248.3	0.353	0.051	0.009	2.69E-09
LDL	20	39,218,023	G	C	0.384	-0.039	0.007	2.45E-08	39476.9	0.360	-0.040	0.007	9.90E-09
LDL	20	39,974,316	G	A	0.287	0.045	0.008	5.36E-09	37733.5	0.274	0.046	0.008	2.37E-09

Supplementary Table 4. Independent variants associated with TC after discovery phase.
EA: effect allele. NEA: non-effect allele. MAF and MAF_{GoNL}: the minor allele frequency of the effect allele in the discovery cohorts and in the GoNL reference panel. *N*: estimated effective sample size, determined by GCTA.

Trait	Chr	Position	EA	NEA	MAF	After METAL analysis			*N*	MAF_{GoNL}	After GCTA analysis		
						β	$SE_β$	*p*-value			β	$SE_β$	*p*-value
TC	1	25,724,005	C	T	0.493	0.009	0.008	2.31E-01	34227.7	0.505	0.133	0.013	4.14E-24
TC	1	25,768,937	A	G	0.462	-0.044	0.007	3.02E-09	39257.3	0.443	-0.144	0.012	1.33E-31
TC	1	55,493,584	A	C	0.244	-0.066	0.009	1.87E-13	35579.7	0.237	-0.070	0.009	5.27E-15
TC	1	62,980,161	A	G	0.372	-0.083	0.008	3.47E-28	39483.9	0.373	-0.085	0.008	7.36E-30
TC	1	109,818,530	C	T	0.240	-0.131	0.009	8.01E-47	34223.3	0.212	-0.131	0.009	5.50E-47
TC	1	234,849,339	G	A	0.479	-0.055	0.007	5.93E-14	39083.5	0.479	-0.055	0.007	4.61E-14
TC	2	21,014,883	C	T	0.018	-0.062	0.031	4.19E-02	30523.4	0.024	-0.211	0.034	7.58E-10
TC	2	21,152,755	A	G	0.053	-0.135	0.018	2.31E-14	32951.5	0.043	-0.174	0.018	1.64E-21
TC	2	21,183,334	C	A	0.061	0.021	0.017	1.96E-01	32707.9	0.058	0.140	0.023	5.32E-10
TC	2	21,202,959	A	C	0.278	0.040	0.008	6.97E-07	39223	0.278	0.221	0.020	2.25E-29
TC	2	21,213,193	T	C	0.275	0.054	0.008	4.10E-11	38467.6	0.291	0.301	0.021	7.87E-46
TC	2	21,216,815	A	C	0.366	0.072	0.008	4.27E-21	38417.7	0.360	0.127	0.012	2.54E-24
TC	2	21,217,490	T	C	0.477	0.024	0.007	1.04E-03	39908.8	0.520	-0.449	0.020	3.69E-113
TC	2	21,244,000	T	G	0.453	0.080	0.007	7.67E-28	38977.1	0.474	0.169	0.016	1.42E-25
TC	2	21,291,529	G	A	0.182	-0.109	0.010	2.31E-30	38227.2	0.138	-0.099	0.010	2.90E-23
TC	2	27,730,940	T	C	0.384	0.046	0.008	1.81E-09	37751	0.360	0.069	0.008	2.88E-19
TC	2	44,072,576	G	T	0.313	0.072	0.009	2.56E-17	33165.6	0.313	0.072	0.009	2.92E-17
TC	5	74,656,539	C	T	0.417	0.067	0.008	2.36E-19	37643.5	0.396	0.067	0.008	3.99E-19
TC	5	156,443,066	A	G	0.372	-0.050	0.008	2.18E-11	39229.2	0.346	-0.050	0.008	2.24E-11
TC	6	161,005,610	T	C	0.074	0.134	0.017	7.80E-16	27209.7	0.079	0.134	0.017	9.54E-16
TC	7	21,613,842	A	G	0.245	0.052	0.009	1.09E-09	38006.3	0.248	0.052	0.009	1.30E-09
TC	8	9,184,231	G	A	0.093	-0.101	0.013	2.88E-15	37885.9	0.088	-0.101	0.013	2.64E-15
TC	8	59,392,324	G	A	0.352	0.043	0.008	3.48E-08	38165.3	0.324	0.043	0.008	2.97E-08
TC	8	126,500,031	G	C	0.458	-0.069	0.007	5.63E-22	40031.3	0.441	-0.069	0.007	6.11E-22
TC	9	107,661,742	C	A	0.273	-0.058	0.008	3.77E-12	38042.9	0.246	-0.058	0.008	3.14E-12
TC	9	136,141,870	T	C	0.203	0.070	0.009	1.68E-14	38776.3	0.193	0.070	0.009	1.65E-14
TC	11	61,557,803	C	T	0.342	-0.071	0.008	8.10E-19	35790.4	0.359	-0.071	0.008	6.83E-19
TC	11	116,648,917	G	C	0.142	0.129	0.011	3.14E-31	34314.8	0.133	0.133	0.011	1.15E-32
TC	11	117,052,107	A	G	0.024	-0.164	0.030	2.83E-08	24889	0.020	-0.180	0.030	1.03E-09
TC	15	58,716,257	A	G	0.346	0.078	0.011	1.65E-12	18727.6	0.336	0.073	0.011	7.97E-11
TC	15	58,726,744	G	C	0.223	0.059	0.009	4.17E-11	38026.1	0.200	0.054	0.009	1.87E-09
TC	16	72,148,419	G	A	0.202	0.075	0.009	6.58E-16	38036.3	0.189	0.075	0.009	9.27E-16
TC	17	67,081,278	G	A	0.034	0.140	0.025	1.29E-08	26960.2	0.030	0.140	0.025	1.34E-08
TC	19	11,198,502	C	T	0.135	-0.196	0.011	1.42E-70	38952	0.117	-0.191	0.012	1.93E-61
TC	19	11,227,480	A	C	0.430	-0.065	0.007	5.65E-19	42297.5	0.411	-0.071	0.008	4.38E-21
TC	19	11,233,886	T	C	0.011	0.246	0.042	4.72E-09	28049.2	0.004	0.244	0.042	7.59E-09
TC	19	11,248,104	A	G	0.035	-0.066	0.021	1.26E-03	39417.2	0.029	-0.144	0.021	7.47E-12
TC	19	11,283,516	A	G	0.066	-0.246	0.026	1.16E-21	13540.8	0.058	-0.154	0.027	1.07E-08
TC	19	18,302,239	C	T	0.282	0.033	0.008	4.43E-05	41610.2	0.286	0.058	0.008	1.79E-12
TC	19	19,299,305	T	C	0.058	0.080	0.020	8.34E-05	24651.1	0.061	0.134	0.020	5.19E-11
TC	19	19,447,352	G	A	0.052	-0.160	0.019	2.28E-17	31245.2	0.038	-0.148	0.019	7.50E-15
TC	19	36,465,027	C	T	0.124	0.004	0.017	8.10E-01	18780.4	0.118	-0.302	0.018	1.57E-65
TC	19	36,488,326	G	A	0.049	-0.048	0.022	2.67E-02	25541.2	0.053	-0.359	0.022	2.50E-57
TC	19	36,624,359	A	G	0.320	-0.003	0.009	7.37E-01	29388.6	0.327	-0.215	0.010	1.15E-99
TC	19	37,226,329	C	A	0.010	-0.019	0.053	7.18E-01	20511.6	0.016	-0.825	0.056	1.73E-49
TC	19	37,417,530	C	G	0.060	-0.035	0.020	7.37E-02	25441.7	0.062	-0.629	0.022	6.42E-188

TC	19	38,683,266	C	A	0.030	0.039	0.026	1.37E-01	28099.5	0.027	0.832	0.029	3.25E-176
TC	19	38,790,635	C	A	0.124	0.019	0.015	1.98E-01	23594	0.135	-0.309	0.017	4.47E-73
TC	19	38,853,659	T	C	0.169	0.002	0.010	8.67E-01	37235.3	0.183	-0.135	0.012	3.34E-31
TC	19	38,990,336	T	C	0.052	0.037	0.018	4.22E-02	33829.3	0.046	0.390	0.020	1.17E-86
TC	19	39,043,586	A	C	0.110	0.004	0.015	8.06E-01	26266.9	0.103	0.431	0.016	2.39E-154
TC	19	39,538,538	T	G	0.077	0.007	0.020	7.18E-01	19039.8	0.072	0.506	0.022	3.61E-115
TC	19	39,609,053	C	T	0.051	0.029	0.019	1.25E-01	32944.1	0.051	0.341	0.020	2.37E-66
TC	19	39,862,881	T	C	0.447	0.012	0.008	1.42E-01	32497.9	0.435	0.072	0.009	1.15E-15
TC	19	39,955,515	T	C	0.094	0.049	0.015	7.39E-04	30928.3	0.088	0.499	0.016	9.04E-206
TC	19	40,102,981	A	T	0.042	-0.032	0.022	1.51E-01	27908.7	0.038	-0.523	0.024	3.07E-101
TC	19	40,281,184	A	G	0.199	0.007	0.010	5.01E-01	35400	0.177	-0.263	0.012	5.28E-102
TC	19	40,339,715	G	A	0.239	0.003	0.010	7.38E-01	32981.3	0.257	0.390	0.012	3.50E-215
TC	19	40,363,916	T	C	0.038	-0.037	0.025	1.34E-01	25094.2	0.040	-0.962	0.028	1.95E-262
TC	19	41,029,820	T	C	0.421	0.008	0.008	2.97E-01	38304.6	0.373	0.203	0.008	2.97E-130
TC	19	41,506,953	A	C	0.417	-0.009	0.009	3.12E-01	26330.2	0.394	0.166	0.010	1.64E-62
TC	19	41,582,932	A	G	0.075	0.009	0.020	6.36E-01	20531.5	0.069	-0.548	0.022	1.85E-138
TC	19	41,974,444	G	C	0.070	0.025	0.018	1.65E-01	26134.7	0.064	0.107	0.019	2.23E-08
TC	19	42,275,422	A	G	0.044	0.012	0.020	5.72E-01	31685.4	0.056	0.264	0.031	2.62E-17
TC	19	42,382,317	T	A	0.042	0.013	0.022	5.53E-01	27909.5	0.053	0.770	0.044	1.57E-67
TC	19	42,460,653	A	G	0.050	0.001	0.021	9.62E-01	26123.6	0.059	-0.384	0.034	2.86E-30
TC	19	43,896,450	A	G	0.136	-0.035	0.013	5.54E-03	29159.5	0.138	-0.285	0.014	3.16E-94
TC	19	44,460,205	G	A	0.188	0.006	0.011	6.03E-01	30024.7	0.204	-0.267	0.012	8.85E-104
TC	19	44,803,373	T	C	0.044	-0.030	0.023	1.97E-01	24873.3	0.054	-0.534	0.026	1.98E-97
TC	19	44,902,030	G	C	0.336	0.005	0.010	6.06E-01	24315	0.364	-0.314	0.013	1.97E-124
TC	19	44,927,778	C	T	0.447	0.020	0.010	5.38E-02	21098.5	0.455	0.202	0.013	1.98E-55
TC	19	45,033,193	T	C	0.408	-0.019	0.008	1.67E-02	34922.2	0.404	-0.067	0.009	1.83E-14
TC	19	45,040,191	T	C	0.101	0.033	0.019	8.44E-02	16750	0.086	0.599	0.022	1.00E-164
TC	19	45,302,504	A	G	0.026	-0.502	0.035	1.04E-47	18253.1	0.020	-1.020	0.044	2.83E-116
TC	19	45,328,379	A	T	0.184	0.144	0.013	4.57E-27	20439.8	0.175	0.187	0.016	1.07E-30
TC	19	45,345,283	C	A	0.012	0.224	0.046	1.35E-06	21150.7	0.013	0.974	0.052	8.14E-77
TC	19	45,351,516	G	C	0.056	0.220	0.022	1.28E-24	22506.6	0.045	0.591	0.029	2.09E-93
TC	19	45,359,706	A	G	0.296	0.115	0.009	6.58E-39	34106.9	0.270	0.185	0.011	5.20E-62
TC	19	45,369,203	A	G	0.049	-0.309	0.020	2.07E-52	28590.9	0.053	-0.268	0.030	3.88E-19
TC	19	45,370,571	A	G	0.285	-0.050	0.012	2.38E-05	19819.2	0.261	-0.074	0.013	3.00E-08
TC	19	45,382,675	A	G	0.045	-0.370	0.020	4.51E-79	32692.3	0.038	-1.253	0.031	0.00E+00
TC	19	45,418,486	A	G	0.036	0.329	0.032	2.51E-24	15348	0.027	0.437	0.040	2.39E-28
TC	19	45,422,946	G	A	0.197	0.197	0.011	9.09E-78	31419.8	0.200	0.394	0.024	1.69E-59
TC	19	45,423,636	A	G	0.385	0.052	0.009	7.64E-09	29471.1	0.351	0.351	0.011	1.90E-207
TC	19	45,423,944	T	C	0.021	-0.428	0.036	7.98E-33	20553	0.016	-0.908	0.051	1.12E-70
TC	19	45,426,792	A	G	0.068	-0.391	0.019	6.25E-96	24411.8	0.062	-0.974	0.033	1.44E-191
TC	19	45,428,234	A	G	0.171	0.205	0.012	5.59E-71	29189.9	0.164	0.449	0.027	1.80E-63
TC	19	45,742,011	G	C	0.281	-0.018	0.009	4.87E-02	34565.3	0.253	0.225	0.010	5.62E-105
TC	19	45,762,886	G	A	0.248	0.009	0.010	3.70E-01	29131	0.254	0.456	0.013	5.08E-281
TC	19	45,788,381	A	G	0.033	-0.082	0.030	6.03E-03	19483.9	0.029	-0.202	0.032	3.10E-10
TC	19	46,871,175	T	G	0.013	0.115	0.044	8.86E-03	22375.4	0.014	1.038	0.049	9.36E-101
TC	19	46,923,126	T	C	0.015	-0.100	0.034	3.64E-03	32471.7	0.015	-0.695	0.038	4.83E-76
TC	19	47,083,034	T	C	0.010	-0.062	0.060	3.04E-01	16003.8	0.012	-1.595	0.065	5.71E-133
TC	19	47,305,760	G	T	0.056	0.011	0.021	5.84E-01	24089.6	0.069	0.694	0.024	2.89E-182
TC	19	47,809,218	G	A	0.136	0.012	0.015	4.50E-01	20095.9	0.135	-0.398	0.017	4.22E-122
TC	19	48,375,023	A	G	0.013	-0.053	0.044	2.31E-01	21783.2	0.014	-0.471	0.047	1.47E-23
TC	19	49,090,982	C	T	0.271	0.005	0.009	5.87E-01	33846.2	0.235	0.180	0.010	2.27E-73
TC	19	49,124,991	C	G	0.068	-0.050	0.018	3.97E-03	28664.3	0.068	-0.156	0.019	6.16E-17
TC	19	49,717,516	C	T	0.136	0.006	0.012	6.45E-01	30709.4	0.147	0.340	0.014	4.13E-136

TC	19	50,055,223	G	A	0.053	0.002	0.022	9.39E-01	24070	0.050	0.129	0.023	3.70E-08
TC	19	50,750,419	C	T	0.193	-0.012	0.010	2.19E-01	36226.9	0.177	-0.290	0.011	2.36E-159
TC	19	50,929,119	G	A	0.317	0.003	0.010	7.69E-01	23622.4	0.335	0.368	0.012	1.61E-214
TC	19	51,132,097	C	T	0.083	-0.061	0.021	3.84E-03	16361	0.090	-0.595	0.023	1.39E-148
TC	19	51,179,712	A	G	0.495	0.018	0.007	1.62E-02	40427.8	0.486	0.063	0.008	5.98E-16
TC	19	51,301,057	C	T	0.032	-0.040	0.027	1.44E-01	23971.9	0.037	-0.567	0.030	8.23E-82
TC	19	51,334,355	T	C	0.053	-0.005	0.021	8.04E-01	26286.5	0.048	0.482	0.022	1.94E-103
TC	19	51,739,226	A	G	0.379	-0.010	0.009	2.62E-01	28387.4	0.390	-0.236	0.010	9.28E-125
TC	19	51,981,485	G	A	0.024	0.029	0.030	3.27E-01	26569.7	0.021	0.477	0.032	5.64E-51
TC	19	52,095,122	T	C	0.011	-0.060	0.048	2.07E-01	22863.4	0.012	-1.112	0.051	3.28E-104
TC	19	52,135,776	T	G	0.315	0.015	0.011	1.80E-01	19731.8	0.296	-0.191	0.018	9.49E-28
TC	19	52,147,969	T	C	0.326	0.021	0.011	5.43E-02	21184.1	0.318	0.433	0.017	3.41E-137
TC	19	52,204,328	C	A	0.026	0.006	0.029	8.27E-01	26649	0.025	0.625	0.031	7.24E-88
TC	19	52,513,871	T	A	0.004	0.003	0.075	9.65E-01	23842.9	0.006	-1.041	0.079	2.66E-39
TC	19	52,665,260	G	A	0.342	-0.001	0.009	9.14E-01	27838.5	0.367	-0.230	0.010	3.86E-109
TC	19	52,846,742	G	A	0.154	0.014	0.011	2.00E-01	37746.8	0.142	0.102	0.011	3.39E-20
TC	19	53,112,249	C	G	0.223	0.024	0.011	1.97E-02	28930.2	0.237	0.318	0.012	1.07E-164
TC	19	53,149,057	A	C	0.300	0.001	0.009	8.72E-01	34827.1	0.288	0.187	0.009	2.22E-87
TC	19	53,783,085	T	G	0.008	-0.028	0.051	5.80E-01	26813	0.007	1.038	0.055	1.36E-78
TC	19	53,836,862	A	G	0.443	-0.001	0.008	9.41E-01	35946.7	0.430	0.158	0.008	6.20E-79
TC	19	54,266,412	C	T	0.283	-0.017	0.012	1.47E-01	19280.2	0.258	-0.210	0.013	6.46E-60
TC	19	54,295,344	G	T	0.440	0.012	0.008	1.51E-01	31835.2	0.450	0.284	0.009	7.38E-199
TC	19	54,522,887	A	G	0.278	0.016	0.009	8.25E-02	33288.3	0.226	0.089	0.009	3.33E-21
TC	19	54,643,612	C	T	0.015	0.016	0.041	7.02E-01	22837.7	0.025	-0.567	0.043	2.80E-39
TC	19	54,661,559	A	G	0.321	-0.013	0.008	1.09E-01	37768.6	0.334	-0.220	0.009	1.58E-131
TC	19	54,753,823	G	A	0.077	-0.023	0.021	2.69E-01	18463.6	0.084	-0.619	0.022	5.82E-168
TC	19	54,827,040	C	A	0.141	0.033	0.016	3.58E-02	19027.1	0.128	0.519	0.017	5.07E-201
TC	19	55,021,466	A	G	0.200	0.007	0.011	5.14E-01	29612	0.213	0.382	0.012	5.16E-216
TC	19	55,029,298	T	A	0.182	-0.004	0.014	7.65E-01	20125.4	0.164	0.581	0.016	1.61E-285
TC	19	55,395,951	G	C	0.063	-0.043	0.020	3.31E-02	22591.9	0.055	-0.279	0.022	6.43E-38
TC	19	55,509,644	G	C	0.013	0.018	0.056	7.45E-01	13953.4	0.015	-0.814	0.058	8.62E-45
TC	19	55,698,183	C	T	0.101	-0.027	0.013	3.32E-02	37887.8	0.094	-0.289	0.014	6.97E-100
TC	19	56,990,010	T	C	0.212	-0.008	0.009	4.07E-01	39088.5	0.202	-0.183	0.010	4.21E-79
TC	20	17,845,072	G	C	0.164	0.062	0.011	5.04E-08	29822.7	0.145	0.062	0.011	4.60E-08
TC	20	39,960,342	A	G	0.407	0.046	0.008	7.79E-10	38467.2	0.398	0.046	0.008	7.42E-10

Supplementary Table 5. Independent variants associated with TG after discovery phase.
EA: effect allele. NEA: non-effect allele. MAF and MAF_{GoNL}: the minor allele frequency of the effect allele in the discovery cohorts and in the GoNL reference panel. *N*: estimated effective sample size, determined by GCTA.

Trait	Chr	Position	EA	NEA	MAF	After METAL analysis			*N*	MAF_{GoNL}	After GCTA analysis		
						β	$SE_β$	*p*-value			β	$SE_β$	*p*-value
TG	1	63,094,692	G	A	0.330	-0.016	0.003	8.81E-11	39668	0.369	-0.016	0.003	1.59E-10
TG	2	21,226,560	A	G	0.220	-0.023	0.003	2.93E-13	32984.5	0.223	-0.028	0.003	9.95E-19
TG	2	27,752,463	G	A	0.360	0.030	0.003	1.51E-30	34870.3	0.353	0.033	0.003	9.29E-36
TG	7	72,854,549	T	C	0.081	-0.040	0.005	2.76E-14	26192.3	0.088	-0.040	0.005	3.55E-14
TG	8	19,785,656	A	G	0.022	0.098	0.017	3.18E-09	9434.26	0.020	0.092	0.017	2.66E-08
TG	8	19,793,581	T	C	0.024	0.091	0.011	1.93E-17	20206.7	0.028	0.092	0.011	1.55E-17
TG	8	19,824,563	C	T	0.078	-0.053	0.005	3.64E-28	32831.8	0.119	-0.051	0.005	6.40E-26
TG	8	126,500,031	G	C	0.457	-0.018	0.002	2.10E-14	38228.8	0.441	-0.018	0.002	3.52E-14
TG	11	116,648,917	G	C	0.132	0.060	0.004	1.81E-49	28060	0.133	0.056	0.004	2.29E-41
TG	11	117,075,566	T	C	0.071	0.072	0.009	2.82E-16	10650.9	0.071	0.052	0.009	8.41E-09
TG	17	41,874,745	A	C	0.032	0.066	0.011	1.67E-09	15125	0.029	0.066	0.011	1.55E-09
TG	19	8,627,569	T	C	0.016	-0.143	0.025	8.02E-09	5947.35	0.007	-0.143	0.025	8.31E-09
TG	19	19,379,549	T	C	0.060	-0.052	0.006	3.36E-16	24617.2	0.064	-0.052	0.006	4.30E-16
TG	19	45,390,333	G	A	0.172	0.031	0.004	2.12E-15	27788.7	0.226	0.024	0.004	4.10E-10
TG	19	45,414,399	A	C	0.100	0.064	0.007	2.45E-22	14894.1	0.112	0.050	0.007	9.49E-14
TG	19	45,430,280	C	G	0.437	-0.028	0.004	1.56E-12	14479.2	0.439	-0.026	0.004	1.27E-10

Supplementary Table 6. Replication of the loci identified by Teslovich *et al.*[1] in the meta-analysis of the discovery cohorts.

β is the effect size of the effect allele per mmol per Liter.

Trait	SNP	Chr	Position	Teslovich *et al.* (2010)				This study				
				MAF	β	SE_β	*p*-value	MAF	β	SE_β	*p*-value	replicated?
HDL	rs3764261	16	56,993,324	0.320	-0.088	0.090	7.0E-380	0.330	-0.092	0.003	5.50E-176	y
LDL	rs4420638	19	45,422,946	0.170	-0.185	0.290	8.72E-147	0.195	-0.227	0.010	9.21E-116	y
TC	rs4420638	19	45,422,946	0.170	-0.177	0.320	5.20E-111	0.197	-0.197	0.011	9.09E-78	y
LDL	rs6511720	19	11,202,306	0.110	0.181	0.300	4.28E-117	0.130	0.198	0.011	1.11E-76	y
TC	rs6511720	19	11,202,306	0.110	0.184	0.340	6.65E-97	0.131	0.195	0.011	6.22E-67	y
LDL	rs629301	1	109,818,306	0.220	0.146	0.210	9.70E-171	0.236	0.134	0.009	1.55E-56	y
TG	rs964184	11	116,648,917	0.130	-0.186	0.480	6.71E-240	0.132	-0.060	0.004	1.81E-49	y
TC	rs629301	1	109,818,306	0.220	0.140	0.240	5.77E-131	0.239	0.126	0.009	8.82E-45	y
HDL	rs1532085	15	58,683,366	0.390	-0.038	0.080	2.92E-96	0.388	-0.044	0.003	8.00E-43	y
HDL	rs12678919	8	19,844,222	0.120	-0.058	0.120	9.71E-98	0.102	-0.066	0.005	3.31E-38	y
LDL	rs1367117	2	21,263,900	0.300	-0.105	0.190	4.48E-114	0.324	-0.088	0.007	1.16E-32	y
TC	rs964184	11	116,648,917	0.130	-0.121	0.290	6.21E-57	0.142	-0.129	0.011	3.14E-31	y
TC	rs1367117	2	21,263,900	0.300	-0.108	0.220	4.08E-96	0.326	-0.088	0.008	3.77E-29	y
TC	rs3850634	1	63,050,598	0.320	0.067	0.200	4.90E-41	0.365	0.081	0.008	2.27E-27	y
TG	rs1260326	2	27,730,940	0.410	-0.096	0.400	5.68E-133	0.379	-0.027	0.003	1.57E-26	y
LDL	rs964184	11	116,648,917	0.130	-0.074	0.270	1.47E-26	0.139	-0.099	0.011	3.68E-21	y
TG	rs12678919	8	19,844,222	0.120	0.150	0.650	1.50E-115	0.098	0.039	0.004	1.39E-20	y
LDL	rs12916	5	74,656,539	0.390	-0.063	0.180	5.12E-45	0.415	-0.066	0.007	1.44E-20	y
TC	rs12916	5	74,656,539	0.390	-0.074	0.200	8.79E-47	0.417	-0.067	0.008	2.36E-19	y
HDL	rs4420638	19	45,422,946	0.170	0.027	0.120	4.40E-21	0.187	0.039	0.004	2.41E-19	y
HDL	rs964184	11	116,648,917	0.130	0.039	0.110	5.21E-47	0.133	0.041	0.005	1.00E-18	y
LDL	rs4299376	2	44,072,576	0.300	-0.071	0.200	1.73E-47	0.311	-0.070	0.008	2.55E-18	y
HDL	rs7241918	18	47,160,953	0.170	0.034	0.100	2.73E-49	0.163	0.035	0.004	3.46E-18	y
TC	rs10401969	19	19,407,718	0.070	0.123	0.420	2.90E-38	0.080	0.120	0.014	4.67E-18	y
TC	rs4299376	2	44,072,576	0.300	-0.078	0.220	4.03E-45	0.313	-0.072	0.009	2.56E-17	y
TC	rs174550	11	61,571,478	0.340	0.046	0.200	2.08E-22	0.343	0.066	0.008	4.32E-17	y
LDL	rs10401969	19	19,407,718	0.070	0.081	0.380	6.69E-22	0.079	0.107	0.013	2.05E-16	y
LDL	rs174583	11	61,609,750	0.350	0.044	0.190	1.17E-21	0.349	0.059	0.007	2.83E-16	y
HDL	rs1883025	9	107,664,301	0.250	0.024	0.090	1.75E-33	0.250	0.028	0.004	4.92E-16	y
TC	rs2954022	8	126,482,621	0.460	0.060	0.190	5.02E-36	0.494	0.056	0.007	4.59E-15	y
TC	rs2126259	8	9,185,146	0.100	0.081	0.320	8.98E-24	0.106	0.095	0.012	9.39E-15	y
LDL	rs2954022	8	126,482,621	0.460	0.048	0.170	2.59E-29	0.493	0.051	0.007	2.57E-14	y
LDL	rs3850634	1	63,050,598	0.320	0.041	0.190	2.63E-18	0.363	0.053	0.007	6.84E-14	y
LDL	rs2479409	1	55,504,650	0.300	-0.052	0.220	1.93E-28	0.350	-0.058	0.008	1.94E-13	y
TG	rs1042034	2	21,225,281	0.220	0.066	0.450	1.36E-45	0.220	0.023	0.003	3.08E-13	y
TC	rs514230	1	234,858,597	0.480	0.035	0.200	5.37E-14	0.480	0.052	0.007	4.97E-13	y
TC	rs651007	9	136,153,875	0.210	-0.060	0.250	8.66E-21	0.217	-0.064	0.009	6.58E-13	y
TG	rs7811265	7	72,934,510	0.190	0.087	0.500	9.06E-59	0.177	0.025	0.004	1.21E-12	y
HDL	rs386000	19	54,792,761	0.200	-0.021	0.110	4.29E-16	0.203	-0.028	0.004	1.23E-12	y
TG	rs2954029	8	126,490,972	0.470	0.062	0.390	3.29E-55	0.485	0.017	0.002	3.13E-12	y
HDL	rs6065906	20	44,554,015	0.180	0.024	0.100	1.90E-22	0.194	0.027	0.004	3.20E-12	y
HDL	rs1042034	2	21,225,281	0.220	-0.023	0.090	1.22E-30	0.218	-0.025	0.004	3.87E-12	y
TC	rs2000999	16	72,108,093	0.200	-0.061	0.240	3.22E-24	0.195	-0.067	0.010	5.90E-12	y
TC	rs1883025	9	107,664,301	0.250	0.058	0.240	3.39E-27	0.260	0.058	0.009	7.68E-12	y
TG	rs10401969	19	19,407,718	0.070	0.086	0.820	1.61E-29	0.065	0.033	0.005	1.78E-11	y
TC	rs2479409	1	55,504,650	0.300	-0.051	0.240	3.84E-24	0.351	-0.055	0.008	3.64E-11	y
LDL	rs514230	1	234,858,597	0.480	0.029	0.180	9.38E-12	0.478	0.045	0.007	4.01E-11	y

TG	rs439401	19	45,414,451	0.360	0.061	0.440	1.14E-30	0.287	0.019	0.003	6.38E-11	y
LDL	rs649129	9	136,154,304	0.220	-0.053	0.210	7.85E-22	0.215	-0.054	0.008	8.13E-11	y
LDL	rs2000999	16	72,108,093	0.200	-0.052	0.220	1.75E-22	0.193	-0.060	0.009	8.17E-11	y
LDL	rs2126259	8	9,185,146	0.100	0.057	0.290	7.43E-15	0.105	0.075	0.012	8.64E-11	y
HDL	rs9987289	8	9,183,358	0.090	0.031	0.130	6.40E-25	0.088	0.034	0.005	9.15E-11	y
LDL	rs6882076	5	156,390,297	0.350	0.043	0.190	1.89E-22	0.385	0.045	0.007	3.16E-10	y
TG	rs13238203	7	72,129,667	0.040	0.087	1.340	1.13E-09	0.040	0.079	0.013	4.68E-10	y
TC	rs6882076	5	156,390,297	0.350	0.051	0.200	7.46E-28	0.387	0.047	0.008	7.59E-10	y
HDL	rs16942887	16	67,928,042	0.120	-0.033	0.110	8.39E-33	0.131	-0.027	0.005	1.47E-09	y
TC	rs1260326	2	27,730,940	0.410	-0.049	0.190	7.31E-27	0.384	-0.046	0.008	1.81E-09	y
HDL	rs4846914	1	230,295,691	0.400	0.016	0.070	3.66E-21	0.413	0.020	0.003	2.44E-09	y
TG	rs2131925	1	63,025,942	0.320	0.054	0.400	8.84E-43	0.333	0.015	0.003	2.45E-09	y
TC	rs4297946	20	39,811,275	0.470	-0.039	0.190	2.76E-17	0.483	-0.043	0.007	4.74E-09	y
TC	rs12027135	1	25,775,733	0.470	0.032	0.190	4.12E-11	0.462	0.042	0.007	5.08E-09	y
HDL	rs1800961	20	43,042,364	0.030	0.049	0.240	1.05E-15	0.037	0.052	0.009	8.65E-09	y
TC	rs1532085	15	58,683,366	0.390	-0.040	0.200	8.83E-20	0.399	-0.044	0.008	9.77E-09	y
LDL	rs12027135	1	25,775,733	0.470	0.028	0.180	1.24E-10	0.460	0.039	0.007	1.57E-08	y
HDL	rs174601	11	61,623,140	0.360	0.019	0.080	1.50E-22	0.342	0.018	0.003	1.90E-08	y
HDL	rs838880	12	125,261,593	0.310	-0.016	0.090	2.58E-14	0.346	-0.018	0.003	2.40E-08	y
HDL	rs1515100	2	227,128,917	0.370	-0.012	0.080	2.01E-09	0.381	-0.017	0.003	2.55E-08	y
HDL	rs10808546	8	126,495,818	0.440	-0.016	0.070	6.35E-19	0.463	-0.017	0.003	4.40E-08	y
TC	rs1030431	8	59,311,697	0.350	-0.033	0.200	8.79E-13	0.349	-0.042	0.008	8.16E-08	y
HDL	rs2925979	16	81,534,790	0.300	0.012	0.080	2.09E-11	0.332	0.018	0.003	1.19E-07	y
TG	rs261342	15	58,731,153	0.220	-0.033	0.450	2.42E-13	0.194	-0.018	0.003	1.20E-07	y
LDL	rs12670798	7	21,607,352	0.230	-0.033	0.200	6.88E-10	0.242	-0.043	0.008	2.41E-07	y
LDL	rs1030431	8	59,311,697	0.350	-0.025	0.180	3.86E-09	0.347	-0.038	0.007	2.56E-07	y
LDL	rs909802	20	39,936,815	0.470	-0.037	0.170	3.18E-19	0.485	-0.035	0.007	2.57E-07	y
LDL	rs11136341	8	145,043,543	0.400	-0.036	0.210	4.44E-13	0.381	-0.037	0.007	3.55E-07	y
HDL	rs643531	9	15,296,034	0.140	0.019	0.100	1.30E-13	0.151	0.021	0.004	6.82E-07	y
TC	rs7206971	17	45,425,115	0.490	-0.026	0.200	1.05E-08	0.451	-0.037	0.008	1.27E-06	y
HDL	rs1689800	1	182,168,885	0.350	0.012	0.080	3.18E-10	0.349	0.015	0.003	1.65E-06	y
HDL	rs4660293	1	40,028,180	0.230	0.012	0.090	3.99E-10	0.268	0.017	0.004	1.84E-06	y
HDL	rs13107325	4	103,188,709	0.070	0.022	0.160	7.20E-11	0.040	0.042	0.009	2.25E-06	y
TC	rs11136341	8	145,043,543	0.400	-0.035	0.240	8.96E-10	0.383	-0.036	0.008	3.20E-06	y
HDL	rs881844	17	37,810,218	0.340	0.013	0.080	2.84E-14	0.339	0.014	0.003	4.10E-06	y
HDL	rs4731702	7	130,433,384	0.480	-0.015	0.070	1.21E-15	0.485	-0.014	0.003	5.97E-06	y
HDL	rs3136441	11	46,743,247	0.150	-0.020	0.100	3.48E-18	0.143	-0.019	0.004	1.33E-05	y
TC	rs2290159	3	12,628,920	0.220	0.037	0.230	4.21E-09	0.227	0.036	0.009	3.40E-05	y
TC	rs2277862	20	34,152,782	0.150	0.031	0.270	3.82E-10	0.146	0.044	0.011	5.34E-05	y
LDL	rs217386	7	44,600,695	0.430	0.030	0.190	4.25E-11	0.461	0.028	0.007	5.80E-05	y
TG	rs442177	4	88,030,261	0.410	0.025	0.380	8.65E-12	0.434	0.010	0.002	6.55E-05	y
TC	rs1800562	6	26,093,141	0.060	0.056	0.430	2.49E-08	0.064	0.064	0.016	7.88E-05	y
TC	rs1800961	20	43,042,364	0.030	0.123	0.660	5.72E-13	0.038	0.086	0.022	1.01E-04	y
TC	rs2737229	8	116,648,565	0.300	0.029	0.210	2.45E-08	0.312	0.031	0.008	1.28E-04	y
HDL	rs12328675	2	165,540,800	0.130	-0.018	0.120	2.72E-10	0.125	-0.017	0.005	1.51E-04	y
LDL	rs1800562	6	26,093,141	0.060	0.057	0.390	6.07E-10	0.063	0.057	0.015	1.81E-04	y
HDL	rs605066	6	139,829,666	0.420	0.010	0.080	2.55E-08	0.438	0.011	0.003	2.01E-04	y
HDL	rs181362	22	21,932,068	0.200	0.012	0.090	1.11E-08	0.213	0.013	0.004	2.15E-04	y
HDL	rs4148008	17	66,875,294	0.320	0.011	0.080	1.79E-10	0.318	0.012	0.003	2.25E-04	y
TC	rs2072183	7	44,579,180	0.250	-0.052	0.290	3.22E-11	0.233	-0.035	0.010	2.39E-04	y
TC	rs2807834	1	220,970,593	0.320	0.036	0.220	4.90E-13	0.321	0.029	0.008	2.61E-04	y
TG	rs10195252	2	165,513,091	0.400	0.022	0.380	1.63E-10	0.432	0.009	0.003	2.62E-04	y
TC	rs7515577	1	93,009,438	0.210	0.031	0.240	2.78E-08	0.233	0.032	0.009	2.64E-04	y

HDL	rs2814944	6	34,552,797	0.160	0.013	0.100	3.81E-09	0.159	0.015	0.004	3.03E-04	y
TC	rs7239867	18	47,164,717	0.170	0.050	0.260	2.03E-19	0.171	0.036	0.010	3.47E-04	n
TC	rs492602	19	49,206,417	0.490	-0.033	0.210	2.01E-10	0.490	-0.026	0.007	3.82E-04	n
HDL	rs3741414	12	57,844,049	0.240	-0.012	0.090	1.64E-08	0.266	-0.012	0.004	9.12E-04	n
TC	rs3764261	16	56,993,324	0.320	-0.043	0.230	6.67E-14	0.344	-0.025	0.008	1.21E-03	n
TC	rs9488822	6	116,312,893	0.350	0.031	0.200	1.69E-10	0.322	0.025	0.008	1.23E-03	n
LDL	rs2807834	1	220,970,593	0.320	0.028	0.200	5.62E-11	0.318	0.024	0.007	1.37E-03	n
TG	rs645040	3	135,926,622	0.220	0.024	0.450	2.52E-08	0.200	0.009	0.003	1.47E-03	n
TG	rs174546	11	61,569,830	0.340	-0.042	0.380	5.41E-24	0.316	-0.008	0.003	1.99E-03	n
TG	rs1321257	1	230,305,312	0.390	-0.030	0.380	2.09E-14	0.387	-0.008	0.003	2.42E-03	n
TC	rs11220463	11	126,248,211	0.110	-0.052	0.330	2.12E-11	0.145	-0.032	0.011	2.89E-03	n
HDL	rs7255436	19	8,433,196	0.470	0.012	0.080	3.25E-08	0.472	0.009	0.003	3.23E-03	n
TG	rs4810479	20	44,545,048	0.240	-0.037	0.420	4.69E-18	0.240	-0.008	0.003	3.68E-03	n
LDL	rs11220462	11	126,243,952	0.140	-0.051	0.260	1.20E-15	0.148	-0.029	0.010	4.19E-03	n
TC	rs2255141	10	113,933,886	0.300	-0.030	0.200	2.03E-10	0.292	-0.023	0.008	5.06E-03	n
HDL	rs4765127	12	124,460,167	0.340	-0.011	0.080	2.89E-10	0.324	-0.009	0.003	5.30E-03	n
LDL	rs11153594	6	116,354,591	0.410	0.023	0.180	2.95E-09	0.385	0.019	0.007	6.73E-03	n
TC	rs2814982	6	34,546,560	0.110	0.048	0.330	4.68E-11	0.124	0.032	0.012	6.80E-03	n
TG	rs12310367	12	124,486,678	0.340	0.027	0.410	1.21E-08	0.321	0.007	0.002	7.50E-03	n
TG	rs1495743	8	18,273,300	0.220	-0.033	0.420	4.11E-14	0.232	-0.008	0.003	7.89E-03	n
TC	rs1961456	8	18,255,709	0.320	-0.028	0.210	1.68E-09	0.315	-0.021	0.008	8.12E-03	n
HDL	rs17145738	7	72,982,874	0.120	-0.015	0.120	1.19E-09	0.128	-0.012	0.005	9.66E-03	n
LDL	rs3757354	6	16,127,407	0.220	0.037	0.210	1.16E-11	0.222	0.022	0.009	9.74E-03	n
HDL	rs7134594	12	110,000,193	0.470	0.011	0.070	6.88E-15	0.480	0.008	0.003	1.02E-02	n
TC	rs2902940	20	39,091,487	0.290	0.036	0.210	6.08E-11	0.311	0.021	0.008	1.04E-02	n
TG	rs7205804	16	57,004,889	0.450	0.032	0.380	1.15E-12	0.436	0.006	0.002	1.06E-02	n
TC	rs1564348	6	160,578,860	0.170	-0.056	0.270	9.71E-17	0.164	-0.027	0.010	1.08E-02	n
HDL	rs2923084	11	10,388,782	0.170	0.011	0.100	4.62E-08	0.189	0.010	0.004	1.20E-02	n
TG	rs9686661	5	55,861,786	0.200	-0.028	0.490	1.32E-10	0.170	-0.009	0.004	1.25E-02	n
TC	rs10832963	11	18,664,241	0.290	0.027	0.220	2.52E-08	0.268	0.021	0.009	1.33E-02	n
LDL	rs2902941	20	39,091,514	0.330	0.025	0.190	1.11E-08	0.349	0.018	0.008	1.57E-02	n
HDL	rs12967135	18	57,849,023	0.230	0.011	0.090	6.58E-09	0.257	0.008	0.003	2.54E-02	n
LDL	rs1564348	6	160,578,860	0.170	-0.051	0.240	1.70E-17	0.162	-0.022	0.010	2.58E-02	n
TC	rs7941030	11	122,522,375	0.380	-0.025	0.190	1.52E-10	0.388	-0.017	0.008	2.77E-02	n
TG	rs2943645	2	227,099,180	0.370	0.021	0.380	2.35E-08	0.373	0.005	0.003	3.28E-02	n
HDL	rs2293889	8	116,599,199	0.410	0.011	0.080	5.77E-11	0.436	0.006	0.003	3.29E-02	n
HDL	rs4082919	17	76,377,482	0.480	0.010	0.080	4.98E-09	0.497	0.007	0.003	3.35E-02	n
TC	rs581080	9	15,305,378	0.180	0.041	0.260	3.08E-09	0.202	0.019	0.009	3.98E-02	n
HDL	rs7115089	11	122,530,591	0.370	-0.008	0.080	2.66E-08	0.374	-0.006	0.003	4.07E-02	n
TC	rs3757354	6	16,127,407	0.220	0.038	0.240	2.78E-09	0.224	0.018	0.009	5.28E-02	n
TG	rs2929282	15	44,245,931	0.050	-0.056	0.860	1.63E-11	0.041	-0.012	0.007	7.27E-02	n
TG	rs10761731	10	65,027,610	0.430	0.026	0.380	3.48E-12	0.432	0.004	0.003	9.06E-02	n
HDL	rs2652834	15	63,396,867	0.200	0.010	0.100	8.75E-09	0.187	0.006	0.004	1.01E-01	n
HDL	rs6450176	5	53,298,025	0.260	0.013	0.090	4.98E-08	0.257	0.006	0.003	1.03E-01	n
TG	rs11649653	16	30,918,487	0.400	0.023	0.390	3.35E-08	0.364	0.004	0.003	1.08E-01	n
TG	rs11613352	12	57,792,580	0.230	0.030	0.430	4.43E-10	0.274	0.004	0.003	1.18E-01	n
TC	rs11065987	12	112,072,424	0.420	0.025	0.200	6.77E-12	0.395	0.012	0.008	1.23E-01	n
HDL	rs737337	19	11,347,493	0.080	0.017	0.140	3.10E-09	0.083	0.008	0.006	1.34E-01	n
LDL	rs1129555	10	113,910,721	0.290	-0.028	0.200	2.14E-09	0.289	-0.011	0.008	1.63E-01	n
LDL	rs11065987	12	112,072,424	0.420	0.025	0.180	1.51E-09	0.392	0.010	0.007	1.74E-01	n
TG	rs5756931	22	38,546,033	0.400	0.017	0.380	3.82E-08	0.371	0.003	0.003	2.19E-01	n
TG	rs2412710	15	42,683,787	0.020	-0.077	1.490	1.87E-08	0.024	-0.010	0.009	2.61E-01	n
TG	rs2068888	10	94,839,642	0.470	0.025	0.380	2.38E-08	0.458	0.003	0.002	2.73E-01	n

LDL	rs7225700	17	45,391,804	0.350	0.023	0.180	3.92E-09	0.396	0.005	0.007	4.91E-01	n
TG	rs11776767	8	10,683,929	0.370	-0.022	0.390	1.30E-08	0.352	-0.002	0.003	5.16E-01	n
HDL	rs7134375	12	20,473,758	0.420	-0.010	0.080	3.84E-08	0.430	0.002	0.003	6.45E-01	n
LDL	rs2332328	14	24,883,058	0.480	-0.030	0.190	4.41E-11	0.480	0.001	0.007	8.45E-01	n

Supplementary Table 7. Replication of the loci identified by Willer *et al.*[2] in the meta-analysis of the discovery cohorts.

β is the effect size of the effect allele per mmol per Liter

Trait	SNP	Chr	Position	Willer *et al.* (2013)				This study				
				MAF	β	$SE_β$	*p*-value	MAF	β	$SE_β$	*p*-value	replicated?
HDL	rs11246602	11	51,512,090	0.150	0.034	-	2.00E-10	0.152	0.023	0.004	5.84E-08	y
HDL	rs12748152	1	27,138,393	0.090	-0.051	-	1.00E-15	0.081	-0.028	0.005	1.96E-07	y
LDL	rs1801689	17	64,210,580	0.040	0.103	-	1.00E-11	0.039	0.118	0.025	1.56E-06	y
HDL	rs499974	11	75,455,021	0.190	-0.026	-	1.00E-08	0.177	-0.018	0.004	2.80E-06	y
LDL	rs1250229	2	216,304,384	0.270	-0.024	-	3.00E-08	0.283	-0.031	0.008	8.18E-05	y
LDL	rs10490626	2	118,835,841	0.080	-0.051	-	2.00E-12	0.067	-0.057	0.015	1.21E-04	y
TC	rs4883201	12	9,082,581	0.120	-0.035	-	2.00E-09	0.101	-0.048	0.013	1.26E-04	y
LDL	rs2710642	2	63,149,557	0.350	-0.024	-	6.00E-09	0.335	-0.028	0.007	1.60E-04	y
TC	rs10102164	8	55,421,614	0.210	0.030	-	5.00E-11	0.207	0.034	0.009	1.89E-04	y
HDL	rs1047891	2	211,540,507	0.330	-0.027	-	9.00E-10	0.310	-0.013	0.004	2.28E-04	y
TG	rs4722551	7	25,991,826	0.200	0.029	-	9.00E-11	0.116	0.015	0.004	2.40E-04	y
TC	rs6831256	4	3,473,139	0.420	-0.022	-	1.00E-10	0.415	-0.027	0.008	3.07E-04	y
HDL	rs4142995	7	17,919,258	0.380	-0.026	-	9.00E-12	0.388	-0.011	0.003	3.20E-04	y
LDL	rs10102164	8	55,421,614	0.210	0.032	-	4.00E-11	0.205	0.031	0.009	3.42E-04	y
HDL	rs4650994	1	178,515,312	0.490	0.021	-	7.00E-09	0.472	0.010	0.003	3.58E-04	y
TC	rs1077514	1	23,766,233	0.150	-0.030	-	6.00E-09	0.161	-0.035	0.010	4.62E-04	y
LDL	rs314253	17	7,091,650	0.370	-0.024	-	3.00E-10	0.398	-0.027	0.008	4.93E-04	y
LDL	rs12748152	1	27,138,393	0.090	0.050	-	3.00E-12	0.086	0.043	0.013	5.54E-04	y
HDL	rs2290547	3	47,061,183	0.200	-0.030	-	4.00E-09	0.165	-0.015	0.005	8.67E-04	n
HDL	rs17695224	19	52,324,216	0.260	-0.029	-	2.00E-13	0.264	-0.011	0.003	9.45E-04	n
HDL	rs12145743	1	156,700,651	0.340	0.020	-	2.00E-08	0.365	0.011	0.003	9.68E-04	n
TC	rs314253	17	7,091,650	0.370	-0.023	-	3.00E-10	0.398	-0.026	0.008	1.05E-03	n
LDL	rs17404153	3	132,163,200	0.140	-0.034	-	2.00E-09	0.113	-0.037	0.012	1.14E-03	n
HDL	rs13326165	3	52,532,118	0.210	0.029	-	9.00E-11	0.198	0.013	0.004	1.49E-03	n
TC	rs10490626	2	118,835,841	0.080	0.042	-	6.00E-09	0.067	0.048	0.016	1.90E-03	n
TC	rs970548	10	46,013,277	0.260	-0.026	-	8.00E-09	0.272	-0.026	0.009	1.94E-03	n
TC	rs9376090	6	135,411,228	0.280	-0.025	-	3.00E-09	0.273	-0.025	0.008	2.24E-03	n
TC	rs7640978	3	32,533,010	0.090	-0.038	-	1.00E-08	0.078	-0.042	0.014	2.33E-03	n
TC	rs10904908	10	17,260,290	0.430	0.025	-	3.00E-11	0.434	0.022	0.007	2.78E-03	n
LDL	rs7640978	3	32,533,010	0.090	-0.039	-	1.00E-08	0.078	-0.039	0.013	2.79E-03	n
TC	rs4530754	5	122,855,416	0.460	-0.023	-	2.00E-09	0.469	-0.022	0.007	2.80E-03	n
TC	rs11694172	2	203,532,304	0.250	0.028	-	2.00E-09	0.261	0.025	0.008	2.93E-03	n
TG	rs7248104	19	7,224,431	0.420	-0.022	-	5.00E-10	0.399	-0.007	0.002	3.65E-03	n
TG	rs12748152	1	27,138,393	0.090	0.037	-	1.00E-09	0.080	0.014	0.005	4.18E-03	n
HDL	rs998584	6	43,757,896	0.490	-0.026	-	2.00E-11	0.462	-0.009	0.003	4.30E-03	n
LDL	rs6831256	4	3,473,139	0.420	0.025	-	2.00E-08	0.413	0.020	0.007	4.33E-03	n
HDL	rs731839	19	33,899,065	0.350	-0.022	-	3.00E-09	0.329	-0.009	0.003	4.77E-03	n
HDL	rs4983559	14	105,277,209	0.400	0.020	-	1.00E-07	0.397	0.011	0.004	5.01E-03	n
HDL	rs1121980	16	53,809,247	0.430	-0.020	-	7.00E-09	0.427	-0.009	0.003	6.03E-03	n
LDL	rs4722551	7	25,991,826	0.200	0.039	-	4.00E-14	0.152	0.029	0.011	6.53E-03	n
TC	rs4722551	7	25,991,826	0.200	0.023	-	7.00E-09	0.154	0.031	0.011	6.96E-03	n
LDL	rs4530754	5	122,855,416	0.460	-0.028	-	4.00E-12	0.467	-0.019	0.007	7.65E-03	n
LDL	rs3780181	9	2,640,759	0.080	-0.044	-	2.00E-09	0.068	-0.038	0.015	9.30E-03	n
LDL	rs4942486	13	32,953,388	0.480	0.024	-	2.00E-11	0.495	0.018	0.007	9.94E-03	n
TC	rs3780181	9	2,640,759	0.080	-0.044	-	7.00E-10	0.069	-0.040	0.015	9.97E-03	n

HDL	rs3822072	4	89,741,269	0.460	-0.025	-	4.00E-12	0.438	-0.008	0.003	1.04E-02	n
TC	rs13315871	3	58,381,287	0.100	-0.036	-	4.00E-08	0.094	-0.032	0.013	1.26E-02	n
LDL	rs2328223	20	17,845,921	0.210	0.030	-	6.00E-09	0.180	0.026	0.011	1.81E-02	n
TG	rs998584	6	43,757,896	0.490	0.029	-	3.00E-15	0.464	0.006	0.002	1.90E-02	n
HDL	rs12801636	11	65,391,317	0.230	0.024	-	3.00E-08	0.227	0.009	0.004	2.02E-02	n
HDL	rs702485	7	6,449,272	0.450	0.024	-	7.00E-12	0.438	0.007	0.003	2.18E-02	n
TG	rs1936800	6	127,436,064	0.490	-0.020	-	3.00E-08	0.492	-0.005	0.002	2.35E-02	n
TG	rs6831256	4	3,473,139	0.420	0.026	-	2.00E-12	0.401	0.005	0.002	2.52E-02	n
HDL	rs17173637	7	150,529,449	0.120	-0.036	-	2.00E-08	0.105	-0.011	0.005	2.73E-02	n
TG	rs1832007	10	5,254,847	0.180	-0.033	-	2.00E-12	0.155	-0.007	0.003	3.28E-02	n
HDL	rs2013208	3	50,129,399	0.500	0.025	-	9.00E-12	0.494	0.006	0.003	3.29E-02	n
LDL	rs11563251	2	234,679,384	0.120	0.034	-	5.00E-08	0.102	0.026	0.012	3.49E-02	n
HDL	rs970548	10	46,013,277	0.260	0.026	-	2.00E-10	0.260	0.007	0.004	3.79E-02	n
TC	rs138777	22	35,711,098	0.360	0.021	-	5.00E-08	0.372	0.016	0.008	4.30E-02	n
TC	rs2758886	6	39,250,837	0.300	0.023	-	3.00E-08	0.304	0.016	0.008	5.14E-02	n
TC	rs2287623	2	169,830,155	0.410	0.027	-	4.00E-12	0.399	0.015	0.008	5.23E-02	n
TC	rs2030746	2	121,309,488	0.400	0.020	-	4.00E-08	0.411	0.014	0.008	6.06E-02	n
TG	rs8077889	17	41,878,166	0.220	0.025	-	1.00E-08	0.195	0.006	0.003	6.11E-02	n
HDL	rs2606736	3	11,400,249	0.390	0.025	-	5.00E-08	0.361	0.006	0.003	6.90E-02	n
HDL	rs6805251	3	119,560,606	0.390	0.020	-	1.00E-08	0.368	0.006	0.003	7.09E-02	n
TG	rs1121980	16	53,809,247	0.430	-0.021	-	3.00E-08	0.424	-0.005	0.003	7.36E-02	n
HDL	rs10019888	4	26,062,990	0.180	-0.027	-	5.00E-08	0.154	-0.007	0.004	7.49E-02	n
TC	rs11563251	2	234,679,384	0.120	0.037	-	1.00E-09	0.102	0.022	0.013	8.90E-02	n
LDL	rs364585	20	12,962,718	0.380	-0.025	-	4.00E-10	0.403	-0.012	0.007	9.57E-02	n
LDL	rs2030746	2	121,309,488	0.400	0.021	-	9.00E-09	0.409	0.012	0.007	1.03E-01	n
TC	rs4253772	22	46,627,603	0.110	0.032	-	1.00E-08	0.105	0.019	0.013	1.31E-01	n
LDL	rs267733	1	150,958,836	0.160	-0.033	-	5.00E-09	0.173	-0.014	0.010	1.34E-01	n
HDL	rs1936800	6	127,436,064	0.490	0.020	-	3.00E-10	0.485	0.004	0.003	1.35E-01	n
TC	rs1997243	7	1,083,777	0.160	0.033	-	3.00E-10	0.186	0.012	0.010	2.25E-01	n
LDL	rs4253772	22	46,627,603	0.110	-0.031	-	3.00E-08	0.103	-0.013	0.012	2.69E-01	n
HDL	rs2602836	4	100,014,805	0.440	0.019	-	5.00E-08	0.432	0.003	0.003	3.10E-01	n
TG	rs3198697	16	15,129,940	0.430	-0.020	-	2.00E-08	0.415	-0.003	0.003	3.34E-01	n
TG	rs38855	7	116,358,044	0.470	-0.019	-	2.00E-08	0.467	-0.002	0.002	4.06E-01	n
TG	rs731839	19	33,899,065	0.350	0.022	-	3.00E-09	0.319	0.002	0.003	4.32E-01	n
HDL	rs4917014	7	50,305,863	0.320	0.022	-	1.00E-08	0.333	0.002	0.003	5.92E-01	n
LDL	rs5763662	22	30,378,703	0.040	0.077	-	1.00E-08	0.017	0.014	0.028	6.21E-01	n
TC	rs11603023	11	118,486,067	0.420	0.022	-	1.00E-08	0.421	0.002	0.008	7.85E-01	n
HDL	rs17404153	3	132,163,200	0.140	0.028	-	5.00E-09	0.109	0.000	0.005	9.27E-01	n

Supplementary Table 8. Potential novel variants associated with HDL, LDL, TC and TG.

EA: effect allele. MAF, MAF_{GoNL} and MAF_{1kG}: the minor allele frequency of the effect allele in the discovery cohorts, in the GoNL reference panel and in the 1kG reference panel. *N*: estimated effective sample size, determined by GCTA.

Trait	SNP	Chr	Position	EA	MAF	MAF_{GoNL}	MAF_{1kG}	*N*	After METAL analysis			After GCTA analysis: Before exclusion of known loci			After GCTA analysis: After exclusion of known loci		
									β	SE_{β}	*p*-value	β_{joint}	$SE_{\beta joint}$	*p*-$value_{joint}$	β_{joint}	$SE_{\beta joint}$	*p*-$value_{joint}$
HDL	rs4752801	11	47,907,641	G	0.355	0.347	0.338	35600.6	-0.023	0.003	1.62E-12	-0.040	0.004	1.74E-21	-0.023	0.003	1.35E-12
HDL	rs149580368	17	41,874,745	A	0.036	0.029	0.015	25123.6	-0.075	0.010	4.23E-14	-0.078	0.010	5.72E-15	-0.075	0.010	5.41E-14
LDL	rs77542162	17	67,081,278	G	0.034	0.030	0.008	26862.2	0.135	0.023	6.67E-09	0.135	0.023	6.71E-09	0.135	0.023	6.71E-09
TC	rs77542162	17	67,081,278	G	0.034	0.030	0.008	26969.4	0.140	0.025	1.29E-08	0.140	0.025	1.34E-08	0.140	0.025	1.34E-08
TC	rs144984216	19	20,479,901	T	0.046	0.028	0.011	21622.9	-0.140	0.024	7.88E-09	-0.081	0.023	4.65E-04	-0.140	0.024	8.41E-09
TG	rs117162033	19	8,627,569	T	0.016	0.007	0.007	5940.1	-0.143	0.025	8.02E-09	-0.143	0.025	8.31E-09	-0.143	0.025	8.31E-09

Supplementary Table 9. The LD between the novel variants and the known loci for the same trait located on the same chromosome.

Trait	Novel variant			Known loci			Rsq
	SNP	Chr	Position	SNP	Chr	Position	
HDL	rs4752801	11	47,907,641	rs2923084	11	10,388,782	0
				rs3136441	11	46,743,247	0.015
				rs11246602	11	51,512,090	0.071
				rs174601	11	61,623,140	0.007
				rs12801636	11	65,391,317	0
				rs499974	11	75,455,021	0.001
				rs964184	11	116,648,917	0.006
				rs7115089	11	122,530,591	0.002
	rs149580368	17	41,874,745	rs881844	17	37,810,218	0.018
				rs4148008	17	66,875,294	0.001
				rs4082919	17	76,377,482	0.004
LDL	rs77542162	17	67,081,278	rs314253	17	7,091,650	0
				rs7225700	17	45,391,804	0
				rs1801689	17	64,210,580	0.001
TC	rs77542162	17	67,081,278	rs314253	17	7,091,650	0
				rs7206971	17	45,425,115	0
	rs144984216	19	20,479,901	rs6511720	19	11,202,306	0.001
				rs10401969	19	19,407,718	0.139
				rs4420638	19	45,422,946	0.003
				rs492602	19	49,206,417	0
TG	rs117162033	19	8,627,569	rs7248104	19	7,224,431	0.001
				rs10401969	19	19,407,718	0.003
				rs731839	19	33,899,065	0.004
				rs439401	19	45,414,451	0

Supplementary Table 10. Conditional analysis within the discovery cohorts.
Direction: ERF, LLS, NTR-NESDA, PREVEND, PROSPER, RS-I, RS-II and RS-III. EA: effect allele. NEA: non effect allelle.

Trait	SNP	Chr	Position	EA	NEA	Unadjusted analysis				Conditional analysis			
						β	$SE_β$	*p*-value	Direction	β	$SE_β$	*p*-value	Direction
HDL	rs4752801	11	47,907,641	G	A	-0.016	0.003	1.88E-06	- - - - - - - -	-0.018	0.004	8.66E-06	- - - - - - - -
HDL	rs149580368	17	41,874,745	A	C	-0.063	0.011	3.72E-08	- - - - - - - -	-0.067	0.011	2.57E-09	- + - - - - - -
LDL	rs77542162	17	67,081,278	G	A	0.141	0.035	6.29E-05	+ + + + + + + +	0.118	0.031	1.75E-04	+ + + + + + + +
TC	rs77542162	17	67,081,278	G	A	0.138	0.034	4.75E-05	+ + + + + + + +	0.108	0.033	1.16E-03	+ + - + + + + +
TC	rs144984216	19	20,479,901	T	C	-0.131	0.034	9.78E-05	+ - - - - - - -	-0.084	0.042	4.27E-02	+ - - - - - - -
TG	rs117162033	19	8,627,569	T	C	-0.109	0.025	1.46E-05	- - - - - - - -	-0.117	0.025	2.68E-06	- - + - - - - -

Supplementary Table 11. Results of the replication of the potential novel variants.
Direction: CHS, CROATIA-Korcula, CROATIA-Split, CROATIA-Vis, FamHS, FHS, GS, MESA Whites, ORCADES, PROSPER-Ireland, PROSPER-Schotland. EA: effect allele. NEA: non effect allelle. MAF: minor allele frequency. $MAF_{min/max}$: the minimum and maximum MAF in the separate cohorts.

Trait	SNP	Chr	Position	EA	NEA	MAF	SE_{MAF}	MAF_{min}	MAF_{max}	β	$SE_β$	*p*-value	Direction
HDL	rs4752801	11	47,907,641	G	A	0.362	0.020	0.390	0.313	-0.012	0.003	5.63E-05	- - - - - - - - - - -
HDL	rs149580368	17	41,874,745	A	C	0.023	0.004	0.015	0.032	-0.079	0.014	5.90E-09	? - + - - ? - - - - -
LDL	rs77542162	17	67,081,278	G	A	0.026	0.006	0.033	0.014	0.125	0.031	4.35E-05	+ ? ? ? + + ? + - + +
TC	rs77542162	17	67,081,278	G	A	0.027	0.006	0.033	0.014	0.095	0.028	6.61E-04	+ ? ? ? + + + + - + +
TC	rs144984216	19	20,479,901	T	C	0.025	0.005	0.016	0.035	-0.056	0.036	1.22E-01	? - - - ? + + + - - -
TG	rs117162033	19	8,627,569	T	C	0.021	0.004	0.012	0.026	-0.133	0.030	7.98E-06	? - ? + ? ? ? - - - -

Supplementary Table 12. Baseline characteristics for replication cohorts.

Cohort	HDL			LDL		
	N (% male)	Mean age (SD), years	Mean HDL-C (SD), in mmol L^{-1}	*N* (% male)	Mean age (SD), years	Mean LDL-C (SD), in mmol L^{-1}
CHS	3116 (39.7)	72.40 (5.4)	1.43 (0.4)	3079 (39.7)	72.41 (5.4)	3.36 (0.9)
Croatia Korcula	858 (35.8)	55.89 (14.0)	1.46 (0.3)	855 (35.7)	55.90 (14.0)	3.85 (1.0)
Croatia Split	489 (41.9)	49.03 (14.6)	1.38 (0.3)	477 (40.5)	49.10 (14.7)	3.82 (1.1)
Croatia Vis	919 (42.7)	55.93 (15.6)	1.11 (0.2)	917 (42.7)	55.94 (15.6)	3.23 (0.9)
FamHS	3438 (46.3)	51.09 (13.7)	1.29 (0.4)	3436 (46.3)	51.09 (13.7)	3.22 (0.9)
FHS	7030 (46.3)	37.48 (9.6)	1.37 (0.4)	6964 (45.9)	37.43 (9.6)	3.06 (0.9)
Generation Scotland	7067 (39.7)	50.48 (13.2)	1.49 (0.4)	-	-	-
MESA Whites	2491 (48.3)	62.82 (10.2)	1.35 (0.4)	2471 (48.0)	62.87 (10.2)	3.23 (0.8)
ORCADES	1736 (38.2)	51.69 (15.1)	1.50 (0.4)	1736 (38.2)	51.69 (15.1)	3.47 (0.9)
PROSPER-Scottish	2303 (49.1)	75.31 (3.4)	1.29 (0.4)	2303 (49.1)	75.31 (3.4)	3.82 (0.8)
PROSPER-Irish	1980 (45.0)	75.45 (3.3)	1.29 (0.4)	1980 (45.0)	75.45 (3.3)	3.70 (0.8)

Cohort	TC			TG		
	N (% male)	Mean age (SD), years	Mean TC (SD), in mmol L^{-1}	*N* (% male)	Mean age (SD), years	Mean TG (SD), in mmol L^{-1}
CHS	3119 (39.7)	72.40 (5.4)	5.49 (1.0)	3119 (39.7)	72.40 (5.4)	1.57 (0.8)
Croatia Korcula	861 (35.9)	55.87 (14.0)	5.98 (1.2)	857 (35.7)	55.91 (14.0)	1.43 (0.8)
Croatia Split	489 (41.9)	49.03 (14.6)	5.88 (1.3)	486 (41.6)	49.03 (14.6)	1.49 (0.9)
Croatia Vis	921 (42.8)	55.98 (15.6)	5.10 (0.9)	918 (42.7)	55.91 (15.6)	1.68 (0.9)
FamHS	3435 (46.3)	51.09 (13.7)	5.27 (1.0)	3368 (46.3)	51.09 (13.7)	1.56 (0.8)
FHS	7047 (46.3)	37.47 (9.6)	4.94 (0.9)	7045 (46.3)	37.47 (9.6)	1.13 (0.9)
Generation Scotland	7078 (39.8)	50.47 (13.2)	5.33 (1.0)	-	-	-
MESA Whites	2498 (48.1)	62.84 (10.2)	5.26 (0.9)	2502 (48.1)	62.84 (10.2)	1.78 (0.5)
ORCADES	1736 (38.2)	51.69 (15.1)	5.47 (1.1)	1736 (38.2)	51.69 (15.1)	1.09 (0.6)
PROSPER-Scottish	2303 (49.1)	75.31 (3.4)	5.70 (0.9)	2303 (49.1)	75.31 (3.4)	1.58 (0.7)
PROSPER-Irish	1980 (45.0)	75.45 (3.3)	5.62 (0.9)	1980 (45.0)	75.45 (3.3)	1.54 (0.7)

Cohort	Country of origin
CHS	European Americans
Croatia Korcula	Croatia
Croatia Split	Croatia
Croatia Vis	Croatia
FamHS	European Americans
FHS	European Americans

Generation Scotland	Scotland
MESA Whites	European Americans
ORCADES	United Kingdom
PROSPER-Scottish	Scotland
PROSPER-Irish	Ireland

Supplementary Table 13. The genomic control factor (λ) per trait per cohort after removing the SNPs with Rsq < 0.3, Rsq > 1.1 and expMAC < 10.

Cohort	HDL		LDL		TC		TG	
	# SNPs	λ	# SNPs	λ	# SNPs	λ	# SNPs	λ
ERF	7,734,540	1.11	7,730,618	1.15	7,734,967	1.16	7,724,667	1.13
Lifelines	12,040,098	1.09	12,040,548	1.1	12,040,549	1.09	12,026,821	1.08
LLS	9,978,402	1	9,955,359	0.99	10,002,471	0.99	-	-
NTR NESDA	7,716,345	1.05	7,715,940	1.05	7,716,424	1.04	7,716,527	1.01
PREVEND	11,160,676	1.03	11,130,565	1.02	11,184,087	1.01	11,161,890	1.02
PROSPER	8,790,308	1.01	8,786,035	1	8,787,472	1	8,786,962	1.01
RS-I	10,542,999	1.03	10,478,841	1.02	10,572,917	1.01	10,537,253	1.02
RS-II	9,718,603	1.01	9,683,704	1	9,717,829	1.01	9,691,451	1.01
RS-III	10,153,993	1.01	10,134,268	1.03	10,155,983	1.03	10,137,373	1.02

Supplementary Table 14. The number of SNPs after meta-analysis.

Number of SNPs	HDL	LDL	TC	TG
after METAL analysis	13,001,835	12,986,317	13,017,734	12,879,667
after METAL analysis in > 6 cohorts	8,736,495	8,725,604	8,734,336	8,022,728
after METAL analysis in > 6 cohorts and labelled accessible genome	8,376,524	8,365,919	8,374,408	7,697,441

Supplementary Note 1: The Genome of the Netherlands Consortium and the CHARGE lipids Working Groups.

The Genome of the Netherlands Consortium.

Analysis group: Morris A. Swertz[6,7] (Co-Chair), Laurent C. Francioli[1], Freerk van Dijk[6,7], Androniki Menelaou[1], Pieter B.T. Neerincx[6,7], Sara L. Pulit[1], Patrick Deelen[6,7], Clara C. Elbers[1], Pier Francesco Palamara[2], Itsik Pe'er[2,8], Abdel Abdellaoui[9], Wigard P. Kloosterman[1], Mannis van Oven[10], Martijn Vermaat[11], Mingkun Li[12], Jeroen F.J. Laros[11], Mark Stoneking[12], Peter de Knijff[13], Manfred Kayser[10], Jan H. Veldink[14], Leonard H. van den Berg[14], Heorhiy Byelas[6,7], Johan T. den Dunnen[11], Martijn Dijkstra[6,7], Najaf Amin[15], K. Joeri van der Velde[6,7], Jouke Jan Hottenga[9], Jessica van Setten[1], Elisabeth M. van Leeuwen[15], Alexandros Kanterakis[6,7], Mathijs Kattenberg[9], Lennart C. Karssen[15], Barbera D.C. van Schaik[16], Jan Bot17, Isaäc J. Nijman[1], David van Enckevort[18], Hailiang Mei[18], Vyacheslav Koval[19], Kai Ye[20,21], Eric-Wubbo Lameijer[21], Matthijs H. Moed[21], Jayne Y. Hehir-Kwa[22], Robert E. Handsaker[5,23], Shamil R. Sunyaev[4,5], Mashaal Sohail[4,5], Fereydoun Hormozdiari[24], Tobias Marschall[25], Alexander Schönhuth[25], Victor Guryev[26], Paul I.W. de Bakker[1,3-5] (Co-Chair);
Cohort collection and sample management group: P. Eline Slagboom[21], Marian B. Beekman[21], Anton J.M. de Craen[21], H. Eka D. Suchiman[21], Albert Hofman[15], Cornelia van Duijn[15], Dorret I. Boomsma[9], Gonneke Willemsen[9], Bruce H. Wolffenbuttel[27], Mathieu Platteel[6], Steven J. Pitts[28], Shobha Potluri[28], David R. Cox[28,34];
Whole-genome sequencing: Qibin Li[29], Yingrui Li[29], Yuanping Du[29], Ruoyan Chen[29], Hongzhi Cao[29], Ning Li[30], Sujie Cao[30], Jun Wang[29,31,32];
Ethical, Legal, and Social Issues: Jasper A. Bovenberg[33];
Steering committee: Cisca Wijmenga[6,7] (Principal Investigator), Morris A. Swertz[6,7], Cornelia M. van Duijn[15], Dorret I. Boomsma[9], P. Eline Slagboom[21], Gertjan B. van Ommen[11], Paul I.W. de Bakker[1,3-5]

[1] Department of Medical Genetics, University Medical Center Utrecht, Utrecht, The Netherlands. [2] Department of Computer Science, Columbia University, New York, NY, USA. [3] Department of Epidemiology, University Medical Center Utrecht, Utrecht, The Netherlands. [4] Division of Genetics, Brigham and Women's Hospital, Harvard Medical School, Boston, MA, USA. [5] Broad Institute of Harvard and MIT, Cambridge, MA, USA. [6] Department of Genetics, University Medical Center Groningen, University of Groningen, Groningen, The Netherlands. [7] Genomics Coordination Center, University Medical Center Groningen, University of Groningen, Groningen, The Netherlands. [8] Department of Systems Biology, Columbia University, New York, NY, USA. [9] Department of Biological Psychology, VU University Amsterdam, Amsterdam, The Netherlands. [10] Department of Forensic Molecular Biology, Erasmus Medical Center, Rotterdam, The Netherlands. [11] Leiden Genome Technology Center, Department of Human Genetics, Leiden University Medical Center, Leiden, The Netherlands. [12] Department of Evolutionary Genetics, Max Planck Institute for Evolutionary Anthropology, Leipzig, Germany. [13] Forensic Laboratory for DNA Research, Department of Human Genetics, Leiden University Medical Center, Leiden, The Netherlands. [14] Department of Neurology, University Medical Center Utrecht, Utrecht, The Netherlands. [15] Department of Epidemiology, Erasmus Medical Center, Rotterdam, The Netherlands. [16] Bioinformatics Laboratory, Department of Clinical Epidemiology, Biostatistics and Bioinformatics, Amsterdam Medical Center, Amsterdam, The Netherlands. [17] SURFsara, Science Park, Amsterdam, The Netherlands. [18] Netherlands Bioinformatics Centre, Nijmegen, The Netherlands. [19] Department of Internal Medicine, Erasmus Medical Center, Rotterdam, The Netherlands. [20] The Genome Institute, Washington University, St. Louis, MO, USA. [21] Section of Molecular Epidemiology, Department of Medical Statistics and Bioinformatics, Leiden University Medical Center, Leiden, The Netherlands. [22] Department of Human Genetics, Radboud University Nijmegen Medical Centre, Nijmegen, The Netherlands. [23] Department of Genetics, Harvard Medical School, Boston, MA, USA. [24] Department of Genome Sciences, University of Washington, Seattle, WA, USA. [25] Centrum voor Wiskunde en Informatica, Life Sciences Group, Amsterdam, The Netherlands. [26] European Research Institute for the Biology of Ageing, University Medical Center Groningen, University of Groningen, Groningen, The Netherlands. [27] Department of Endocrinology, University Medical Center Groningen, Groningen, The Netherlands. [28] Rinat-Pfizer Inc, South San Francisco, CA, USA. [29] BGI-Shenzhen, Shenzhen, China. [30] BGI-Europe, Copenhagen, Denmark. [31] Department of Biology, University of Copenhagen, Copenhagen, Denmark. [32] The Novo Nordisk Foundation Center for Basic Metabolic Research, University of Copenhagen, Copenhagen, Denmark. [33] Legal Pathways Institute for Health and Bio Law, Aerdenhout, The Netherlands. [34] Deceased.

CHARGE lipids Working Group.

Aaron Isaacs, Adrienne Cupples, Alanna C Morrison, Albert Hofman, Albert Smith, Alex Reiner , Ani Manichaikul, Aniko Sabo, Aron Y Joon, Audrey Chu, Ayse Demirkan, Bruce Psaty, Camille Breaux, Charles Kooperberg, Chris Carlson, Chris O'Donnell, Chunyu Liu, Cornelia van Duijn, Daniel Chasman, Elisabeth van Leeuwen, Eric Boerwinkle, Gail Davies, Gina M Peloso, Gudny Eiriksdottir, Hao Mei, Ian Deary, Ida Chen, Ingrid Borecki , Jacy R Crosby, James G. Wilson, Jari Lahti, Jeanette Andrews, Jeff O'Connell, Jennifer Brody, Jennifer Huffman, Jennifer Robinson, Jennifer Smith, Jerome

Rotter, Johanna Jakobsdottir, Jose Ordovas, Josh Bis, Kelli Ryckman, Ken Rice, Kurt Lohman, Lawrence Bielak, Leslie Lange, Lindsay Reynolds, Marcia Lobos, Mary Feitosa, Myriam Fornage, Paul Auer, Rachel Mackey, Rebecca Jackson, Sekar Kathiresan, Serkalem Demissie, Stephen Rich, Tamara Harris, Tarun Ahluwalia, Vilmundur Gudnason, Wei Peng and Yongmei Liu

Supplementary Methods.

Study descriptions.

Discovery cohorts.

Erasmus Rucphen Family (ERF) Study. The ERF study has been described in detail previously[3]. A total of approximately 3,000 participants descend from 22 couples who lived in the Rucphen region in The Netherlands in the 19th century. The 2,755 individuals with genotype data and lipid measurements were included in the current analysis.

Lifelines. LifeLines[4] is a multi-disciplinary prospective population-based cohort study examining in a unique three-generation design the health and health-related behaviours of 165,000 persons living in the North East region of The Netherlands. It employs a broad range of investigative procedures in assessing the biomedical, socio-demographic, behavioural, physical and psychological factors which contribute to the health and disease of the general population, with a special focus on multimorbidity and complex genetics. This study only includes the individuals of which both genotype and lipid measurements was available.

Leiden Longevity Study (LLS). The LLS has been designed to investigate biomarkers of healthy ageing and longevity[5] and has been described in detail previously[6]. It is a family-based study consisting of 1,671 offspring of 421 nonagenarian sibling pairs of Dutch descent, and their 744 partners.

Netherlands Twin Register and Netherlands Study of Depression and Anxiety (NTR-NESDA). The sample used in the analyses in this study consisted of 5,764 participants of the Netherlands Twin Register (NTR). NTR participants are ascertained because of the presence of twins or triplets in the family and consist of multiples, their parents, siblings and spouses. Twins are born in all strata of society and NTR represents a general sample from the Dutch population. Age ranged between 12 and 89 (median 39), and 62.4% was female[7,8].

The other 1,816 samples originated from the NESDA cohort with available phenotype data. NESDA is a longitudinal study focusing on the course and consequences of depression and anxiety disorders. Subjects for NESDA were recruited from three sources, namely the general population, mental health organizations and general practices. The vast majority of NESDA subjects is selected for depression and anxiety, but the sample also includes healthy controls without lifetime psychiatric disorders. Age ranged between 18 and 65 in NESDA (median 43), and the proportion of females was 66.1%[9]. For all analysis, we excluded one monozygotic twin per pair. Additional corrections for

family resemblance are analysis specific, and described where appropriate. Lipids were measured from fasting blood samples following standard protocols as described in Willemsen *et al*.[8,10].

Prevention of Renal and Vascular End stage Disease study (PREVEND). This is an ongoing prospective study investigating the natural course of increased levels of urinary albumin excretion and its relation to renal and cardiovascular disease. Details of the protocol have been described elsewhere[11] (www.prevend.org). Blood samples were obtained in the morning hours. Red blood cell measurements were performed at the 2nd visit (about 4.2 years from baseline).

Prospective Study of Pravastatin in the Elderly at Risk (PROSPER). A detailed description of the study has been published elsewhere[12-14]. PROSPER was a prospective multicenter randomized placebo-controlled trial to assess whether treatment with pravastatin diminishes the risk of major vascular events in elderly. Between December 1997 and May 1999, we screened and enrolled subjects in Scotland (Glasgow), Ireland (Cork), and the Netherlands (Leiden). Men and women aged 70-82 years were recruited if they had pre-existing vascular disease or increased risk of such disease because of smoking, hypertension, or diabetes. A total number of 5,804 subjects were randomly assigned to pravastatin or placebo. A large number of prospective tests were performed including Biobank tests and cognitive function measurements. A whole genome wide screening has been performed in the sequential PHASE project with the use of the Illumina 660K beadchip. Of 5,763 subjects DNA was available for genotyping. Only the Dutch samples were used for the discovery phase of this study (PROSPER-Dutch).

Rotterdam Study cohort I (RS-I). The Rotterdam Study is an ongoing prospective population-based cohort study, focused on chronic disabling conditions of the elderly. The study comprises an outbred ethnically homogenous population of Dutch Caucasian origin. The rationale of the study has been described in detail elsewhere[15]. In summary, 7,983 men and women aged 55 years or older, living in Ommoord, a suburb of Rotterdam, the Netherlands, were invited to participate in the first phase. Fasting blood samples were taken during the participant's third visit to the research center.

Rotterdam Study cohort II (RS-II). The Rotterdam Study cohort II prospective population-based cohort study comprises 3,011 residents aged 55 years and older from the same district of Rotterdam. The rationale and study designs of this cohort is similar to that of the RS-I[15]. The baseline measurements, including the fasting HDL measurements, took place during the first visit.

Rotterdam Study cohort III (RS-III). The Rotterdam Study cohort III prospective population-based cohort study comprised 3,932 residents aged 45 years and older from the same district of Rotterdam. The rationale and study designs of this cohort is similar to that of the RS-I[15]. The baseline measurements, including the fasting HDL measurements, took place during the first visit.

Replication cohorts.

Cardiovascular Health Study (CHS). The CHS is a population-based cohort study of risk factors for CHD and stroke in adults greater than or equal to 65 years years conducted across four field centers[16]. The original predominantly Caucasian cohort of 5,201 persons was recruited in 1989-1990 from random samples of the Medicare eligibility lists; subsequently, an additional predominantly African-American cohort of 687 persons were enrolled for a total sample of 5,888. DNA was extracted from blood samples drawn on all participants at their baseline examination in 1989-1990. In 2007-2008, genotyping was performed at the General Clinical Research Center's Phenotyping/Genotyping Laboratory at Cedars-Sinai using the Illumina 370CNV BeadChip system on 3,980 CHS participants who were free of CVD at baseline, consented to genetic testing, and had DNA available for genotyping.

CHS was approved by institutional review committees at each site, the subjects gave informed consent, and those included in the present analysis consented to the use of their genetic information for the study of cardiovascular disease.

CROATIA-Korcula, CROATIA-Split and CROATIA-Vis. The CROATIA-Vis study includes unselected adult participants who were recruited in a population-based study during 2003 and 2004 in the villages of Vis and Komiza on the Dalmatian island of Vis. All subjects visited the clinical research centre in the region where they were examined in person and where fasting blood was drawn. Biochemical and physiological measurements were performed, detailed genealogies reconstructed, questionnaire of lifestyle and environmental exposures collected, and blood samples stored for further analyses. CROATIA-Korcula participants were recruited in the same manner from the Dalmatian island of Korcula in 2007 and CROATIA-Split from the mainland Croatian city of Split in 2009-2010. All studies received appropriate ethical approval, and all participants gave informed consent.

Family Heart Study (FamHS). The collection of phenotypes and covariates as well as clinical examination have been previously described for the Family Heart Study[17]. In brief, the FamHS began in 1992 with the ascertainment of 1,200 families, half randomly sampled and half selected because of an excess of coronary heart disease (CHD) or risk factor abnormalities as compared with age- and sex-specific population rates. The families, with approximately 6,000 subjects, were sampled from four population-based parent studies: the Framingham Heart Study, the Utah Family Tree Study, and two centers for the Atherosclerosis Risk in Communities study (ARIC: Minneapolis, and Forsyth County, NC). The participants attended a clinic visit between the years 1994-1996 and a broad range of

phenotypes was assessed in the general domains of CHD, atherosclerosis, cardiac and vascular function, inflammation and hemostasis, lipids and lipoproteins, blood pressure, diabetes and insulin resistance, pulmonary function, diet, habitual physical activity, anthropometry, medical history and medication use. Approximately 8 years later, 2,756 European American (EA) subjects belonging to the 510 of the largest and most informative pedigrees were invited for a second clinical exam (2002-2004). The most important CHD risk factors were measured again, including lipids, parameters of glucose metabolism, blood pressure, anthropometry, and several biochemical and hematologic markers. In addition, a computed tomography examination provided measures of coronary and aortic calcification, and abdominal and liver fat burden. Medical history and medication use was updated. A total of 3,794 EA subjects, from the first clinic visit, participated in the current study.

Framingham Heart Study (FHS). The FHS funded by the National Heart Lung and Blood Institute, is an observational population-based cohort study composed of three generations of Framingham (MA) residents predominately of European descent. The Original cohort (N = 5,209) was enrolled in 1948. The children and spouses of the Original cohort comprise the Offspring cohort (N = 5,124), which was enrolled in 1971-1975[18]. The Third Generation(N = 4,095) consists mostly of the children of the Offspring cohort, and was enrolled in 2002 to 2005[19]. All participants were examined every 4-8 years. DNA for surviving participants was collected in the late 1990s and early 2000s (1995-2005). Cholesterol and genetic data from 3,464 Offspring subjects and 3,569 Third Generation subjects contribute to this paper.

Generation Scotland (GS). The Generation Scotland: Scottish Family Health Study (GS:SFHS) is a collaboration between the Scottish Universities and the NHS, funded by the Chief Scientist Office of the Scottish Government. GS:SFHS is a family-based genetic epidemiology cohort with DNA, other biological samples (serum, urine and cryopreserved whole blood) and socio-demographic and clinical data from ~24,000 volunteers, aged 18-98 years, in ~7,000 family groups. Participants were recruited across Scotland, with some family members from further afield, from 2006-2011. Most (87%) participants were born in Scotland and 96% in the United Kingdom or Ireland. The cohort profile has been published[20]. GS:SFHS operates under appropriate ethical approvals, and all participants gave written informed consent.

Multi-Ethnic Study of Atherosclerosis (MESA Whites). MESA is a study of the characteristics of subclinical cardiovascular disease (disease detected non-invasively before it has produced clinical signs and symptoms) and the risk factors that predict progression to clinically overt cardiovascular disease or progression of the subclinical disease. MESA researchers study a diverse, population-based sample of 6,814 asymptomatic men and women aged 45-84. Thirty-eight percent of

the recruited participants are white, 28% African-American, 22% Hispanic, and 12% Asian, predominantly of Chinese descent, as well as 2,128 additional individuals from 594 families recruited through MESA Family by utilizing the existing MESA framework, yielding 3,026 sibpairs divided between African Americans and Hispanic-Americans. Participants were recruited from six field centers across the United States: Wake Forest University, Columbia University, Johns Hopkins University, University of Minnesota, Northwestern University and University of California - Los Angeles. For current investigation, analysis of the MESA cohort was restricted to those participants who self-identified as White.

Orkney Complex Disease studies (ORCADES). The ORCADES is a family-based, cross-sectional community study of the genetics of complex traits, based in the Orkney Isles in Scotland[21].

Prospective Study of Pravastatin in the Elderly at Risk (PROSPER). For the replication only the Scottish (PROSPER-Scottish) and the Irish (PROSPER-Irish) samples of the PROSPER study were used. The full description of PROSPER can be found at the section of the discovery cohorts.

Genotyping and imputations.

Discovery cohorts.

In ERF genotyping was done on various Illumina and Affymetrix chips. QC was done separately for each chip. For most chips, the following QC criteria were applied: callrate > 0.98, per individual callrate > 0.96, Hardy-Weinberg equilibrium (HWE) p-value > $5 \cdot 10^{-8}$ and minor allele frequency (MAF) > 0.005. IBS checks, sex chromosome checks and ethnicity checks were also performed. The imputation to the Genome of the Netherlands reference panel, release 4 was performed with MaCH (1.0.18c) and Minimac (minimac-beta-2012-03-14).

The genotyping in Lifelines was done with the Illumina Human CytoSNP12 array. The following QC was applied after genotyping: HWE ≤ 0.0001, MAF < 0.01, callrate < 0.95. If the SNP is not present in the reference data set, has null alleles and/or has an allele frequency which is comparable to the reference (> 25% difference), the SNP is excluded prior to imputations. The imputation to the Genome of the Netherlands reference panel, release 4 was performed with MaCH (1.0.17) and Minimac (minimac-beta-2012.10.3).

The genotyping in LLS was done with the Illumina Human 660_Quad and the Illumina OmniExpress. The following exclusion criteria were applied: sample call rate < 0.95, SNP call rate < 0.95, HWE p-value < $1 \cdot 10^{-4}$ and MAF < 0.01. The imputation to the Genome of the Netherlands

reference panel, release 4 was performed with IMPUTE version 2.2.

Genotyping in the NTR and NESDA samples was done on multiple platforms for partly overlapping subsets of the total sample (Affymetrix, Santa Clara, CA, USA; Perlegen 5.0, Mountain View, CA, USA; Illumina 370, San Diego, CA, USA; Illumina 660, Illumina Omni Express 1 M and Affymetrix 6.0). Quality control was done within as well as between platforms. Imputation was done using MaCH (version 1.0.18) and Minimac (version 2012.10.9 beta) using the GoNL reference panel release 4.1. SNP removal criteria included MAF < 0.01, HWE p-value $< 1 \cdot 10^{-5}$ and call rate < 0.95. Samples were excluded in case of sex mismatch, genotype missing rate > 0.1 or Plink F inbreeding value was either > 0.10 or < -0.10 (heterozygosity). On the merged data, the HWE and MAF SNP filters were reapplied, as well as an imputation quality cutoff $R^2 < 0.30$.

Genotyping for PREVEND was performed on the Illumina CytoSNP12 v2 chip. Samples exclusions based on call rates < 0.95, gender mismatch, duplicate discordance and genetic similarity. Population stratification was assessed by principal component analysis (PCA) over the sample correlation matrix, based on 16,842 independent (LD-pruned) SNPs. Samples were excluded when for they diverged from the mean with at least 3 standard deviations (Z-score > 3) for the first 5 principal components. SNPs were excluded with a MAF of < 0.01, call rate < 0.95, or deviation from HWE (p-value $< 1 \cdot 10^{-5}$). The imputation to the Genome of the Netherlands reference panel, release 4 was performed with MaCH (1.0.18) and Minimac (2012.10.3) after the genome positions were converted from hg18 to hg19 using the UCSC LiftOver tool (*http://hgdownload.cse.ucsc.edu/admin/exe/linux.x86_64/liftOver*).

The genotyping in PROSPER-Dutch was done with Illumina Human 660_Quadv1. The following exclusion criteria were applied: SNP call rate < 0.95, MAF < 0.01, HWE p-value $< 1 \cdot 10^{-4}$ and sample call rate < 0.95. The imputation to the Genome of the Netherlands reference panel, release 4 was performed with IMPUTE version 2.2. The genotyping, QC and imputations of PROSPER-Dutch was done together with the samples of PROSPER-Scottish and PROSPER-Irish.

The first two cohorts of the Rotterdam Study were genotyped using the Illumina 550K chip, the third cohort was genotyped using the Illumina 610K and 660K chip. The following exclusions were applied to identify a final set of SNPs that was used in this study: MAF < 0.05, SNP callrate < 0.95 and/or HWE p-value $< 1 \cdot 10^{-7}$. The QC was done per cohort. Imputation per cohort to the Genome of the Netherlands reference panel, release 4 was performed with MaCH and Minimac.

Replication cohorts.

In CHS both genotyped (rs4752801) and imputed SNPs (rs149580368, rs77542162 and rs117162033) were used for the replication. In CHS, genotyping was performed at the General Clinical Research Center's Phenotyping/Genotyping Laboratory at Cedars-Sinai using the Illumina 370CNV BeadChip system. Genotypes were called using the Illumina BeadStudio software. The following exclusions were applied to identify a final set of 306,655 autosomal SNPs: SNP call rate < 0.97, HWE *p*-value < $1 \cdot 10^{-5}$, more than 2 duplicate errors or Mendelian inconsistencies (for reference CEPH trios), heterozygote frequency equal to 0, SNP not found in HapMap and sample call rate < 0.95. For the 3,271 white participants in this study, the cleaned genotypes from the Illumina CNV370 were merged with genotypes from ITMAT-Broad-CARe (IBC) Illumina iSELECT chip. The IBC chip contained 46,423 markers on a subset of 2861 participants. The genotypes from the two chips were merged into a final set of 359,592 unique SNPs and then updated to hg19 position using plink. MaCH was used to pre-phase the genotypes. The phased genotypes were imputed into a reference panel of 1,092 individual of multiple ethnicities from the Phase1 version3 haplotypes of 1000 Genomes project using Minimac. The final number of imputed SNPs on the autosomes was 38,050,714.

In CROATIA-Korcula, CROATIA-Split and CROATIA-Vis only imputed SNPs were used for the replication. Both CROATIA-Korcula and CROATIA-Split were genotyped with the Illumina HumanHap370CNV chip, the CROATIA-Vis was genotyped with the Illumina HumanHap300v1 chip. The QC applied prior to imputations was: individuals call rate: 0.97, SNP call rate: 0.98, MAF cutoff: 0.01, HWE *p*-value cutoff: $1 \cdot 10^{-6}$ and inbreeding coefficient: 0.2-0.8. Imputation per cohort to the 1000 Genomes project reference panel (version Phase 1 integrated release v3, april 2012, all populations) was performed with SHAPEIT2 and IMPUTE2.

In FamHS rs4752801 was genotyped, the other replication SNPs were imputed. The FamHS participants were genotyped on three Illumina platforms (HumMap550K, Human 6100-Quadv1, or Human 1M-Duov3). Quality control was performed before imputation and included assessment of Mendelian errors[22] and verification of reported pedigree relationships using GRR[23]. For the 1000 Genome reference panel imputation, SNPs were used if: call rate > 0.98; MAF > 0.01; no deviation from HWE *p*-value > $1 \cdot 10^{-6}$, allelic match with 1000 Genomes; and present in 1000 Genome reference panel genotyping. A total of 501,404 (550K), 530,979 (610K-Quadv1), and 910,456 (1M-Duov3) SNPs were included for the haplotype phasing and imputation using MaCH (version 1.0.16[24,25]). The reference phased haplotypes were the Cosmopolitan panel (including all races-version 2010-11 data freeze, 2012-03-04 haplotypes). The number of imputed gene dosages was about 38

million for EA subjects. To create the hybrid dataset, genotyped SNPs were strand oriented to the HapMap + strand. The original genotypes were converted to dosages. Genotyped SNPs were excluded from the hybrid dataset if a genotyped SNP was not in dbSNP or the mlinfo file from the 1000 Genome reference panel imputation. SNPs that were mapped to more than one location in the genome were excluded. Imputed gene dosages were used in the hybrid dataset for any genotyped SNP with a call rate < 0.98 or a MAF < 0.01. For the remaining genotyped SNPs common to all three genotyping platforms, genotyped dosage data was merged over imputed data in the mldose file whenever the measured genotype was available, with missing genotyped data kept as missing in the hybrid dataset and monomorphic SNPs removed. This process led to a hybrid dataset with 36,545,314 SNPs, of which 519,261 SNPs were genotyped and 36,026,053 SNPs were imputed.

In FHS only imputed SNPs were used for the replication. Samples of the FHS were genotyped with Affymetrix 250K Nsp and 250K Sty mapping arrays and the Affymetrix 50K supplemental gene-focused array. The QC criteria for variants used in the imputations was: HWE p-value $< 1 \cdot 10^{-7}$, call rate < 0.969, MAF < 0.005 and Mendelian errors < 1000. Imputation to the 1000 Genomes project reference panel (version Phase 1 integrated release v3, april 2012, all populations) was performed with MaCH (version 1.0.15).

In Generation Scotland only imputed SNPs were used for the replication. The samples were genotyped with the OMNI Express chip and with the Illumina HumanExome array. The QC criteria for variants on the OMNI express were: individual call rate 0.98, SNP call rate 0.98, MAF 0.01 and HWE p-value $1 \cdot 10^{-6}$. The QC criteria for variants on the Exome array were: individual call rate 0.99, SNP call rate 0.98, MAF 0.0001 and HWE p-value $1 \cdot 10^{-6}$. Prior to imputations both data sets were merged. Imputation to the 1000 Genomes project reference panel (version Phase 1 integrated release v3, april 2012, all populations) was performed with SHAPEIT and IMPUTE2.

In MESA Whites and MESA Family only imputed SNPs were used for the replication. MESA Whites and MESA Family samples were genotyped using the Affymetrix Genome-Wide Human SNP Array 6.0 (Santa Clara, California); for the current meta-analysis only self-reported Caucasian participants were analyzed. Sample exclusion criteria included heterozygosity > 0.53 and individual-level genotyping callrate < 0.95. Monomorphic SNPs were removed, and there was no filter on HWE or MAF. IMPUTE version 2.2.2 was used to perform imputation for the MESA SHARe Caucasian participants (chromosomes 1-23) using the 1000 Genomes haplotypes release Phase I integrated variant set (NCBI build 37, hg19) based on data freezes from 23 Nov 2010 (low-coverage whole-genome) and 21 May 2011 (high-coverage exome); phased haplotypes released March 2012 (v3).

In ORCADES only imputed SNPs were used for the replication. 890 samples of the ORCADES

cohort were genotyped with the Illumina Hap300 chip. The QC criteria for variants used in the imputations were: call rate < 0.97, FDR > 0.01, ethnic outliers, duplicates, gender mismatch, excess IBS incompatible with pedigree, MAF < 0.01 and HWE *p*-value $< 1 \cdot 10^{-6}$. Imputations to the 1000 Genomes project reference panel (version Phase 1 integrated release v3, april 2012, all populations) was performed with SHAPEIT (version 2, r644) and IMPUTE2 (version 2.2.2). 304 samples of the ORCADES cohort were genotyped with the Illumina Omni1 chip and an additional 1,073 samples were genotyped with the Illumina OmniX. QC exclusion criteria for variants genotyped with these chips prior to the imputations were: call rate < 0.97, ethnic outliers, duplicates, gender mismatch, excess IBS incompatible with pedigree, monomorphic SNPs and HWE *p*-value $< 1 \cdot 10^{-6}$. Data from both SNP arrays was merged after QC prior to the imputations. Imputations to the 1000 Genomes project reference panel (version Phase 1 integrated release v3, april 2012, all populations) was performed with SHAPEIT (version 2, r644) and IMPUTE2 (version 2.2.2).

In PROSPER-Scottish and PROSPER-Irish both imputed and genotyped SNPs were used for the replication. The genotyping in these cohorts was done with Illumina Human 660_Quadv1. The following exclusion criteria were applied: SNP call rate < 0.95, MAF < 0.01, HWE *p*-value $< 1 \cdot 10^{-4}$ and sample call rate < 0.95. The imputation to the 1000 Genomes project reference panel (version Phase 1 integrated release v3, april 2012, all populations) was performed with IMPUTE version 2.2. The genotyping, QC and imputations of these cohorts was done together with the samples of PROSPER-Dutch.

Supplementary References.

[1] Teslovich, T. M. *et al.* Biological, clinical and population relevance of 95 loci for blood lipids. *Nature* **466**, 707–713 (2010).

[2] Consortium, G. L. G. *et al.* Discovery and refinement of loci associated with lipid levels. *Nat Genet* **45**, 1274–1283 (2013).

[3] Pardo, L. M., MacKay, I., Oostra, B., van Duijn, C. M. & Aulchenko, Y. S. The effect of genetic drift in a young genetically isolated population. *Ann Hum Genet* **69**, 288–295 (2005).

[4] Stolk, R. P. *et al.* Universal risk factors for multifactorial diseases: LifeLines: a three-generation population-based study. *Eur J Epidemiol* **23**, 67–74 (2008).

[5] Westendorp, R. G. J. *et al.* Nonagenarian siblings and their offspring display lower risk of mortality and morbidity than sporadic nonagenarians: The Leiden Longevity Study. *J Am Geriatr Soc* **57**, 1634–1637 (2009).

[6] Schoenmaker, M. *et al.* Evidence of genetic enrichment for exceptional survival using a family approach: the Leiden Longevity Study. *Eur J Hum Genet* **14**, 79–84 (2006).

[7] Boomsma, D. I. *et al.* Netherlands Twin Register: from twins to twin families. *Twin Res Hum Genet* **9**, 849–857 (2006).

[8] Willemsen, G. *et al.* The Netherlands Twin Register biobank: a resource for genetic epidemiological studies. *Twin Res Hum Genet* **13**, 231–245 (2010).

[9] Penninx, B. W. J. H. *et al.* The Netherlands Study of Depression and Anxiety (NESDA): rationale, objectives and methods. *Int J Methods Psychiatr Res* **17**, 121–140 (2008).

[10] van Reedt Dortland, A. K. B. *et al.* Associations between serum lipids and major depressive disorder: results from the Netherlands Study of Depression and Anxiety (NESDA). *J Clin Psychiatry* **71**, 729–736 (2010).

[11] Hillege, H. L. *et al.* Microalbuminuria is common, also in a nondiabetic, nonhypertensive population, and an independent indicator of cardiovascular risk factors and cardiovascular morbidity. *J Intern Med* **249**, 519–526 (2001).

[12] Shepherd, J. *et al.* The design of a prospective study of Pravastatin in the Elderly at Risk (PROSPER). PROSPER Study Group. PROspective Study of Pravastatin in the Elderly at Risk. *Am J Cardiol* **84**, 1192–1197 (1999).

[13] Shepherd, J. *et al.* Pravastatin in elderly individuals at risk of vascular disease (PROSPER): a randomised controlled trial. *Lancet* **360**, 1623–1630 (2002).

[14] Trompet, S. *et al.* Replication of LDL GWAs hits in PROSPER/PHASE as validation for future (pharmaco)genetic analyses. *BMC Med Genet* **12**, 131 (2011).

[15] Hofman, A. *et al.* The Rotterdam Study: 2012 objectives and design update. *Eur J Epidemiol* **26**, 657–686 (2011).

[16] Fried, L. P. *et al.* The Cardiovascular Health Study: design and rationale. *Ann Epidemiol* **1**, 263–276 (1991).

[17] Higgins, M. *et al.* NHLBI Family Heart Study: objectives and design. *Am J Epidemiol* **143**, 1219–1228 (1996).

[18] Kannel, W. B., Feinleib, M., McNamara, P. M., Garrison, R. J. & Castelli, W. P. An investigation of coronary heart disease in families. The Framingham offspring study. *Am J Epidemiol* **110**, 281–290 (1979).

[19] Splansky, G. L. *et al.* The Third Generation Cohort of the National Heart, Lung, and Blood Institute's Framingham Heart Study: design, recruitment, and initial examination. *Am J Epidemiol* **165**, 1328–1335 (2007).

[20] Smith, B. H. *et al.* Cohort Profile: Generation Scotland: Scottish Family Health Study (GS:SFHS). The study, its participants and their potential for genetic research on health and illness. *Int J Epidemiol* **42**, 689–700 (2013).

[21] McQuillan, R. *et al.* Runs of homozygosity in European populations. *Am J Hum Genet* **83**, 359–372 (2008).

[22] Heath, S. C. Markov chain Monte Carlo segregation and linkage analysis for oligogenic models. *Am J Hum Genet* **61**, 748–760 (1997).

[23] Abecasis, G. R., Cherny, S. S., Cookson, W. O. & Cardon, L. R. GRR: graphical representation of relationship errors. *Bioinformatics* **17**, 742–743 (2001).

[24] Li, Y., Willer, C., Sanna, S. & calo Abecasis, G. Genotype imputation. *Annu Rev Genomics Hum Genet* **10**, 387–406 (2009).

[25] Li, Y., Willer, C. J., Ding, J., Scheet, P. & calo R Abecasis, G. MaCH: using sequence and genotype data to estimate haplotypes and unobserved genotypes. *Genet Epidemiol* **34**, 816–834 (2010).
